# Direct electrochemical oxidation of alcohols with hydrogen evolution in continuous-flow reactor

**DOI:** 10.1038/s41467-019-10928-0

**Published:** 2019-06-26

**Authors:** Dan Wang, Pan Wang, Shengchun Wang, Yi-Hung Chen, Heng Zhang, Aiwen Lei

**Affiliations:** The Institute for Advanced Studies (IAS), College of Chemistry and Molecular Sciences, Wuhan University, 430072 Wuhan, Hubei People’s Republic of China

**Keywords:** Flow chemistry, Electrocatalysis, Sustainability, Synthetic chemistry methodology

## Abstract

Alcohol oxidation reactions are widely used for the preparation of aldehydes and ketones. The electrolysis of alcohols to carbonyl compounds have been underutilized owing to low efficiency. Herein, we report an electrochemical oxidation of various alcohols in a continuous-flow reactor without external oxidants, base or mediators. The robust electrochemical oxidation is performed for a variety of alcohols with good functional group tolerance, high efficiency and atom economy, whereas mechanistic studies support the benzylic radical intermediate formation and hydrogen evolution. The electrochemical oxidation proves viable on diols with excellent levels of selectivity for the benzylic position.

## Abstract (web summary)

Alcohol oxidation to carbonyl compounds is a very useful functional group transformation in organic synthesis. Here, the authors perform the direct electrochemical oxidation of various alcohols to the corresponding ketones in a continuous-flow reactor without external oxidants, base or mediators.

## Introduction

**Fig. 1:**
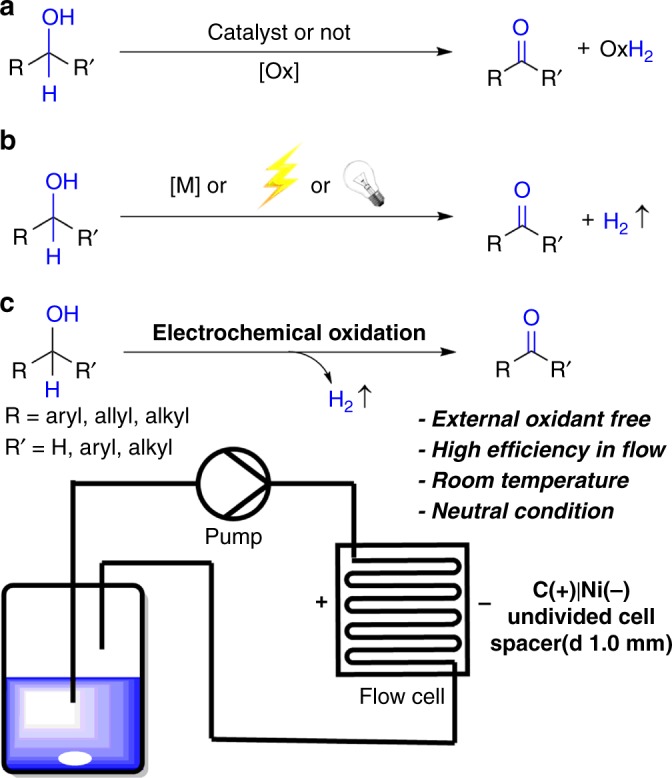
Alcohol oxidation conditions. **a** Alcohols oxidation with oxidant. **b** Alcohols oxidation with dehydrogenation. **c** Alcohol oxidation under continuous-flow set-up

Aldehydes or ketones are not only essential functionalities of various biologically active compounds, but also important reagents in modern organic synthesis^1,2^. In fact, the high frequently used of alcohol oxidations to carbonyl compounds leading to a ranking of 3rd of strongly prefer better reagents for pharmaceutical manufacturers^3^. So, developing oxidation of alcohols under environmentally benign and economic conditions is highly demanded^4–7^ for pharmaceutical and chemical industries. Stoichiometric oxidants such as chromium, manganese, and ruthenium salts^8–15^ are relatively not environmental friendly (Fig. 1a). Molecular oxygen, air, or hydrogen peroxide in combination with appropriate transition metal catalysts (Cu, Ru, Pd, Au, Fe, V, or Ir)^16–22^ represent superior alternatives according to the principles of green and sustainable chemistry. However, the oxidation remains challenging for a broad group of alcohols. In contrast, oxidant-free alcohol oxidation with hydrogen evolution is apparently an ideal process to reach the higher atom economy (Fig. 1b). This concept has been achieved with transition metal catalysis^23–30^, which normally involves expensive transition metals, sophisticated ligands, and generally requires high reaction temperatures. On the other hand, alcohol oxidation with hydrogen release via photocatalysis^31,32^ or electrocatalysis^33–40^ have the merit of running reactions under mild conditions.

Direct electrolysis of alcohols to carbonyl compounds is an idealized goal, which produces only hydrogen without product contamination. The direct electro-oxidation of alcohol can be tracked back to the pioneering work of Lund and Mayeda et al.^41–43^ using undivided electrolysis cells. However, prior attempts gave only the desired aldehydes or ketones in low selectivity and efficiency. We envisioned that electrolysis in a continuous-flow set-up would be a sustainable method to replace stoichiometric oxidants and improve both energy efficiency and productivity. Large surface-to-volume ratio, enhanced mass transfer, operation at quasi-isothermal condition, and lower resistances are the advantages of using flow electrochemistry set-up, which often make it superior than batch mode^44–46^. In addition, the electrode surface can be regenerated efficiently to avoid low conductivity for the transformations with gas evolution. Furthermore, flow cells are easy to scale up and employed in industry^47–49^. Recently, electrochemical flow cells have been successfully used in a variety of organic transformations^50–64^. Herein, we report the direct electrolysis of alcohols to afford the aldehydes or ketones with high efficiency without any mediators or catalysts under neutral conditions by using a flow reactor (Fig. 1c and Supplementary Fig. 1).

## Results

### Investigation of reaction conditions

Our studies commenced with benzyl alcohol (**1aa**), which was subjected to a batch condition in an undivided cell (Table 1, entry 1), yielding 37% of benzaldehyde (**2aa**) at 100 mA constant current electrolysis for 1 h (Supplementary Fig. 2). In order to improve the solubility of the starting material, CH_3_CN and water (1:1) were used as solvents in the continuous-flow set-up, and benzaldehyde (**2aa**) was isolated in 93% yield under the same current (10 mA, 10 h, Table 1, entry 2). Encouraged by these results, we investigated the magnitude of current from 10 mA to 1000 mA (entries 2–8). When 50 mA was applied for the oxidation, 92% of benzaldehyde was isolated in 2 h (Table 1, entry 3). Among a set of studies, 800 mA enabled effective oxidation up to 99% yield in 10 min (Table 1, entry 7). However, when the current was increased to 1000 mA, **2aa** was provided in lower yield (81%, entry 8). The flow rate was another parameter, which was evaluated at the current of 800 mA. Accordingly, 0.10 mL s^−1^ was the optimum flow rate (Table 1, entries 9–11). Then the reaction conditions of 800 mA, 10 min and 0.10 mL s^−1^ were employed for the further researches.

**Table 1:** Optimization of electrochemical oxidation of benzyl alcohol 1aa


Entry	Current	Reaction time	Current density	Flow rate	Yield (%)^a^
1^b^	100 mA	1 h	44.44 mA cm^−2^	Undivided cell	37
2	10 mA	10 h	0.64 mA cm^−2^	0.10 mL s^−1^	93
3	50 mA	2 h	3.19 mA cm^−2^	0.10 mL s^−1^	92
4	100 mA	1 h	6.38 mA cm^−2^	0.10 mL s^−1^	89
5	500 mA	12 min	31.89 mA cm^−2^	0.10 mL s^−1^	90
6	800 mA	8 min	51.02 mA cm^−2^	0.10 mL s^−1^	91
7	1000 mA	6 min	63.78 mA cm^−2^	0.10 mL s^−1^	81
8	800 mA	10 min	51.02 mA cm^−2^	0.10 mL s^−1^	99
9	800 mA	10 min	51.02 mA cm^−2^	0.05 mL s^−1^	67
10	800 mA	10 min	51.02 mA cm^−2^	0.15 mL s^−1^	95
11	800 mA	10 min	51.02 mA cm^−2^	0.20 mL s^−1^	84

Reaction conditions: carbon paper (93 × 93 × 0.2 mm) anode (contact area 1.6 cm^2^), Ni plate (93 × 93 × 0.3 mm) cathode (contact area 1.6 cm^2^), **1aa** (2.0 mmol), ^*n*^Bu_4_NBF_4_ (0.20 mmol), CH_3_CN/H_2_O (1:1, 30 mL), N_2_, room temperature, flow cell (2.49 F mol^−1^)

^a^Isolated yield

^b^Carbon cloth (15  × 15  ×  0.2  mm) anode, Ni plate (15  × 15 × 0.5  ) cathode, **1aa** (2.0  mmol), ^*n*^Bu_4_NBF_4_ (0.20  mmol), CH_3_CN/H_2_O (1:1, 30  mL), N_2_, room temperature, undivided cell (1.86  F  mol^−1^)

**Fig. 2:**
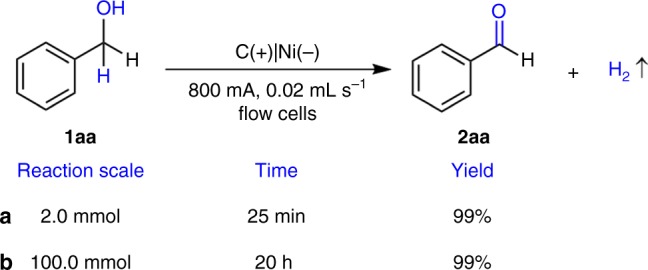
Slow flow rate of electrochemical oxidation of benzyl alcohol **1aa**. **a** The amount of **1aa** is 2.0 mmol. **b** The amount of **1aa** is 100.0 mmol

At the flow rate of 0.10 mL s^−1^, the solution passed through the flow cell multiple times by using peristaltic pump. We think that it would be possible to achieve full conversion by employing large current and small flow rate. By using this strategy, the 2 mmol benzyl alcohol in 30 mL mixed solvent could be quantitatively transformed to benzyaldehyde at 800 mA and 0.02 mL s^−1^ in 25 min (Fig. 2a and Supplementary Fig. 3). Furthermore, the reaction scale can be extended to 100.0 mmol at the same current and the flow rate still in quantitative yield (Fig. 2b and Supplementary Fig. 4). These results illustrated the potential applicability of this method.

### Substrate scope

**Fig. 3:**
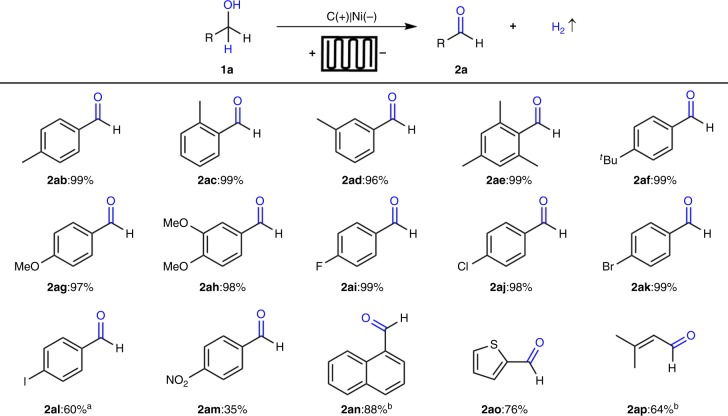
Substrate scopes of electrochemical oxidation of primary alcohols. Reaction conditions: carbon paper (93 × 93 × 0.2 mm) anode (contact area 1.6 cm^2^), Ni plate (93 × 93 × 0.3 mm) cathode (contact area 1.6 cm^2^), constant current = 800 mA, flow rate = 0.10 mL s^−1^, 10 min, **1a** (2.0 mmol), ^*n*^Bu_4_NBF_4_ (0.20 mmol), CH_3_CN/H_2_O (1:1, 30 mL), N_2_, room temperature, flow cell (2.49 F mol^−1^), isolated yield. ^a^10 mA, 10 h. ^b^10 mA, 20 h

With the optimized reaction conditions in hand, we next explored various benzylic and allylic alcohols **1ab-1ap** (2.0 mmol) under galvanostatic conditions (Fig. 3). Electron-rich benzylic alcohols with *ortho-*, *meta-*, and *para*-substitution could be converted to the corresponding aldehydes **2ab-2ah** in nearly quantitative yields (96–99%) without over oxidation. Halogenated benzylic alcohols could be oxidized to afford the corresponding aldehydes **2ai-2ak** in excellent yield (98–99%), which could be further functionalized. Owing to weak C–I bond, 10 mA cell current had to be adapted and 4-iodo benzaldehyde (**2al**) was obtained in moderate yield (60%). Moreover, electron-deficient benzylic alcohols gave less satisfactory results. For example, compound **2am** was obtained in only 35% yield. On the other hand, 1-naphthyl methanol (**1an**) was converted to the corresponding aldehyde **2an** in 88% yield. The flow conditions could also be applied to thiophene derivative **1ao** to afford aldehyde **2ao** in 76% yield without significant side product formation. Additionally, we explored the possibility of electrolysis of allylic alcohol **1ap**, thus obtaining the corresponding unsaturated aldehyde **2ap** in 64% yield.

**Fig. 4:**
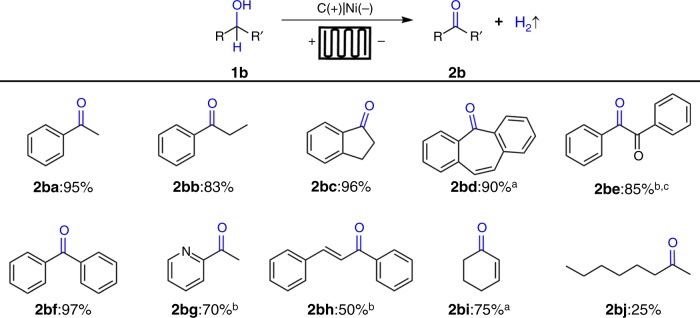
Substrate scopes of electrochemical oxidation of secondary alcohols. Reaction conditions: carbon paper (93 × 93 × 0.2 mm) anode (contact area 1.6 cm^2^), Ni plate (93 × 93 × 0.3 mm) cathode (contact area 1.6 cm^2^), constant current = 800 mA, flow rate = 0.10 mL s^−1^, 10 min, **1b** (2.0 mmol), ^*n*^Bu_4_NBF_4_ (0.20 mmol), CH_3_CN/H_2_O (1:1, 30 mL), N_2_, room temperature, flow cell (2.49 F mol^−1^), isolated yield. ^a^10 mA, 20 h. ^b^10 mA, 10 h. ^c^**1be**: benzoin used as starting material

The reaction conditions were subsequently employed to oxidize a range of secondary alcohols (Fig. 4). Excellent results were observed for oxidation of alcohols **1ba**-**1bf** to the corresponding ketones **2ba-2bf** in 83–97% yield. There was no significant difference in the reactivity of primary and secondary alcohols in the continuous-flow reactor. The oxidation of heterocyclic alcohol **1bg** proceeded smoothly under 10 mA cell current condition to afford ketone **2bg** in 70% yield. Oxidation of allylic alcohols **1bh**−**1bi** afforded the desired ketones in 50–75% yield. However, aliphatic alcohol was not oxidized smoothly under electrochemical oxidation condition and gave **2bj** in 25% yield.

**Fig. 5:**
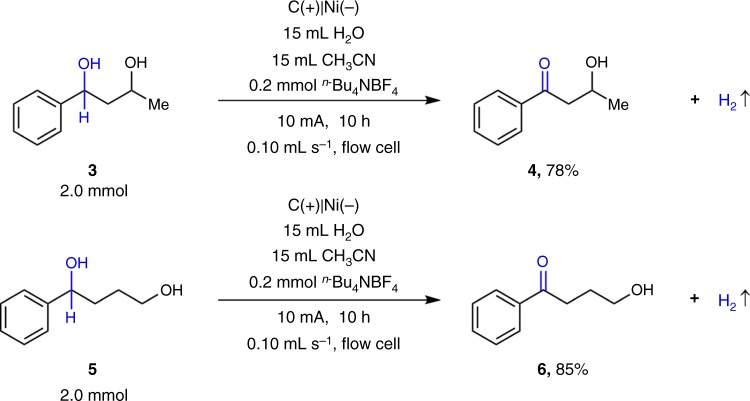
Selective oxidation of benzylic alcohols in the presence of aliphatic alcohols. **a** Selective oxidation of 1-phenylbutane-1,3-diol. **b** Selective oxidation of 1-phenylbutane-1,4-diol

This observation led us to explore the selective oxidation of diols **3** and **5** as shown in Fig. 5. For both substrates, benzylic hydroxyl groups were oxidized selectively in the presence of aliphatic primary or secondary hydroxyl groups to afford hydroxyl ketones **4** and **6** in 78% and 85% yields, respectively. This oxidation could be complementary to Swern oxidation, which is selective for primary or less steric hindered alcohols ^65–67^.

**Fig. 6:**
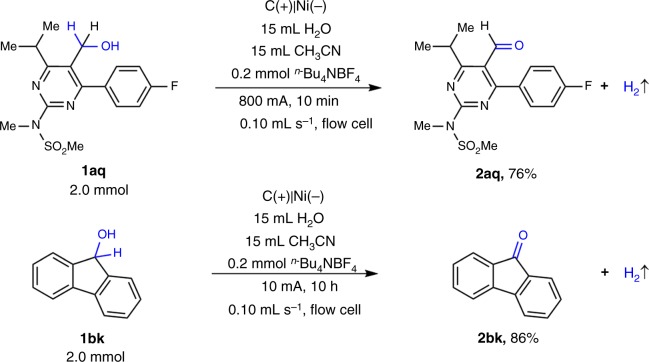
Electrochemical oxidation of biologically relevant substrates. **a** Electrochemical oxidation of Rosuvastatin precursor. **b** Electrochemical oxidation of 9*H*-fluoren-9-ol

The continuous-flow electrolysis was further extended to pharmaceutical relevant substrates (Fig. 6). Rosuvastatin precursor **1aq**^68^ could be oxidized to the corresponding aldehyde in 76% yield within only 10 min, which presented the potential application prospect of this protocol. Fluorenol (**1bk**)^69^ was oxidized to the corresponding ketones in good yields (86%).

**Fig. 7:**
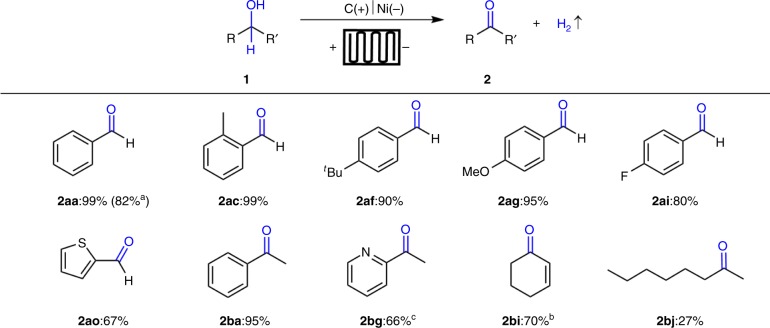
Substrate scopes of electrochemical oxidation of alcohols in water. Reaction conditions: carbon paper (93 × 93 × 0.2 mm) anode (contact area 1.6 cm^2^), Ni plate (93 × 93 × 0.3 mm) cathode (contact area 1.6 cm^2^), constant current = 800 mA, flow rate = 0.10 mL s^−1^, 10 min, **1** (2.0 mmol), *N,N,N*-trimethylhexadecan-1-ammonium sulfate (0.10 mmol), H_2_O (15 mL), N_2_, room temperature, flow cell (2.49 F mol^−1^), isolated yield. ^a^LiClO_4_ instead of *N,N,N*-trimethylhexadecan-1-ammonium sulfate. ^b^10 mA, 20 h. ^c^10 mA, 10 h

The possibility of using water as solvent was also estimated. However, benzaldehyde was obtained in only 82% yield due to the poor solubility of alcohols in water. Thus, the surfactant was employed and we noted that the ionic surfactant could also be the supporting electrolyte. By using this strategy, benzaldehyde could be prepared in quantitative yield in water (Fig. 7). Six primary alcohols in Fig. 3 and four secondary alcohols in Fig. 4 have been chosen to re-evaluate the yields in the presence of surfactant in pure water. In general, aqueous conditions provided the desired products in comparable yields with our standard conditions, which showed that the combination of water and surfactant would be a good choice for this protocol.

**Fig. 8:**
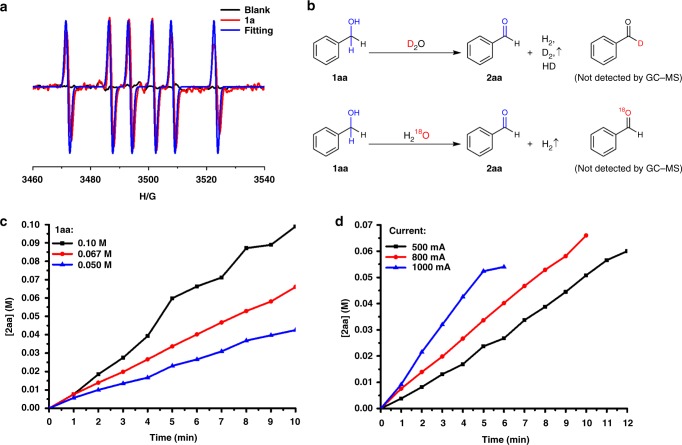
Mechanistic studies experiments. **a** EPR results of benzyl alcohol oxidation. **b** D_2_O and H_2_^18^O labeling experiments. **c** Kinetic profiles under different concentrations of **1aa**. **d** Kinetic profiles under different current

Since the oxidation of alcohol in the anode means the loss of electron, the existence of radical intermediate is highly probable. To gain insight into the reaction mechanism, electron paramagnetic resonance (EPR) experiments were performed by adding the radical spin trapping agent DMPO (5, 5-dimethyl-1-pyrroline N-oxide). No radical signal was detected in the absence of **1aa** (Fig. 8a, blank line). When DMPO was added to the reaction under constant current conditions, a radical signal (*g* = 2.0069, *A*_N_ = 14.82, *A*_H_ = 21.42) was identified (Fig. 8a, red line). According to the fitting result, this radical signal came from benzyl radical captured by DMPO.

No deuterium or ^18^O was incorporated into benzaldehyde when H_2_O was substituted with D_2_O or H_2_^18^O in the reaction system (Fig. 8b and Supplementary Figs. 5 and 6). This result indicated that water did not react with the intermediate under the reaction conditions. This was in accordance with no over oxidation to benzoic acid was observed even when benzaldehyde was used as starting material for this electrolysis (Supplementary Fig. 8). In addition, no over oxidation of the aldehyde products may also benefit from the fact that no extra base was added in our reaction system.

The influence of the concentration, current, and flow rate to the electrochemical oxidation of benzyl alcohol have been evaluated as shown in Fig. 8. Reaction rate increased with the increasing of the concentration of benzyl alcohol (Fig. 8c). It was the same that large current meant high reaction rate (Fig. 8d). The reaction rate kept unchanged when the flow rate was larger than 0.10 mL s^−1^ (Supplementary Fig. 7). These results suggested that the electrochemical oxidation was likely to be the rate-limiting step during electrolysis.

**Fig. 9:**
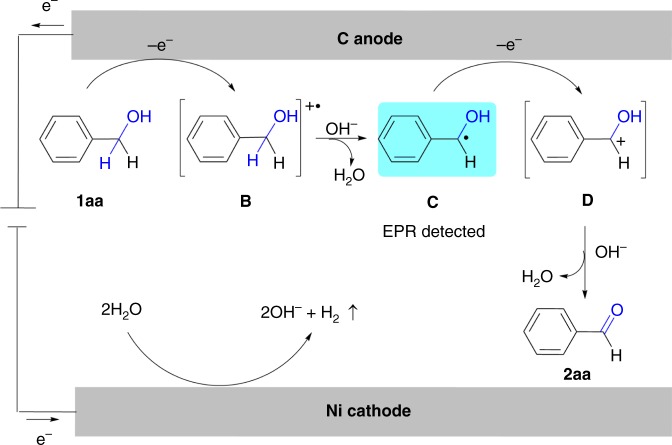
Proposed mechanism of benzyl alcohol electrochemical oxidation. A tentative reaction mechanism involves anodic oxidation of benzyl alcohol to afford intermediate **B**, deprotonation, single electron oxidation and deprotonation to form the desired benzaldehyde (**2aa**). Water underwent cathodic reduction to generate hydroxide accompanied by releasing hydrogen

On the basis of our mechanistic studies and literature reports^41–43^, a possible mechanism for the oxidation of alcohols to carbonyl functionality is depicted in Fig. 9. The oxidation of benzyl alcohol was initiated by anodic oxidation to afford intermediate **B**. The consequent deprotonation of the radical cation **B** resulted in the formation of benzylic radical **C**, which has been detected by EPR. The following fast single-electron oxidation and deprotonation of **D** produce the desired benzaldehyde (**2aa**). Lower efficiency of oxidation with electron deficient benzylic alcohols could be explained by considering that electron withdrawing groups destabilized intermediates. In the meantime, water underwent cathodic reduction to generate hydroxide accompanied by releasing hydrogen^70^. The in situ formed hydroxide acted as base to trap the protons. For the whole reaction, no external oxidant was needed, which is in accordance with the idea of green chemistry.

In summary, a direct electrochemical oxidation of alcohols to the corresponding carbonyl compounds has been accomplished efficiently by the continuous-flow reactor just using carbon anode. Reactions were performed without external oxidant, mediator or additive and no over oxidation was observed, which make this method an ideal transformation from alcohols to aldehydes or ketones. Even water can be used as solvent in the presence of surfactant. The reaction conditions have been applied for selective oxidation and biologically relevant substrates. The reaction can be adjusted conveniently from milligram to gram scale based on demand by using the flow set-up. Further research to broaden the substrate scope of alcohol oxidation will be reported in due course.

## Methods

### General procedures for the electrolysis in acetonitrile and water

In an oven-dried schlenck tube (100 mL) equipped with a stir bar, alcohol **1a** (2.0 mmol), ^*n*^Bu_4_NBF_4_ (65.9 mg, 0.2 mmol) and CH_3_CN/H_2_O (1:1, 30 mL) were added. The flow cell was equipped with carbon paper (9.3 cm × 9.3 cm × 0.2 mm) as the anode (contact area 1.6 cm^2^) and nickel plate (9.3 cm × 9.3 cm × 0.3 mm) as the cathode (contact area 1.6 cm^2^). In order to preclude the possibility that air was involved in the oxidation of alcohol, we flushed the whole system with nitrogen before the direct electrolysis. The reaction mixture was pumped into the electrochemical reactor at the flow rate of 0.10 mL s^−1^ (Supplementary Fig. 1). Method A: A constant current of 800 mA was employed during the electrolysis under room temperature for 10 min. (Method B: A constant current of 10 mA was employed during the electrolysis under room temperature for 10 h. Method C: A constant current of 10 mA was employed during the electrolysis under room temperature for 20 h.) When the reaction was finished, the reaction mixture was washed with water and extracted with dichloromethane (10 mL x 3). The organic layers were combined, dried over Na_2_SO_4_, and concentrated. The pure product was obtained by flash column chromatography on silica gel using petroleum ether and ethyl acetate as the eluent.

### General procedures for the electrolysis in water with surfactant

Method D: In an oven-dried schlenck tube (100 mL) equipped with a stir bar, alcohol **1a** (2.0 mmol), *N,N,N*-trimethylhexadecan-1-ammonium sulfate (69.6 mg, 0.10 mmol) and H_2_O (15 mL) were added. The flow cell was equipped with carbon paper (9.3 cm × 9.3 cm × 0.2 mm) as the anode (contact area 1.6 cm^2^) and nickel plate (9.3 cm × 9.3 cm × 0.3 mm) as the cathode (contact area 1.6 cm^2^). In order to preclude the possibility that air was involved in the oxidation of alcohol, we flushed the whole system with nitrogen before the direct electrolysis. The reaction mixture was pumped into the electrochemical reactor in a flow rate of 0.10 mL s^−1^ (Supplementary Fig. 1). A constant current of 800 mA was employed during the electrolysis under room temperature for 10 min. When the reaction was finished, the reaction mixture was washed with water and extracted with dichloromethane (10 mL x 3). The organic layers were combined, dried over Na_2_SO_4_, and concentrated. The pure product was obtained by flash column chromatography on silica gel using petroleum ether and ethyl acetate as the eluent.

## Supplementary information

Supplementary Information

## Data Availability

The authors declare that the data supporting the findings of this study are available within the article and its Supplementary Information files.
