# Supplementary material for: Direct electrochemical oxidation of alcohols with hydrogen evolution in continuous-flow reactor
Source: Nat Commun. 2019 Jun 26;10:2796. doi: 10.1038/s41467-019-10928-0 (PMC6594969; doi:10.1038/s41467-019-10928-0)
Supplement: Supplementary file 1 — Supplementary Information [file 41467_2019_10928_MOESM1_ESM.pdf]

Direct Electrochemical Oxidation of Alcohols with Hydrogen Evolution in Continuous-flow Reactor

Wang et al.

Supplementary Methods

All glassware was oven dried at 110 °C for hours and cooled down under vacuum. Unless otherwise noted, materials were obtained from commercial suppliers and used without further purification. Switching DC voltage regulator (HSPY-120-01) (made in China). Cyclic voltammograms were obtained on a CHI 605E potentiostat. The anodic electrode was carbon paper (9.3 cm×9.3 cm×0.2 mm) and cathodic electrode was nickel plate (9.3 cm×9.3 cm×0.3 mm). Thin layer chromatography (TLC) employed glass 0.25 mm silica gel plates. Flash chromatography columns were packed with 200-300 mesh silica gel in petroleum (bp. 60-90 °C). EPR spectra were recorded on a Bruker X-band A-200 spectrometer. Mass spectra were recorded using a SATURN-2100 GC-MS spectrometer. Hydrogen gas content was analyzed by gas chromatography (QIC-20, Hiden, argon as a carrier gas and 5 Å molecular sieve column, a thermal conductivity detector). ^{1}H and ^{13}C NMR data were recorded with Bruker Advance III (400 MHz) spectrometers with tetramethylsilane as an internal standard. All chemical shifts (δ) are reported in ppm and coupling constants (*J*) in Hz. All chemical shifts were reported relative to tetramethylsilane (0 ppm for ^{1}H), $CDCl_3$ (77.0 ppm for ^{13}C), respectively.

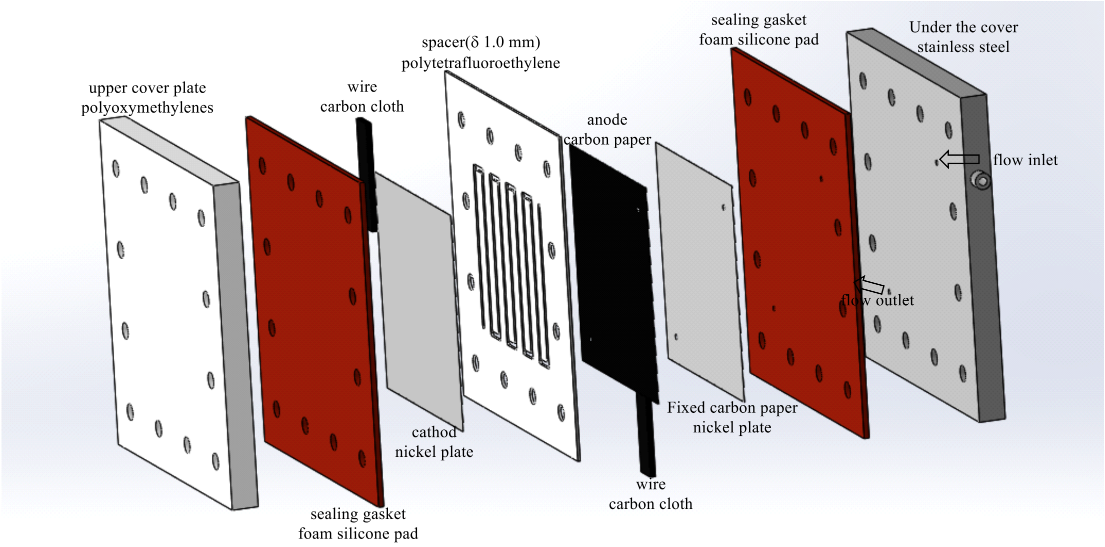

a: Physical map of the device

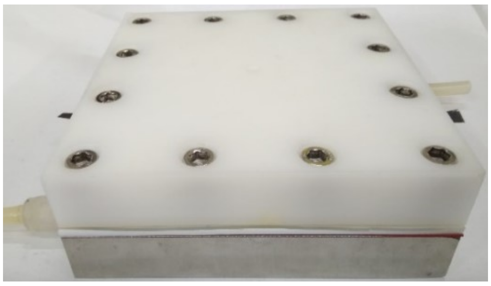

b: Flow cell

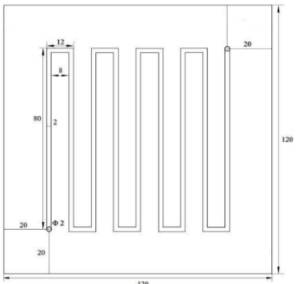

c: Polytetrafluoroethylene (PTEF) board

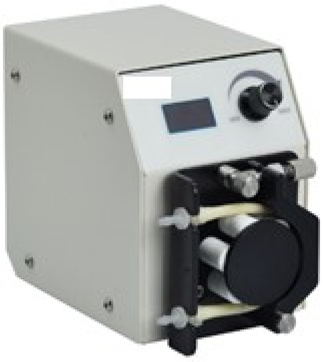

d: Peristaltic pump

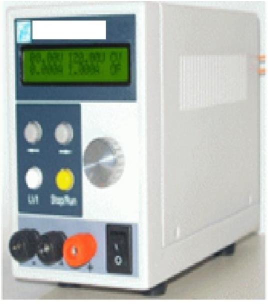

e: Switching DC voltage regulator

Supplementary Figure 1. The components of the electrochemical continuous-flow reactor. (a) Physical map of the device. (b) Flow cell. (c) Polytetrafluoroethylene (PTEF) board. (d) Peristaltic pump. (e) Switching DC voltage regulator.

Parameters of the flow reactor: The anode and cathode are held apart by a polytetrafluoroethylene (PTEF) board of 1.0 mm thick. A rectangular reaction channel (total length: 784 mm, width: 1.0 mm) is cut in the PTEF foil to give an overall channel volume of 1.6 mL. The whole device is held together by steel screws. The pump used in the experiment is a KCS PRO model peristaltic pump manufactured by Kamoer. Switching DC voltage regulator (HSPY-120-01) (made in China). (See Supplementary Figure 1)

General procedures for the electrolysis in acetonitrile and water: In an oven-dried schlenck tube (100 mL) equipped with a stir bar, alcohol **1a** (2.0 mmol), $^{n}Bu_4NBF_4$ (65.9 mg, 0.2 mmol) and CH_3CN/H_2O (1:1, 30 mL) were added. The flow cell was equipped with carbon paper (9.3 cm×9.3 cm×0.2 mm) as the anode (contact area 1.6 cm^2) and nickel plate (9.3 cm×9.3 cm×0.3 mm) as the cathode (contact area 1.6 cm^2). In order to preclude the possibility that air was involved in the oxidation of alcohol, we flushed the whole system with nitrogen before the direct electrolysis. The reaction mixture was pumped into the electrochemical reactor at the flow rate of 0.10 mL s^{-1}

(Supplementary Figure 1). **Method A**: A constant current of 800 mA was employed during the electrolysis under room temperature for 10 min. (**Method B**: A constant current of 10 mA was employed during the electrolysis under room temperature for 10 h. **Method C**: A constant current of 10 mA was employed during the electrolysis under room temperature for 20 h.) When the reaction was finished, the reaction mixture was washed with water and extracted with dichloromethane (10 mL x 3). The organic layers were combined, dried over Na_2SO_4, and concentrated. The pure product was obtained by flash column chromatography on silica gel using petroleum ether and ethyl acetate as the eluent.

General procedures for the electrolysis in water with surfactant (Method D): In an oven-dried schlenck tube (100 mL) equipped with a stir bar, alcohol **1a** (2.0 mmol), *N,N,N*-trimethylhexadecan-1-ammonium sulfate (69.6 mg, 0.10 mmol) and H_2O (15 mL) were added . The flow cell was equipped with carbon paper (9.3 cm×9.3 cm×0.2 mm) as the anode (contact area 1.6 cm^2) and nickel plate (9.3 cm×9.3 cm×0.3 mm) as the cathode (contact area 1.6 cm^2). In order to preclude the possibility that air was involved in the oxidation of alcohol, we flushed the whole system with nitrogen before the direct electrolysis. The reaction mixture was pumped into the electrochemical reactor in a flow rate of 0.10 mL s^{-1} (Supplementary Figure 1). A constant current of 800 mA was employed during the electrolysis under room temperature for 10 min. When the reaction was finished, the reaction mixture was washed with water and extracted with dichloromethane (10 mL x 3). The organic layers were combined, dried over Na_2SO_4, and concentrated. The pure product was obtained by flash column chromatography on silica gel using petroleum ether and ethyl acetate as the eluent.

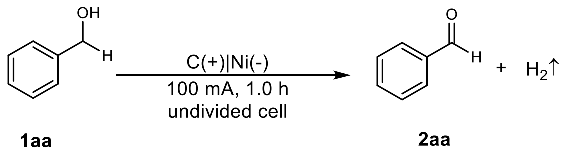

Supplementary Figure 2. Oxidation of benzyl alcohol in undivided cell.

Procedures for the electrolysis in undivided cell: In an oven-dried undivided three-necked flask (50 mL) equipped with a stir bar, the benzyl alcohol **1aa** (216.1 mg, 2.0 mmol) and $^{n}Bu_4NBF_4$ (65.9 mg, 0.2 mmol) was dissolved in CH_3CN/H_2O (1:1, 30 mL). The flask was equipped with carbon cloth (1.5 cm×1.5 cm×0.3 mm) as the anode and nickel plate (1.5 cm×1.5 cm×0.3 mm) as

the cathode. In order to preclude the possibility that air was involved in the oxidation of alcohol, we flushed the whole system with nitrogen before the direct electrolysis. The reaction mixture was stirred and electrolyzed at a constant current of 100 mA under room temperature for 1 h. When the reaction was finished, the reaction mixture was washed with water and extracted with dichloromethane (10 mL x 3). The organic layers were combined, dried over Na_2SO_4, and concentrated. The pure product was obtained by flash column chromatography on silica gel using petroleum ether and ethyl acetate as the eluent. Yield: 37%. (See Supplementary Figure 2)

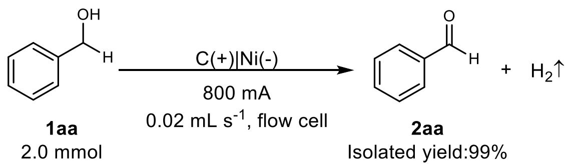

Supplementary Figure 3. Oxidation of benzyl alcohol at slow flow rate in flow cell.

Procedures for slow flow rate of alcohol oxidation in flow cell: In an oven-dried schlenck tube (100 mL) equipped with a stir bar, benzyl alcohol **1aa** (216.1 mg, 2.0 mmol), ^{n}Bu$_4$NBF$_4$ (65.9 mg, 0.2 mmol) and CH_3CN/H_2O (1:1, 30 mL) were added. The flow cell was equipped with carbon paper (9.3 cm×9.3 cm×0.2 mm) as the anode (contact area 1.6 cm^2) and nickel plate (9.3 cm×9.3 cm×0.3 mm) as the cathode (contact area 1.6 cm^2). In order to preclude the possibility that air was involved in the oxidation of alcohol, we flushed the whole system with nitrogen before the direct electrolysis. The reaction mixture was pumped into the electrochemical reactor for single-pass in a flow rate of 0.02 mL s^{-1}. A constant current of 800 mA was employed during the electrolysis under room temperature for 25 min. When the reaction was finished, the reaction mixture was washed with water and extracted with dichloromethane (10 mL x 3). The organic layers were combined, dried over Na_2SO_4, and concentrated. The pure product was obtained by flash column chromatography on silica gel using petroleum ether and ethyl acetate as the eluent. Yield: 99%. (See Supplementary Figure 3)

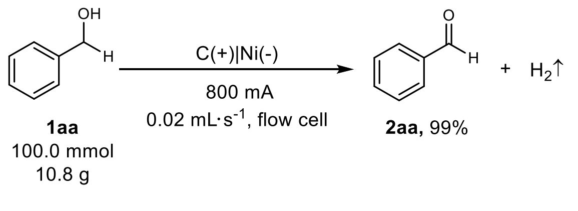

Supplementary Figure 4. Gram scale synthesis of benzyl alcohol oxidation in flow cell.

Procedures for gram scale synthesis in flow cell: In an oven-dried schlenck tube (1000 mL) equipped with a stir bar, benzyl alcohol benzyl alcohol **1aa** (10.8 g, 100.0 mmol), $^{n}Bu_4NBF_4$ (329.3 mg, 1.0 mmol) and CH_3CN/H_2O (1:1, 1000 mL) were added. The flow cell was equipped with carbon paper (9.3 cm×9.3 cm×0.2 mm) as the anode (contact area 1.6 cm^2) and nickel plate (9.3 cm×9.3 cm×0.3 mm) as the cathode (contact area 1.6 cm^2). In order to preclude the possibility that air was involved in the oxidation of alcohol, we flushed the whole system with nitrogen before the direct electrolysis. The reaction mixture was pumped into the electrochemical reactor for single-pass in a flow rate of 0.02 mL s^{-1}. A constant current of 800 mA was employed during the electrolysis under room temperature for 20.0 h. When the reaction was finished, the reaction mixture was washed with water and extracted with dichloromethane (200 mL x 3). The organic layers were combined, dried over Na_2SO_4, and concentrated. The pure product was obtained by flash column chromatography on silica gel using petroleum ether and ethyl acetate as the eluent. Isolated yield: 99%, 10.6 g. (See Supplementary Figure 4)

Procedures for the electron paramagnetic resonance (EPR) experiment (EPR studies of 1a): Under 10 mA constant current conditions, a dried three-necked flask equipped with a stir bar was loaded with **1aa** (54.0 mg, 0.50 mmol), $^{n}Bu_4NBF_4$ (65.9 mg, 0.2 mmol) in CH_3CN (10.0 mL) was stirred under an N_2 atmosphere at 25 ºC. The flask was equipped with carbon cloth (1.5 cm×1.5 cm×0.3 mm) as the anode and nickel plate (1.5 cm×1.5 cm×0.3 mm) as the cathode. In order to preclude the possibility that air was involved in the oxidation of alcohol, we flushed the whole system with nitrogen before the direct electrolysis. The reaction mixture was stirred and electrolyzed at a constant current of 10 mA under room temperature for 1 h. DMPO was added to the reaction mixture and continued to react for 1 minute; the solution sample was taken out into a small tube for EPR test. EPR spectra was recorded at room temperature on EPR spectrometer operated at 9.823 GHz. Typical spectrometer parameters were shown as follows, sweep width: 100 G; center field set: 3505 G; time constant: 163.84 ms; sweep time: 30.72 s; modulation amplitude: 1.0 G; modulation frequency: 100 kHz; receiver gain: 1.00×10^4; microwave power: 22.10 mW.

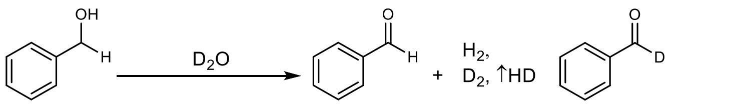

Supplementary Figure 5. Isotope labeling experiment of D_2O.

Procedures for isotope labeling experiments (D_2O): In an oven-dried schlenck tube (100 mL) equipped with a stir bar, benzyl alcohol **1aa** (216.1 mg, 2.0 mmol), $^{n}Bu_4NBF_4$ (65.9 mg, 0.2 mmol) and CH_3CN/ D_2O (1:1, 30 mL) were added. The flow cell was equipped with carbon paper (9.3 cm×9.3 cm×0.2 mm) as the anode (contact area 1.6 cm^2) and nickel plate (9.3 cm×9.3 cm×0.3 mm) as the cathode (contact area 1.6 cm^2). In order to preclude the possibility that air was involved in the oxidation of alcohol, we flushed the whole system with nitrogen before the direct electrolysis. The reaction mixture was pumped into the electrochemical reactor for single-pass in a flow rate of 0.10 mL s^{-1}. A constant current of 800 mA was employed during the electrolysis under room temperature for 10 min. When the reaction was finished, the reaction mixture was detected by GC-MS, but the target product marked by isotope was not detected, and H_2, D_2, HD was detected. (See Supplementary Figure 5)

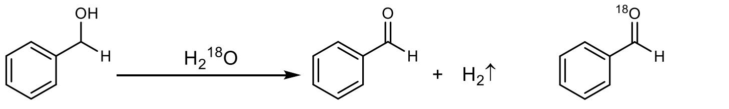

Supplementary Figure 6. Isotope labeling experiment of $H_2{}^{18}O$.

Procedures for isotope labeling experiments ($H_2{}^{18}O$): In an oven-dried schlenck tube (100 mL) equipped with a stir bar, benzyl alcohol 1aa (216.1 mg, 2.0 mmol), $^{n}Bu_4NBF_4$ (65.9 mg, 0.2 mmol) $H_2{}^{18}O$(40.0 mg, 2.0 mmol) and CH_3CN (30 mL) were added. The flow cell was equipped with carbon paper (9.3 cm×9.3 cm×0.2 mm) as the anode (contact area 1.6 cm^2) and nickel plate (9.3 cm×9.3 cm×0.3 mm) as the cathode (contact area 1.6 cm^2). In order to preclude the possibility that air was involved in the oxidation of alcohol, we flushed the whole system with nitrogen before the direct electrolysis. The reaction mixture was pumped into the electrochemical reactor for single-pass in a flow rate of 0.10 mL s^{-1}. A constant current of 800 mA was employed during the electrolysis under room temperature for 10 min. When the reaction was finished, the reaction mixture was the

reaction mixture was detected by GC-MS, but the target product marked by isotope was not detected. (See Supplementary Figure 6)

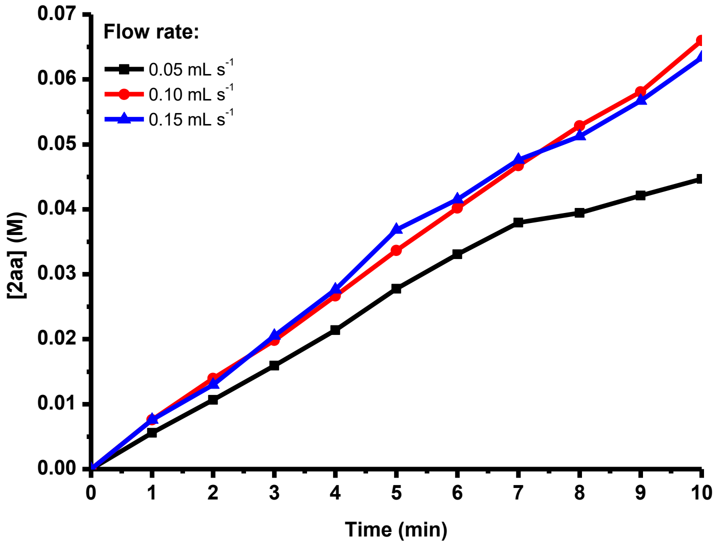

Supplementary Figure 7. Kinetic profiles under different flow rates of **1aa**.

General procedure for kinetic study (Flow rate): In an oven-dried schlenck tube (100 mL) equipped with a stir bar, alcohol **1aa** (2.0 mmol), $^{n}Bu_4NBF_4$ (65.9 mg, 0.2 mmol) and CH_3CN/H_2O (1:1, 30 mL) were added. The flow cell was equipped with carbon paper (9.3 cm×9.3 cm×0.2 mm) as the anode (contact area 1.6 cm^2) and nickel plate (9.3 cm×9.3 cm×0.3 mm) as the cathode (contact area 1.6 cm^2). In order to preclude the possibility that air was involved in the oxidation of alcohol, we flushed the whole system with nitrogen before the direct electrolysis. The reaction mixture was pumped into the electrochemical reactor in a flow rate of 0.10 mL s^{-1}. A constant current of 800 mA was employed during the electrolysis under room temperature. 0.10 mL solution were taken out from the schlenck tube via syringe per minute. By using above procedure, the similar sets of experiments were conducted by using different concentration of **1aa**. Moreover, the similar sets of experiments were also conducted under different constant current and flow rate. Only one parameter was changed from the general procedure in one reaction. (See Supplementary Figure 7)

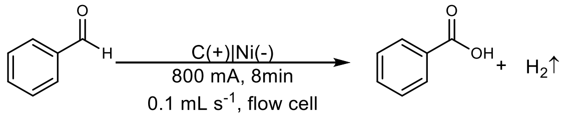

2aa **3aa**

Supplementary Figure 8. Oxidation of benzaldehyde in a flow cell.

Procedures for conversion of benzaldehyde oxidation in flow cell: In an oven-dried schlenck tube (100 mL) equipped with a stir bar, benzaldehyde **2aa** (212.1 mg, 2.0 mmol), $^{n}Bu_4NBF_4$ (65.9 mg, 0.2 mmol) and CH_3CN/H_2O (1:1, 30 mL) were added. The flow cell was equipped with carbon paper (9.3 cm×9.3 cm×0.2 mm) as the anode (contact area 1.6 cm^2) and nickel plate (9.3 cm×9.3 cm×0.3 mm) as the cathode (contact area 1.6 cm^2). In order to preclude the possibility that air was involved in the oxidation of alcohol, we flushed the whole system with nitrogen before the direct electrolysis. The reaction mixture was flowed through the electrochemical reactor in a flow rate of 0.10 mL s^{-1}. A constant current of 800 mA was employed during the electrolysis under room temperature for 8 min. When the reaction was finished, the reaction mixture was washed with water and extracted with dichloromethane (10 mL x 3). The organic layers were combined, dried over Na_2SO_4, and concentrated. The pure product was obtained by flash column chromatography on silica gel using petroleum ether and ethyl acetate as the eluent. No benzoic acid was detected. (See Supplementary Figure 8)

Detail Descriptions for Products

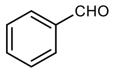

Benzaldehyde (2aa):[6] colorless liquid was obtained in 98% isolated yield (product weight: 207.0 mg) following method A. ^{1}H NMR (400 MHz, $CDCl_3$) δ 10.00 (s, 1H), 7.90 – 7.84 (m, 2H), 7.64 – 7.58 (m, 1H), 7.55 – 7.48 (m, 2H). ^{13}C NMR (101 MHz, $CDCl_3$) δ 192.19, 136.15, 134.26, 129.51, 128.79.

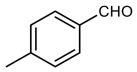

4-Methylbenzaldehyde (2ab):[6] colorless liquid was obtained in 99% isolated yield (product weight: 237.6 mg) following method A. ^{1}H NMR (400 MHz, $CDCl_3$) δ 9.93 (s, 1H), 7.74 (d, J = 8.1 Hz, 2H), 7.29 (d, J = 7.9 Hz, 2H), 2.39 (s, 3H). ^{13}C NMR (101 MHz, $CDCl_3$) δ 191.69, 145.26, 133.90, 129.55, 129.44, 21.57.

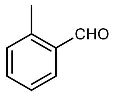

2-Methylbenzaldehyde (2ac):[2] colorless liquid was obtained in 99% isolated yield (product weight: 237.6 mg) following method A. ^{1}H NMR (400 MHz, $CDCl_3$) δ 10.21 (s, 1H), 7.76-7.74 (m, 1H), 7.46-7.41 (m, 1H), 7.34-7.30 (m, 1H), 7.22-7.20 (m, 1H), 2.62 (s, 3H).. ^{13}C NMR (101 MHz, $CDCl_3$) δ 192.76, 140.51, 134.00, 133.56, 131.95, 131.66, 126.22, 19.49.

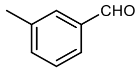

3-Methylbenzaldehyde (2ad):[1] colorless liquid was obtained in 96% isolated yield (product weight: 237.6 mg) following method A. ^{1}H NMR (400 MHz, $CDCl_3$) δ 9.98 (s, 1H), 7.68-7.66 (m, 2H), 7.45 – 7.40 (m, 2H), 2.42 (s, 3H). ^{13}C NMR (101 MHz, $CDCl_3$) δ 192.53, 138.80, 136.32, 135.20, 129.91, 128.77, 127.12, 21.09.

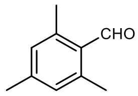

2,4,6-Trimethylbenzaldehyde (2ae):[6] colorless liquid was obtained in 99% isolated yield (product weight: 293.0 mg) following method A. ^{1}H NMR (400 MHz, $CDCl_3$) δ 10.53 (s, 1H), 6.87 (s, 2H), 2.56 (s, 6H), 2.30 (s, 3H). ^{13}C NMR (101 MHz, $CDCl_3$) δ 192.81, 143.69, 141.33, 130.37, 129.73, 21.31, 20.36.

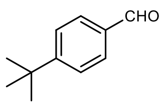

4-(T*ert*-butyl)benzaldehyde (2af):[1] pale yellow liquid was obtained in 99% isolated yield (product weight: 320.8 mg) following method A. ^{1}H NMR (400 MHz, $CDCl_3$) δ 9.97 (s, 1H), 7.84 – 7.80 (m, 2H), 7.57 – 7.53 (m, 2H), 1.35 (s, 9H). ^{13}C NMR (101 MHz, $CDCl_3$) δ 191.94, 158.29, 133.91, 129.58, 125.86, 35.21, 30.93.

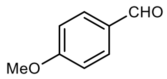

4-Methoxybenzaldehyde (2ag):[6] colorless liquid was obtained in 97% isolated yield (product weight: 263.8 mg) following method A. ^{1}H NMR (400 MHz, $CDCl_3$) δ 9.87 (s, 1H), 7.85 – 7.80 (m, 2H), 7.02 – 6.96 (m, 2H), 3.86 (s, 3H). ^{13}C NMR (101 MHz, $CDCl_3$) δ 190.66, 164.36, 131.74, 129.61, 114.06, 55.32.

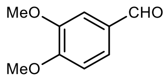

3,4-Dimethoxybenzaldehyde (2ah):[3] white solid was obtained in 98% isolated yield (product weight: 325.4 mg) following method A. ^{1}H NMR (400 MHz, $CDCl_3$) δ 9.73 (s, 1H), 7.35 (dd, J = 8.2, 1.9 Hz, 1H), 7.29 (d, J = 1.8 Hz, 1H), 6.87 (d, J = 8.2 Hz, 1H), 3.85 (s, 3H), 3.82 (s, 3H). ^{13}C NMR (101 MHz, $CDCl_3$) δ 190.58 , 154.07, 149.17, 129.69, 126.53, 110.04, 108.48, 55.80, 55.58.

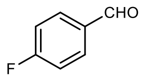

4-Fluorobenzaldehyde (2ai):[1] colorless liquid was obtained in 99% isolated yield (product weight: 245.5 mg) following method A. ^{1}H NMR (400 MHz, $CDCl_3$) δ 9.97 (s, 1H), 7.97 – 7.88 (m, 2H), 7.28 – 7.18 (m, 2H). ^{13}C NMR (101 MHz, $CDCl_3$) δ 190.44, 166.37(d, $J_{C\text{-}F}$ = 255.0 Hz), 132.83 (d, $J_{C\text{-}F}$ = 2.7 Hz), 132.12 (d, $J_{C\text{-}F}$ = 9.8 Hz), 116.22(d, $J_{C\text{-}F}$ = 22.2 Hz). ^{19}F NMR (377 MHz, $CDCl_3$) δ -102.44.

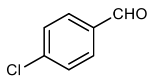

4-Chlorobenzaldehyde (2aj):[6] white solid was obtained in 98% isolated yield (product weight: 274.4 mg) following method A. ^{1}H NMR (400 MHz, $CDCl_3$) δ 9.99 (s, 1H), 7.85 – 7.81 (m, 2H), 7.54 – 7.50 (m, 2H). ^{13}C NMR (101 MHz, $CDCl_3$) δ 190.85, 140.87, 134.60, 130.85, 129.38.

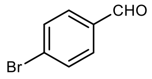

4-Bromobenzaldehyde (2ak):[6] white solid was obtained in 99% isolated yield (product weight:

364.3 mg) following method A. ^{1}H NMR (400 MHz, $CDCl_3$) δ 9.98 (s, 1H), 7.79 – 7.73 (m, 2H), 7.72 – 7.66 (m, 2H). ^{13}C NMR (101 MHz, $CDCl_3$) δ 191.08, 134.99, 132.40, 130.94, 129.76.

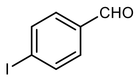

4-Bromobenzaldehyde (2ak):[1] white solid was obtained in 60% isolated yield (product weight: 278.4 mg) following method B. ^{1}H NMR (400 MHz, $CDCl_3$) δ 9.96 (s, 1H), 7.93-7.91 (m, 2H), 7.62 – 7.57 (m, 2H). ^{13}C NMR (101 MHz, $CDCl_3$) δ 191.44, 138.38, 135.50, 130.78, 102.84.

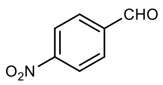

4-Nitrobenzaldehyde (2am):[6] pale yellow solid was obtained in 35% isolated yield (product weight: 105.7 mg) following method A. ^{1}H NMR (400 MHz, $CDCl_3$) δ 10.18 (s, 1H), 8.43 – 8.39 (m, 2H), 8.13 – 8.08 (m, 2H). ^{13}C NMR (101 MHz, $CDCl_3$) δ 190.31, 151.04, 139.97, 130.46, 124.27.

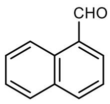

1-Naphthaldehyde (2an):[1] pale yellow solid was obtained in 88% isolated yield (product weight: 274.6 mg) following method C. ^{1}H NMR (400 MHz, $CDCl_3$) δ 10.14 (s, 1H), 9.18-9.16 (m, 1H), 7.76-7.74 (m, 1H), 7.65 – 7.60 (m, 2H), 7.51-7.47 (m, 1H), 7.39 – 7.35 (m, 1H), 7.29-7.25 (m, 1H). ^{13}C NMR (101 MHz, $CDCl_3$) δ 193.37, 136.66, 135.07, 133.51, 131.09, 130.25, 128.90, 128.46, 126.81, 124.78.

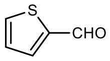

Thiophene-2-carbaldehyde (2ao):[1] pale yellow liquid was obtained in 76% isolated yield (product weight: 170.2 mg) following method A. ^{1}H NMR (400 MHz, $CDCl_3$) δ 9.94 (s, 1H), 7.82 – 7.76 (m, 2H), 7.24-7-22 (m, 1H). ^{13}C NMR (101 MHz, $CDCl_3$) δ 182.98, 143.85, 136.36, 135.08, 128.25.

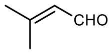

3-Methylbut-2-enal:[8] pale yellow liquid was obtained in 64% isolated yield (product weight: 107.5 mg) following method C. ^{1}H NMR (400 MHz, $CDCl_3$) δ 9.96 (d, J = 8.2 Hz, 1H), 5.91-5.87 (m, 1H), 2.18 (s, 3H), 1.99 (s, 3H). ^{13}C NMR (101 MHz, $CDCl_3$) δ 190.98, 160.69, 127.93, 27.11, 18.76.

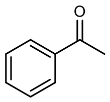

Acetophenone (2ba):[1] pale yellow liquid was obtained in 95% isolated yield (product weight: 228.0 mg) following method A. ^{1}H NMR (400 MHz, $CDCl_3$) δ 7.98 – 7.91 (m, 2H), 7.58 – 7.51 (m, 1H),

7.48 – 7.40 (m, 2H), 2.61 (s, 3H). ^{13}C NMR (101 MHz, $CDCl_3$) δ 197.90, 136.82, 132.89, 128.34, 128.06, 26.39.

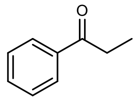

Propiophenone (2bb):[4] pale yellow liquid was obtained in 83% isolated yield (product weight: 222.4 mg) following method A. ^{1}H NMR (400 MHz, $CDCl_3$) δ 7.97-7.95 (m, 2H), 7.57-7.53 (m, 1H), 7.48 – 7.42 (m, 2H), 3.00 (q, J = 7.2 Hz, 2H), 1.22 (t, J = 7.2 Hz, 3H). ^{13}C NMR (101 MHz, $CDCl_3$) δ 200.74, 136.80, 132.80, 128.46, 127.88, 31.69, 8.15.

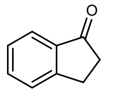

2,3-Dihydro-*1H*-inden-1-one (2bc):[4] white solid was obtained in 96% isolated yield (product weight: 253.4 mg) following method A. ^{1}H NMR (400 MHz, $CDCl_3$) δ 7.61 (d, J = 7.7 Hz, 1H), 7.49 – 7.42 (m, 1H), 7.35 (d, J = 7.7 Hz, 1H), 7.23 (t, J = 7.4 Hz, 1H), 3.05 – 2.96 (m, 2H), 2.57 – 2.49 (m, 2H). ^{13}C NMR (101 MHz, $CDCl_3$) δ 206.81, 154.91, 136.71, 134.32, 126.95, 126.44, 123.30, 35.91, 25.51.

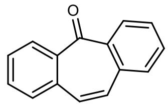

5H-dibenzo[a,d][7]annulen-5-one (2bd):[6] white solid was obtained in 90% isolated yield (product weight: 370.8 mg) following method C. ^{1}H NMR (400 MHz, $CDCl_3$) δ 8.24 – 8.19 (m, 2H), 7.66 – 7.60 (m, 2H), 7.56-7.52 (m, 4H), 7.05 (s, 2H). ^{13}C NMR (101 MHz, $CDCl_3$) δ 193.07, 138.59, 134.85, 131.91, 131.60, 130.70, 130.10, 128.80.

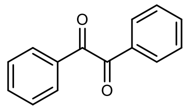

Benzil (2be):[2] pale yellow liquid was obtained in 85% isolated yield (product weight: 357.0 mg) following method B. ^{1}H NMR (400 MHz, $CDCl_3$) δ 7.98-7.95 (m, 4H), 7.67 – 7.60 (m, 2H), 7.51-7.47 (m, 4H). ^{13}C NMR (101 MHz, $CDCl_3$) δ 194.49, 134.82, 132.78, 129.74, 128.91.

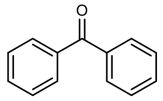

Benzophenone (2bf):[3] white solid was obtained in 97% isolated yield (product weight: 353.1 mg) following method A. ^{1}H NMR (400 MHz, $CDCl_3$) δ 7.82-7.79 (m, 4H), 7.61 – 7.56 (m, 2H), 7.51 –

7.45 (m, 4H). ^{13}C NMR (101 MHz, $CDCl_3$) δ 196.72, 137.48, 132.37, 130.00, 128.21.

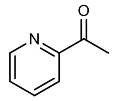

1-(Pyridin-2-yl)ethan-1-one (2bg):[7] colorless liquid was obtained in 70% isolated yield (product weight: 169.4 mg) following method B. ^{1}H NMR (400 MHz, $CDCl_3$) δ 8.70-8.69 (m, 1H), 8.09 – 8.02 (m, 1H), 7.87-7.82 (m, 1H), 7.54 – 7.46 (m, 1H), 2.73 (s, 3H). ^{13}C NMR (101 MHz, $CDCl_3$) δ 199.92, 153.36, 148.82, 136.68, 126.96, 121.46, 25.65.

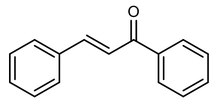

(*E*)-chalcone (2bh):[2] pale yellow liquid was obtained in 50% isolated yield (product weight: 208.0 mg) following method B. ^{1}H NMR (400 MHz, $CDCl_3$) δ 8.10 – 8.05 (m, 2H), 7.88-7.84 (m, 1H), 7.70 – 7.65 (m, 2H), 7.64 – 7.51 (m, 4H), 7.46 – 7.41 (m, 3H). ^{13}C NMR (101 MHz, $CDCl_3$) δ 190.34, 144.69, 138.02, 134.70, 132.68, 130.44, 128.83, 128.50, 128.38, 128.24, 121.86.

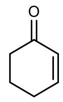

Cyclohex-2-en-1-one (2bi):[1] pale yellow liquid was obtained in 75% isolated yield (product weight: 144.0 mg) following method C. ^{1}H NMR (400 MHz, $CDCl_3$) δ 7.05-7.00 (m, 1H), 6.08 – 5.97 (m, 1H), 2.47 – 2.34 (m, 4H), 2.08 – 1.99 (m, 2H). ^{13}C NMR (101 MHz, $CDCl_3$) δ 199.45, 150.58, 129.51, 37.80, 25.37, 22.43.

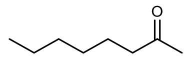

Octan-2-one (2bj):[5] pale yellow liquid was obtained in 25% isolated yield (product weight: 64.0 mg) following method A. ^{1}H NMR (400 MHz, $CDCl_3$) δ 2.43 (t, J = 7.5 Hz, 2H), 2.14 (s, 3H), 1.63 – 1.51 (m, 2H), 1.35 – 1.23 (m, 6H), 0.88 (dd, J = 9.1, 4.6 Hz, 3H). ^{13}C NMR (101 MHz, $CDCl_3$) δ 209.23, 43.63, 31.46, 29.68, 28.70, 23.66, 22.35, 13.87.

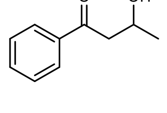

3-Hydroxy-1-phenylbutan-1-one (4):[5] colorless liquid was obtained in 78% isolated yield (product weight: 255.8 mg) following method B. ^{1}H NMR (400 MHz, $CDCl_3$) δ 7.99 – 7.93 (m, 2H), 7.61-7.57 (m, 1H), 7.50-7.50 (m, 2H), 4.47 – 4.37 (m, 1H), 3.44 (s, 1H), 3.21-3.02 (m, 2H), 1.31 (d, J = 6.4 Hz,

3H). ^{13}C NMR (101 MHz, $CDCl_3$) δ 200.82, 136.56, 133.52, 128.62, 128.00, 63.94, 46.40, 22.34.

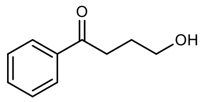

4-Hydroxy-1-phenylbutan-1-one(6):[5] colorless liquid was obtained in 85% isolated yield (product weight: 278.8 mg) following method B. ^{1}H NMR (400 MHz, $CDCl_3$) δ 7.98-7.96 (m, 2H), 7.58-7.54 (m, 1H), 7.50 – 7.42 (m, 2H), 3.73 (t, J = 6.1 Hz, 2H), 3.13 (t, J = 7.0 Hz, 2H), 2.59 (s, 1H), 2.06 – 1.96 (m, 2H). ^{13}C NMR (101 MHz, $CDCl_3$) δ 200.63, 136.66, 133.07, 128.51, 128.00, 62.03, 35.16, 26.81.

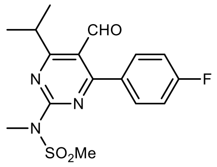

N-(4-(4-fluorophenyl)-5-formyl-6-isopropylpyrimidin-2-yl)-N-methylmethanesulfonamide (2aq):[6] white solid was obtained in 76% isolated yield (product weight: 533.5 mg) following method A. ^{1}H NMR (400 MHz, $CDCl_3$) δ 9.97 (s, 1H), 7.67 – 7.60 (m, 2H), 7.26 – 7.20 (m, 2H), 4.01 (hept, J = 6.7 Hz, 1H), 3.64 (s, 3H), 3.56 (s, 3H), 1.32 (d, J = 6.7 Hz, 6H). ^{13}C NMR (101 MHz, $CDCl_3$) δ 190.56, 179.06, 169.84, 164.48(d, J_{C-F} = 251.0 Hz), 158.81, 132.67 (d, J_{C-F} = 8.8 Hz), 132.13 (d, J_{C-F} = 3.2 Hz), 119.52, 116.02(d, J_{C-F} = 21.8 Hz), 42.55, 33.15, 32.03, 21.74. ^{19}F NMR (377 MHz, $CDCl_3$) δ -108.56.

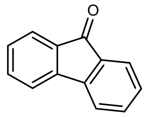

9*H*-fluoren-9-one (2bk):[1] yellow solid was obtained in 86% isolated yield (product weight: 309.6 mg) following method B. ^{1}H NMR (400 MHz, $CDCl_3$) δ 7.63-7.58 (m, 1H), 7.45 – 7.39 (m, 2H), 7.28 – 7.20 (m, 1H). ^{13}C NMR (101 MHz, $CDCl_3$) δ 193.90, 144.37, 134.68, 134.07, 129.05, 124.23, 120.32.

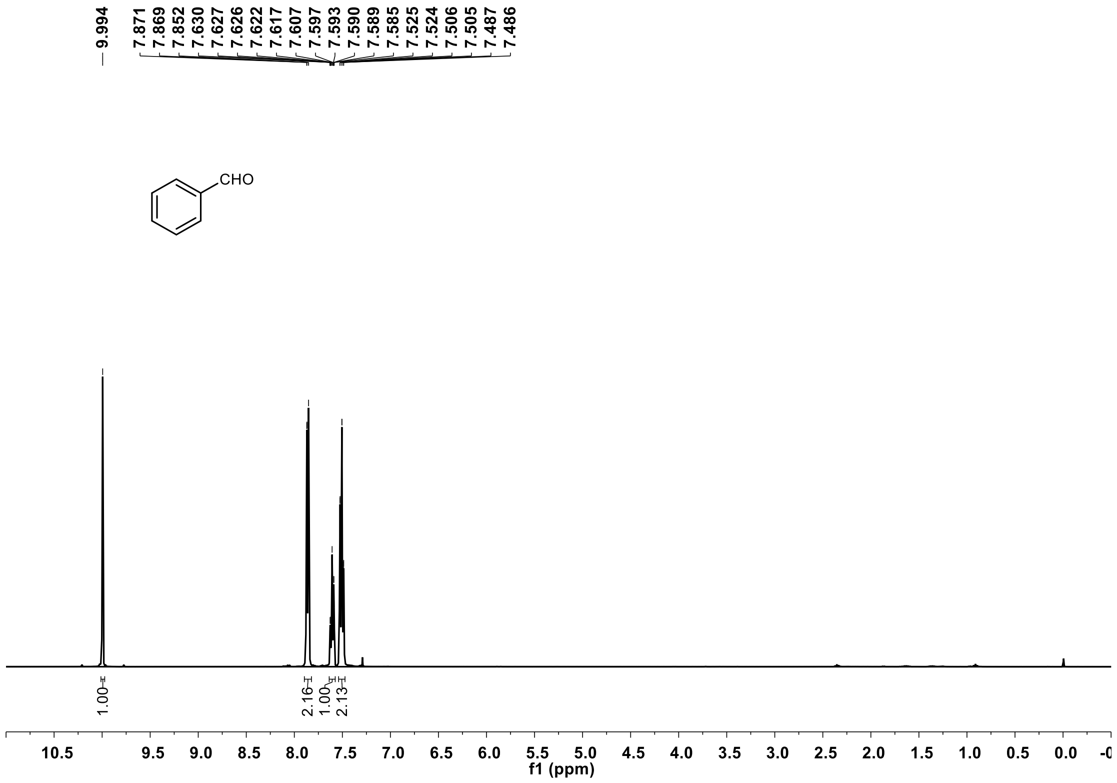

Supplementary Figure 9. **^{1}H NMR (400 MHz, $CDCl_3$) spectrum of 2aa**

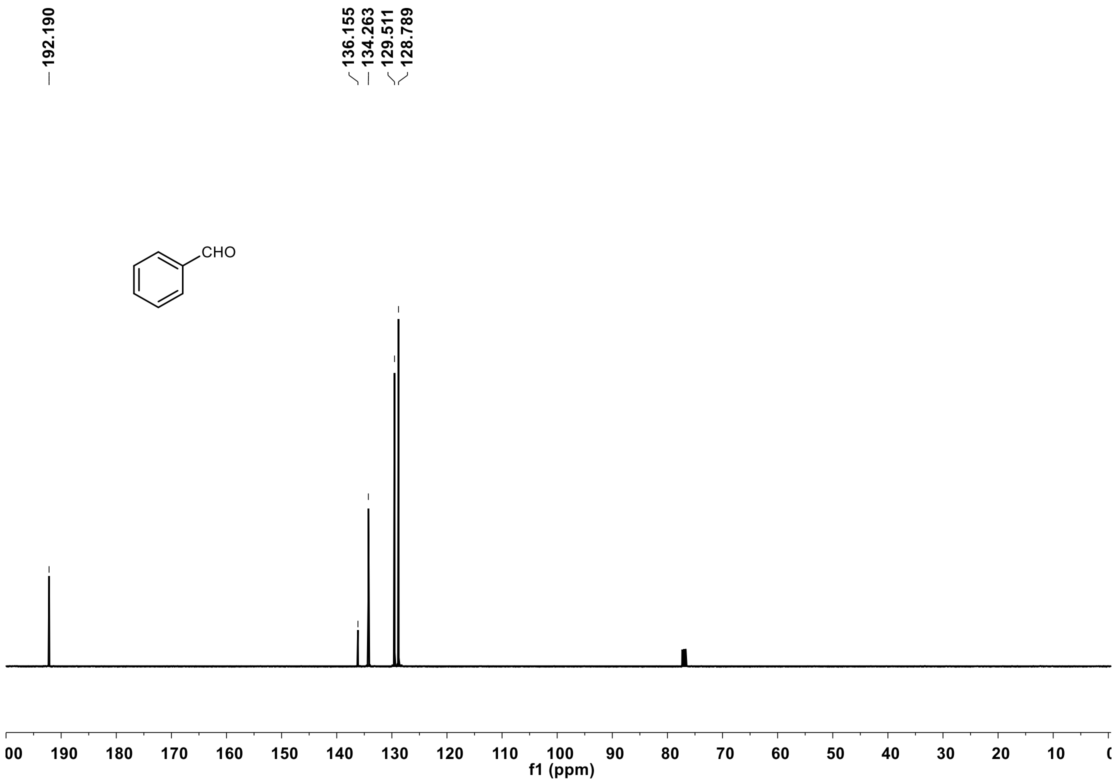

Supplementary Figure 10. **^{13}C NMR (101 MHz, $CDCl_3$) spectrum of 2aa**

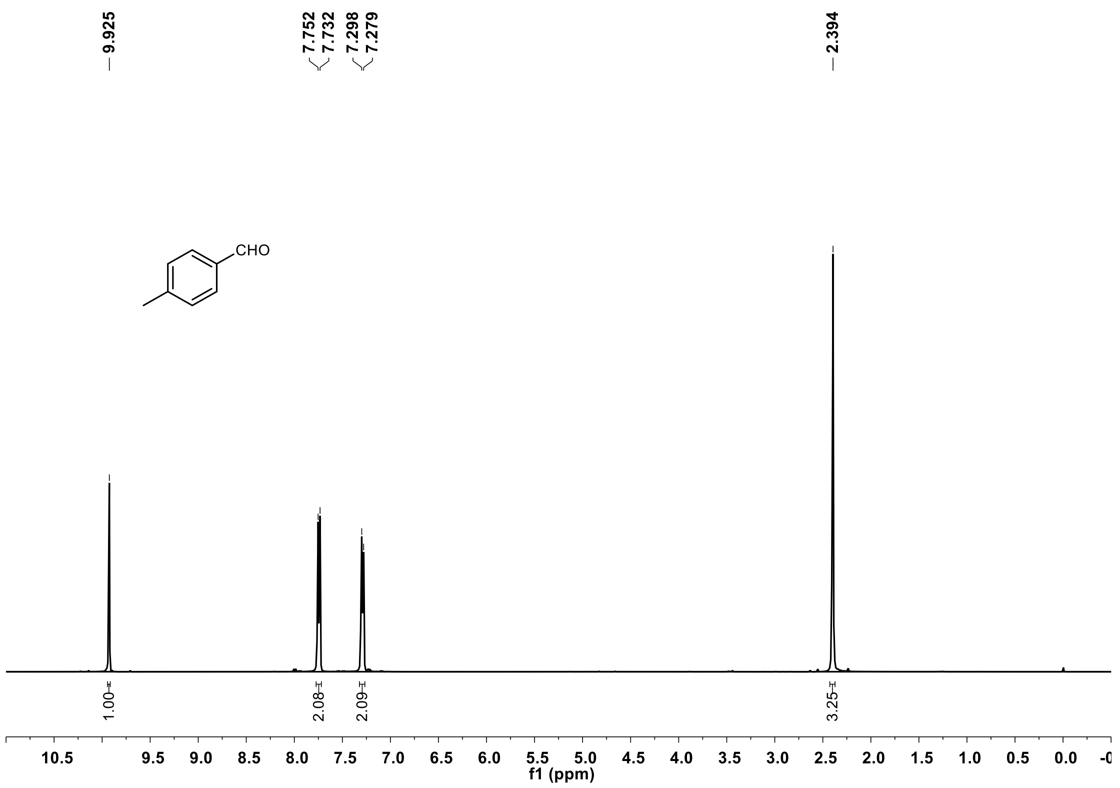

Supplementary Figure 11. ^{1}**H NMR (400 MHz,** $CDCl_3$**) spectrum of 2ab**

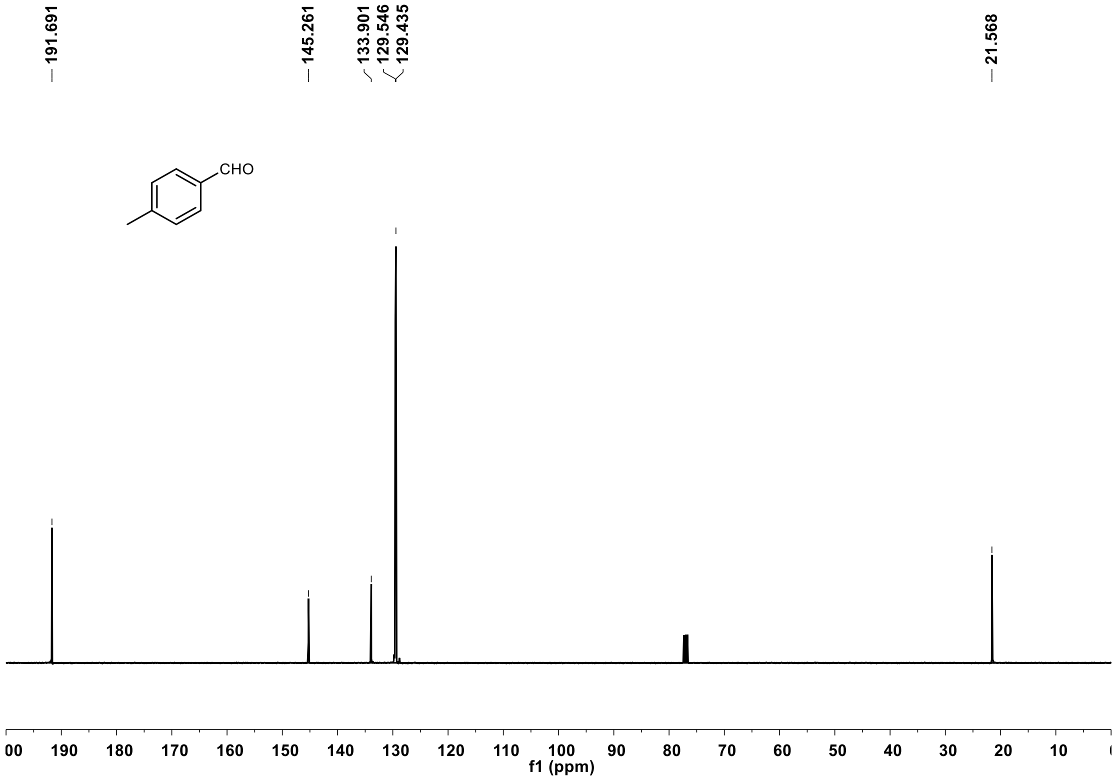

Supplementary Figure 12. ^{13}**C NMR (101 MHz,** $CDCl_3$**) spectrum of 2ab**

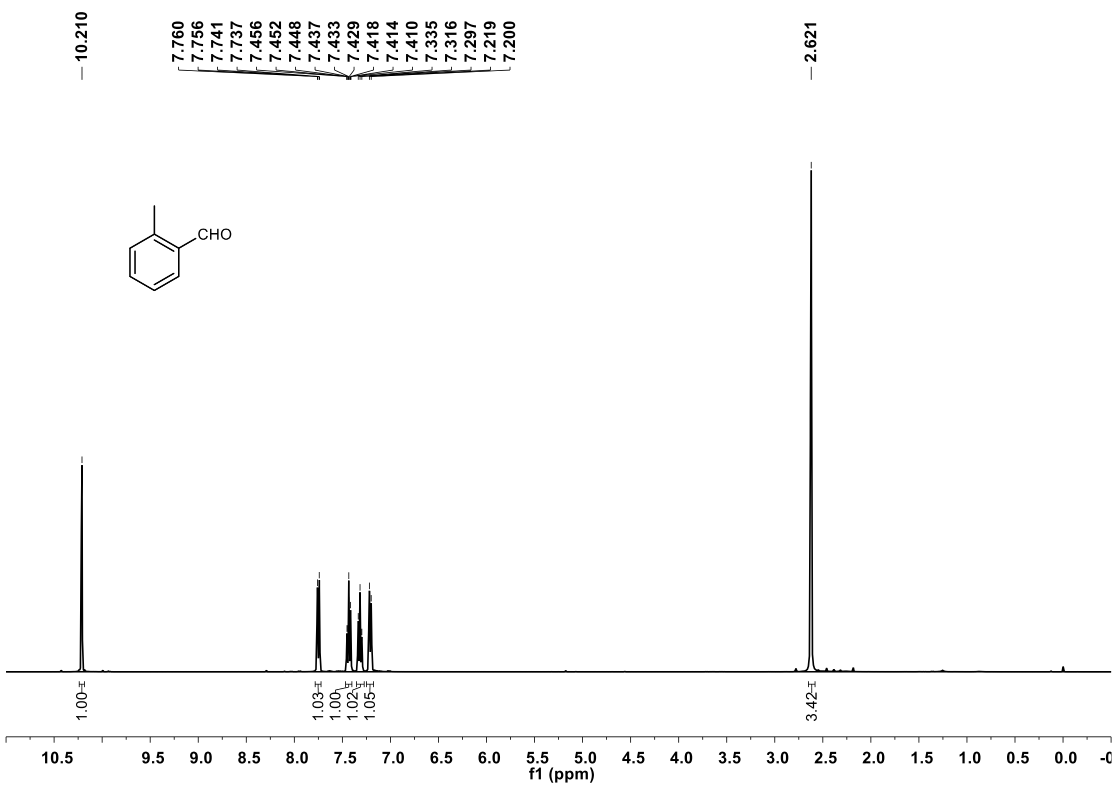

Supplementary Figure 13. ^{1}H NMR (400 MHz, $CDCl_3$) spectrum of 2ac

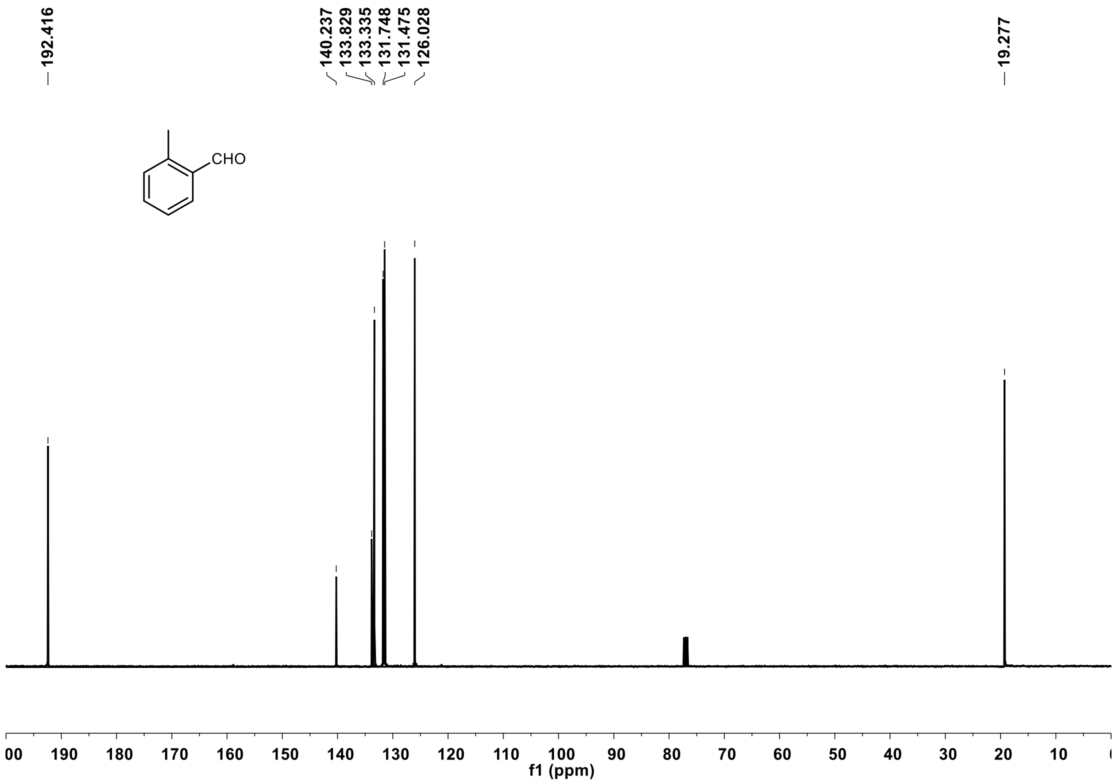

Supplementary Figure 14. ^{13}C NMR (101 MHz, $CDCl_3$) spectrum of 2ac

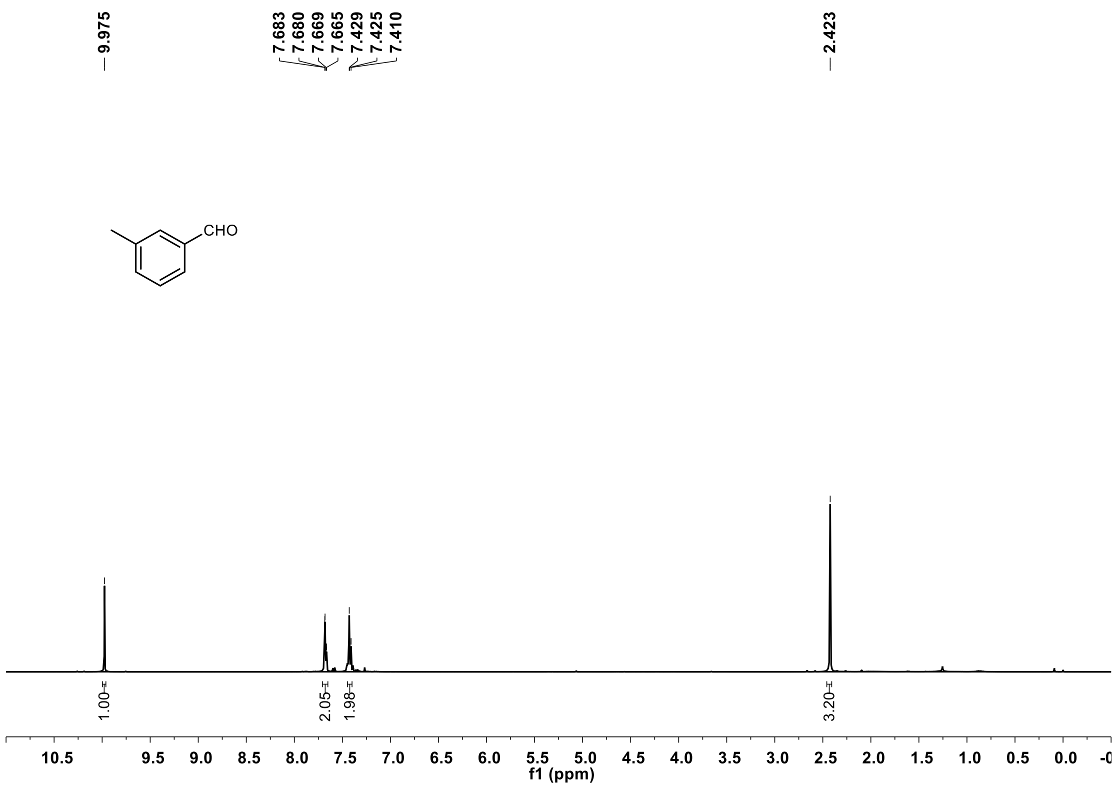

Supplementary Figure 15. ^{1}H NMR (400 MHz, $CDCl_3$) spectrum of 2ad

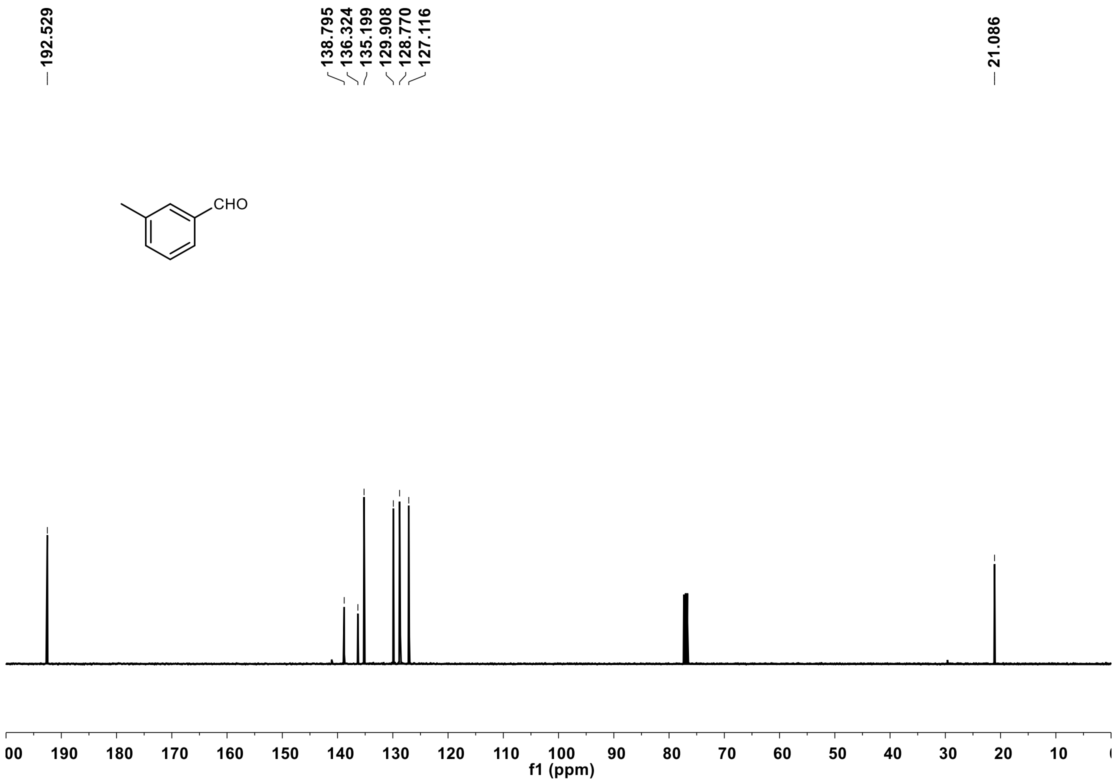

Supplementary Figure 16. ^{13}C NMR (101 MHz, $CDCl_3$) spectrum of 2ad

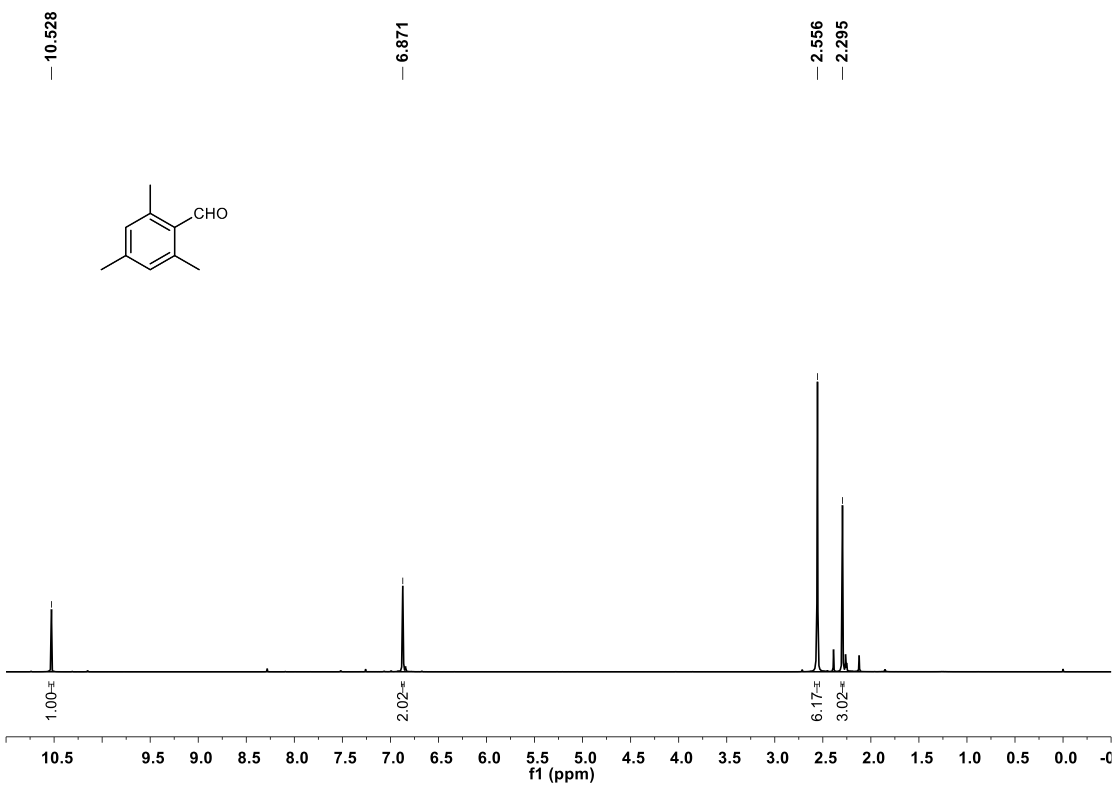

Supplementary Figure 17. ^{1}H **NMR (400 MHz,** $CDCl_3$**) spectrum of 2ae**

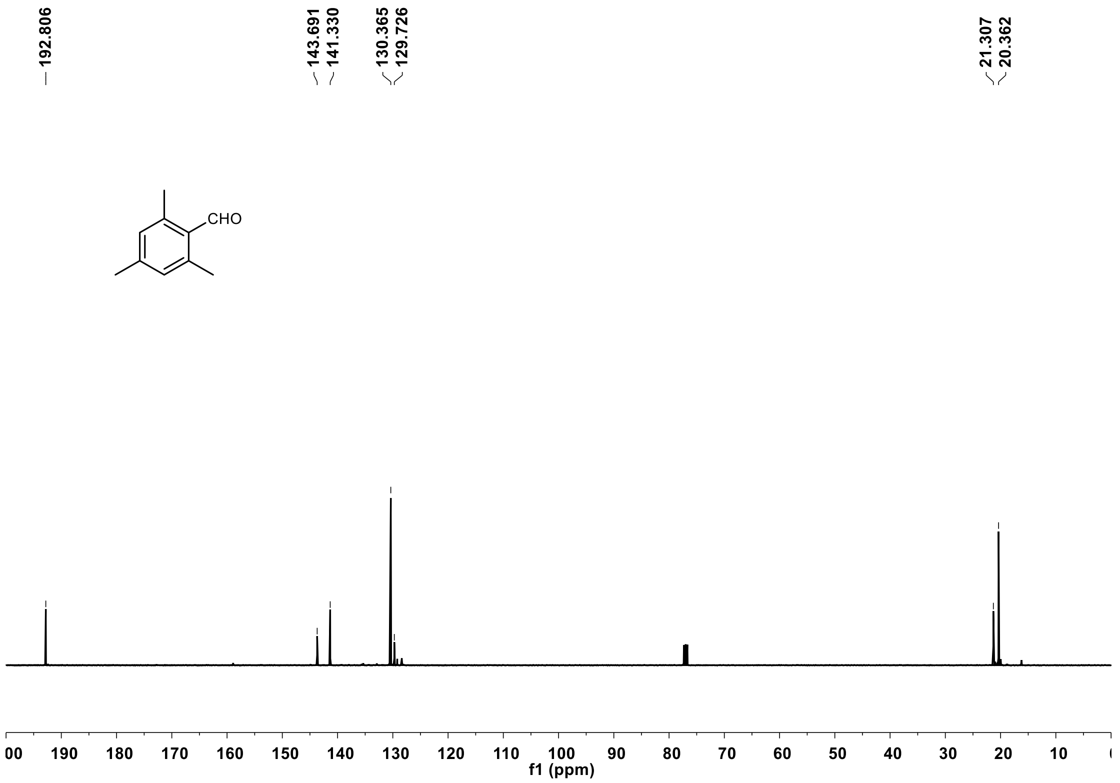

Supplementary Figure 18. ^{13}C **NMR (101 MHz,** $CDCl_3$**) spectrum of 2ae**

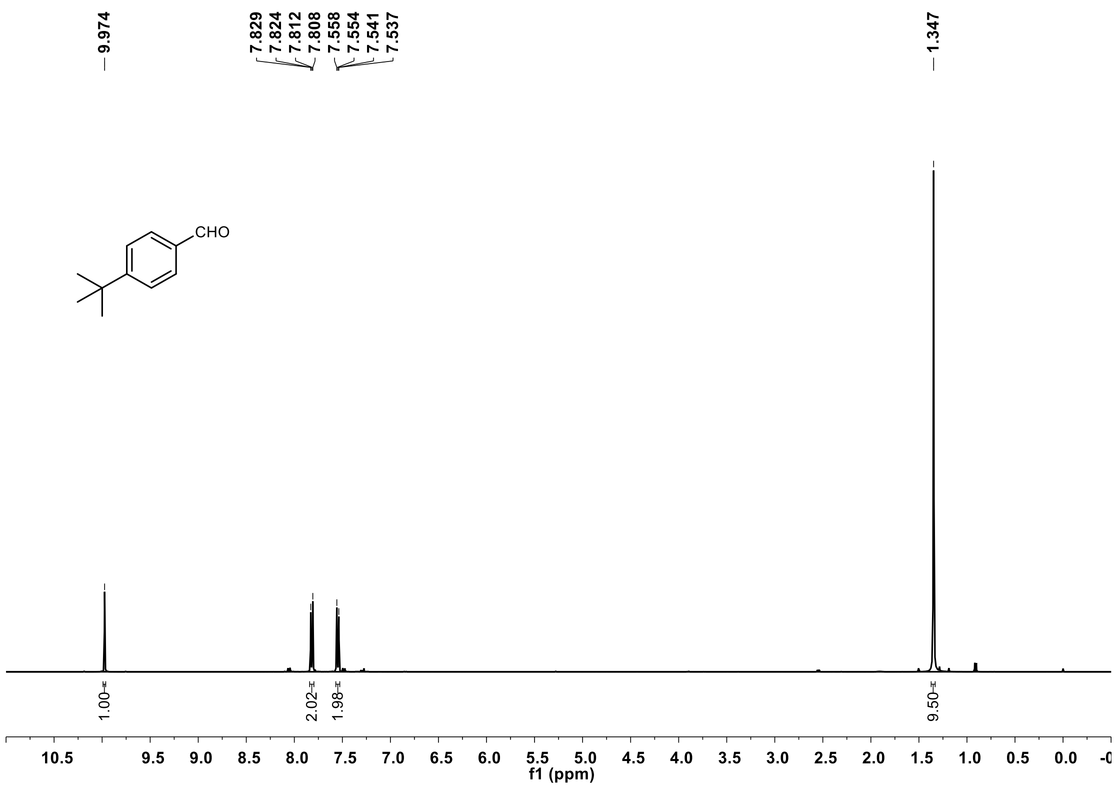

Supplementary Figure 19. ^{1}**H NMR (400 MHz,** $CDCl_3$**) spectrum of 2af**

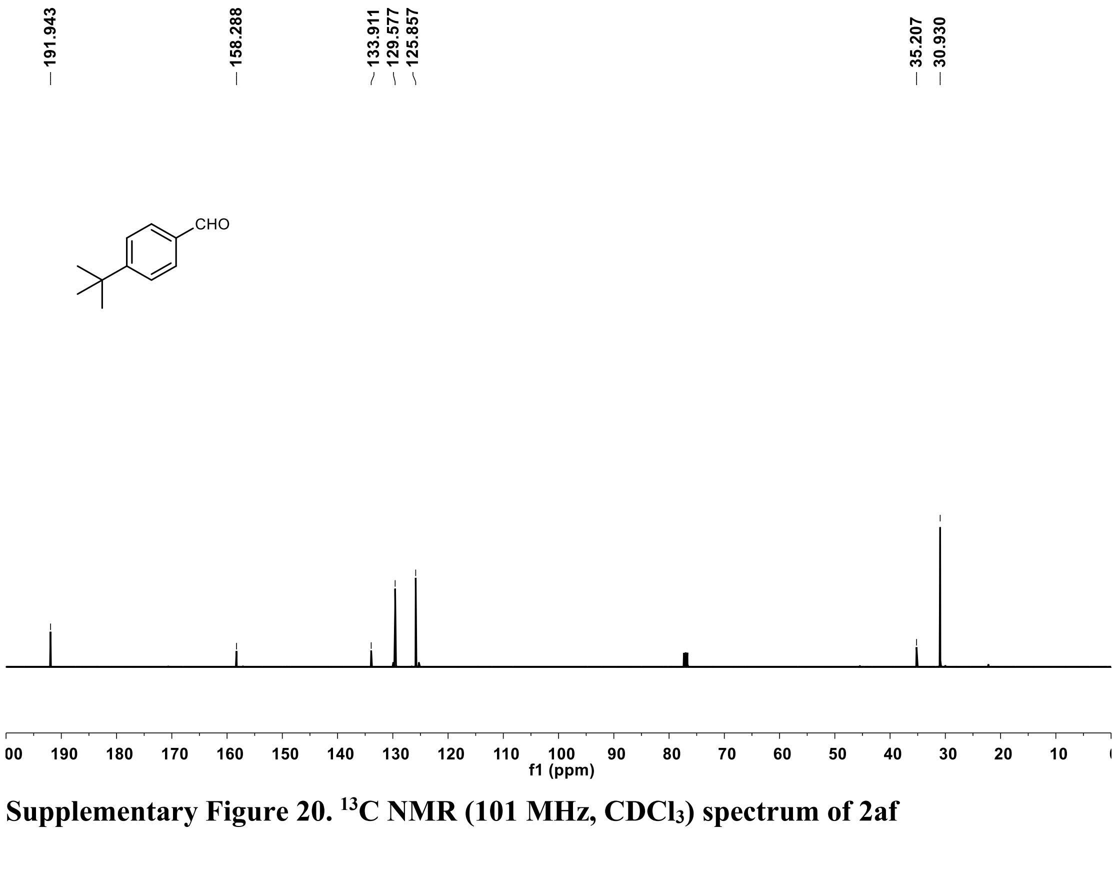

Supplementary Figure 20. ^{13}**C NMR (101 MHz,** $CDCl_3$**) spectrum of 2af**

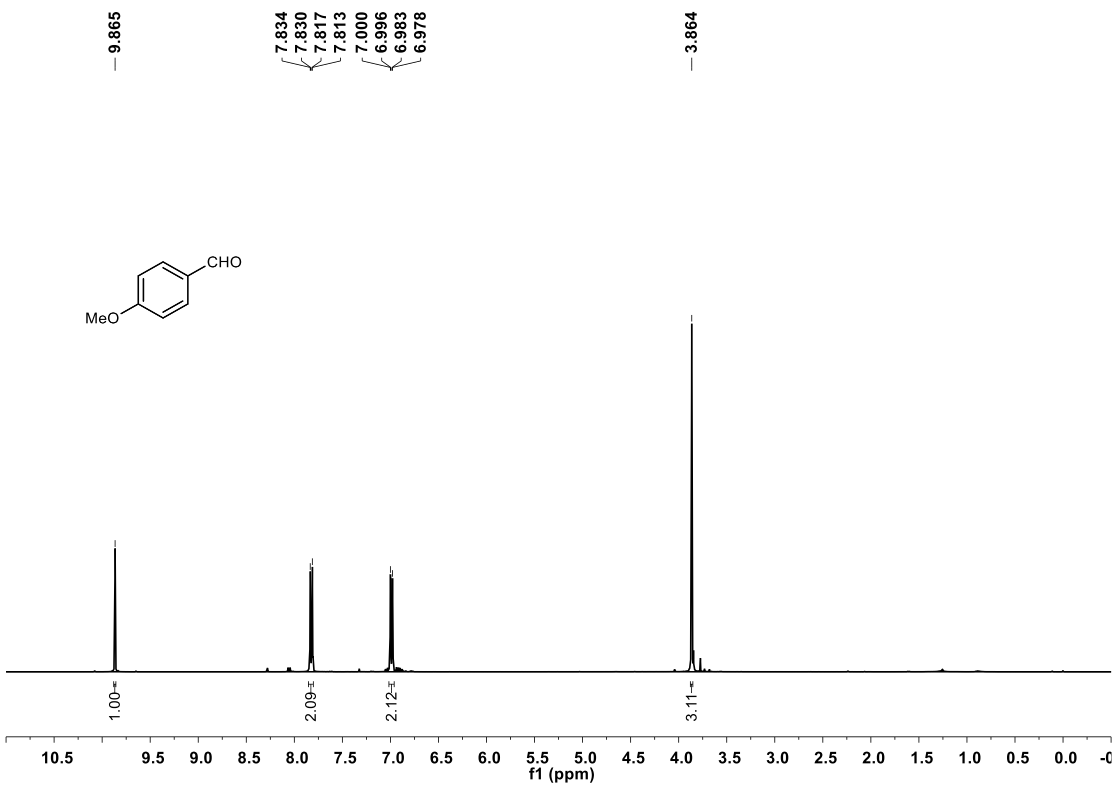

Supplementary Figure 21. ^{1}H NMR (400 MHz, $CDCl_3$) spectrum of 2ag

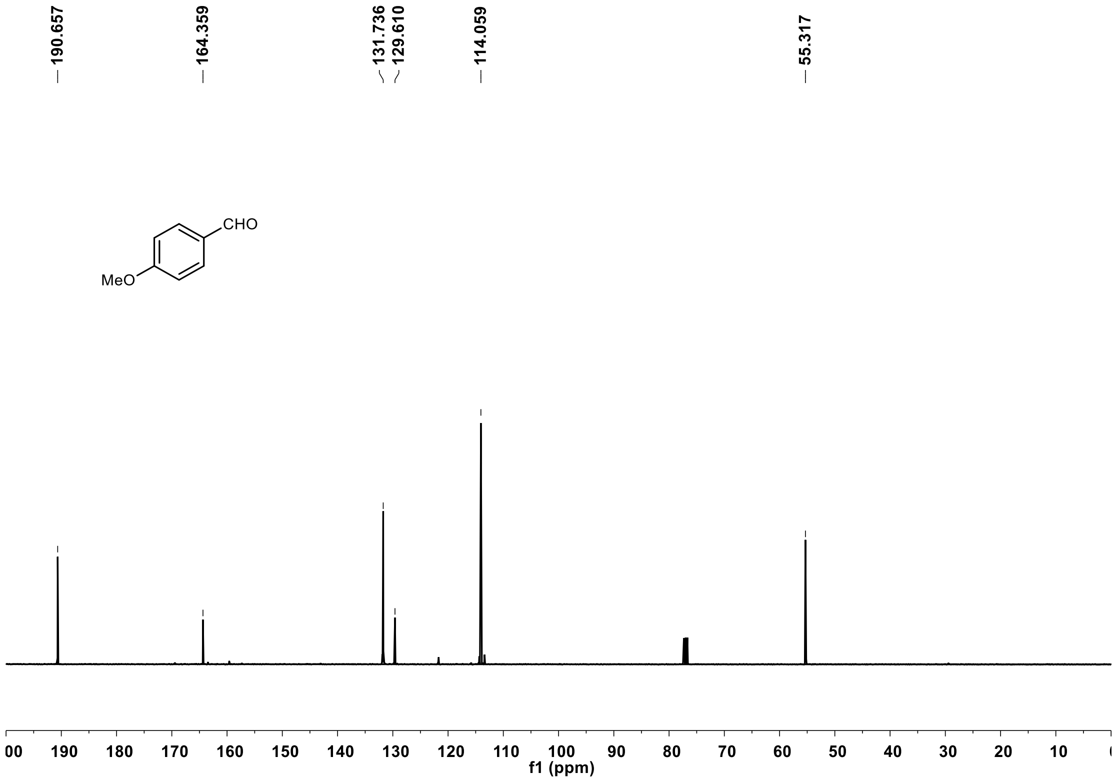

Supplementary Figure 22. ^{13}C NMR (101 MHz, $CDCl_3$) spectrum of 2ag

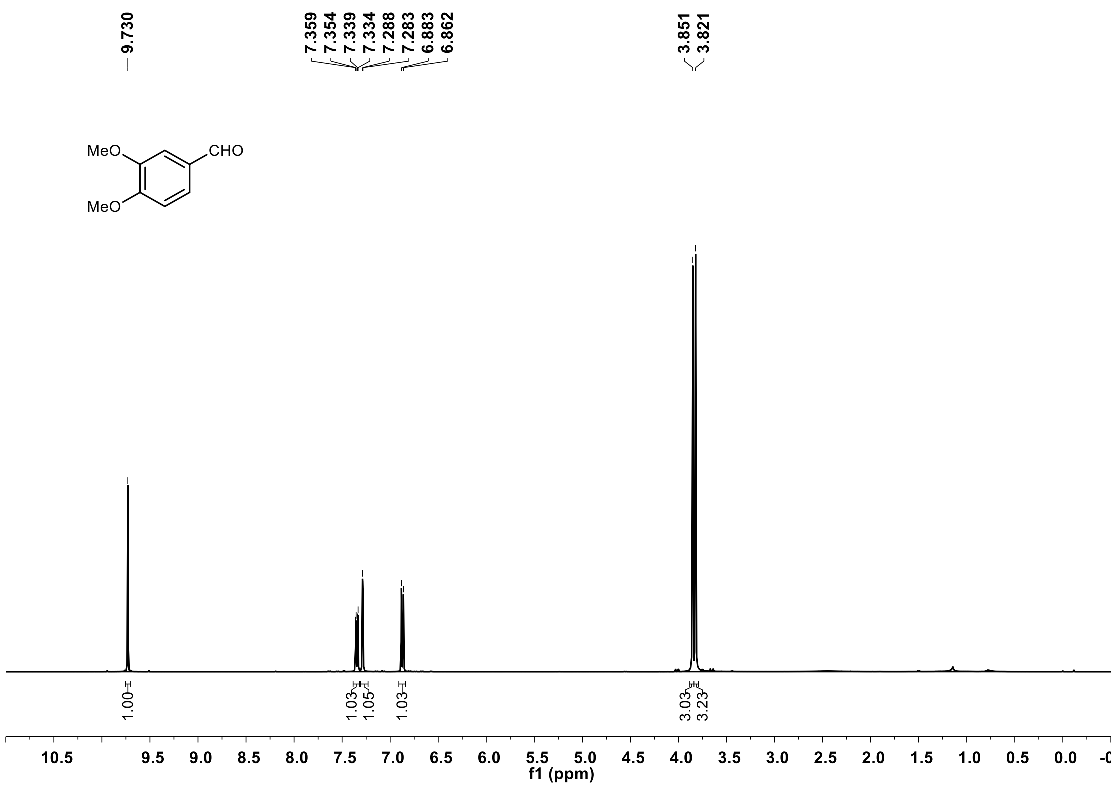

Supplementary Figure 23. ^{1}H **NMR (400 MHz,** $CDCl_3$**) spectrum of 2ah**

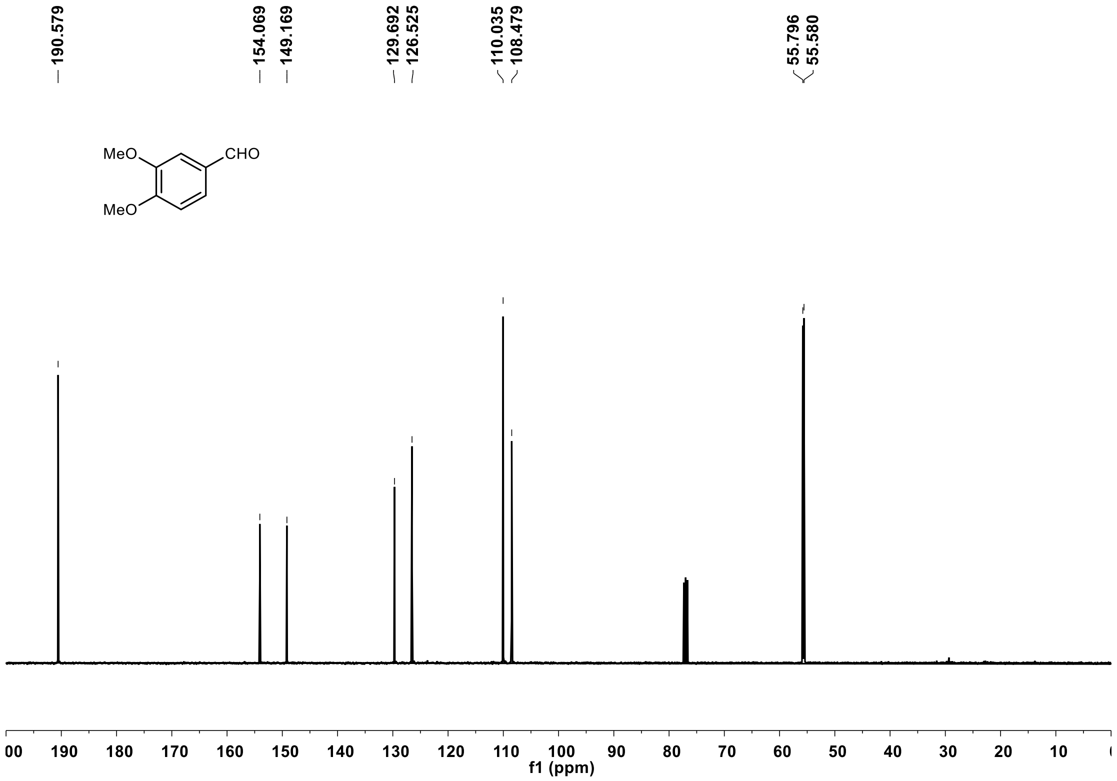

Supplementary Figure 24. ^{13}C **NMR (101 MHz,** $CDCl_3$**) spectrum of 2ah**

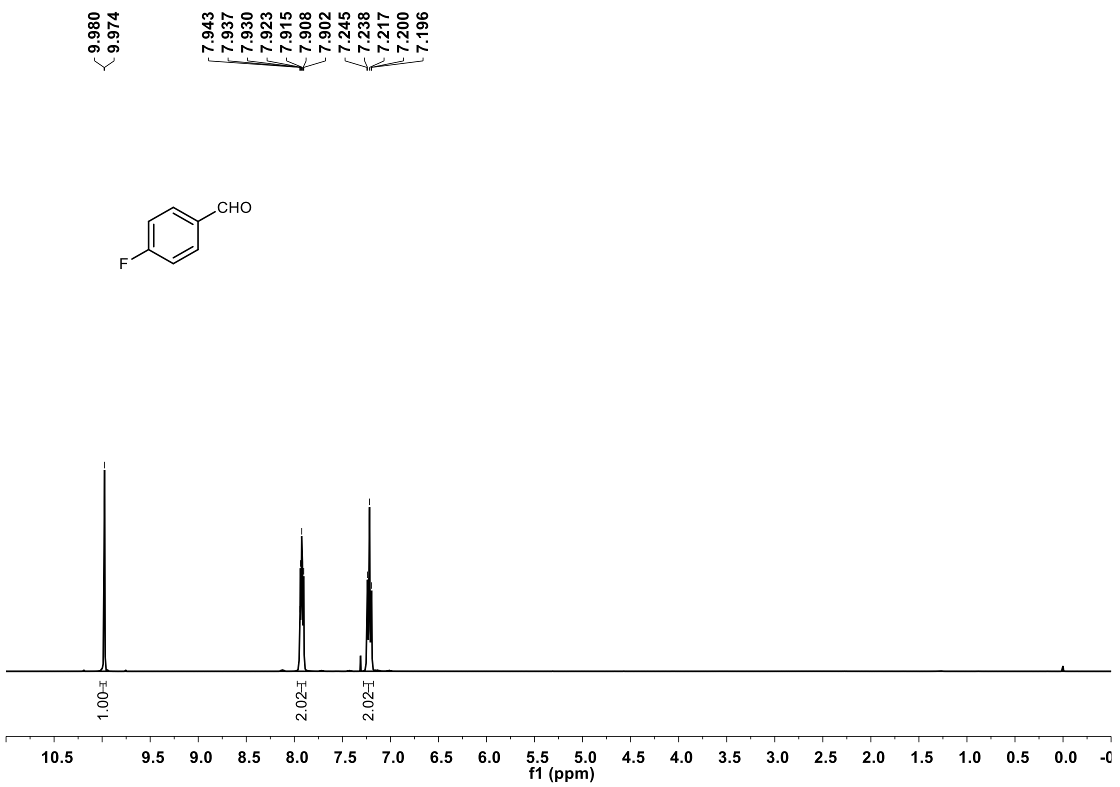

Supplementary Figure 25. ^{1}**H NMR (400 MHz,** $CDCl_3$**) spectrum of 2ai**

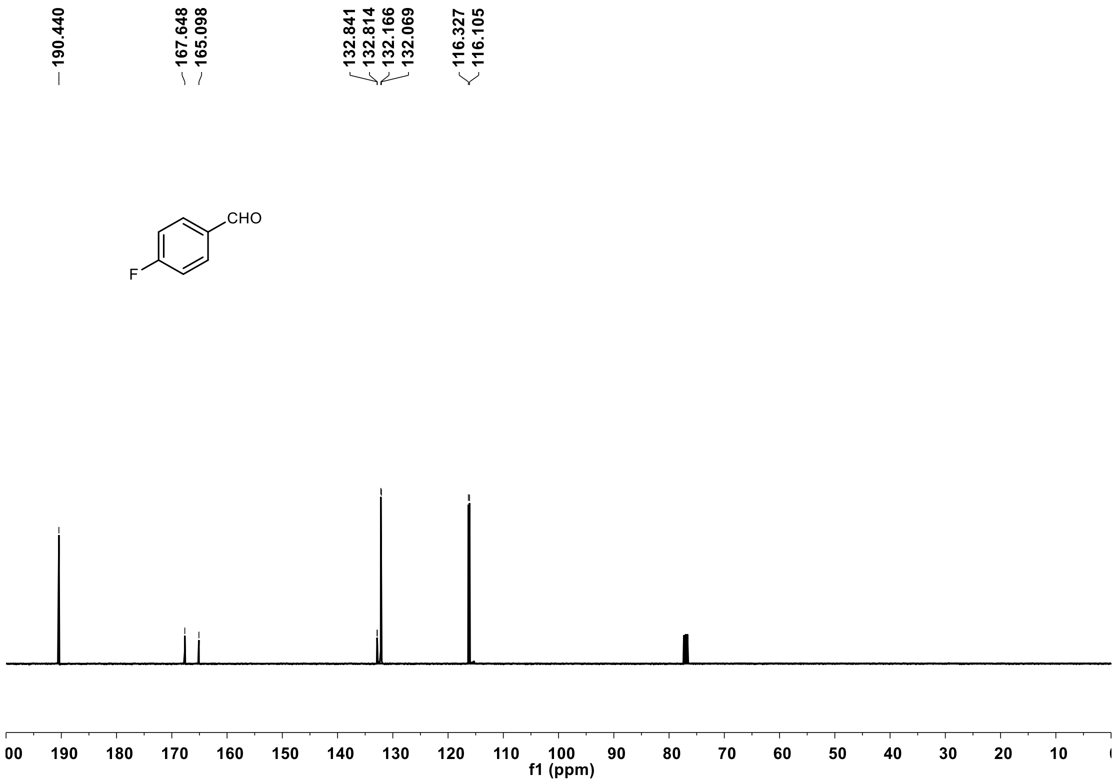

Supplementary Figure 26. ^{13}**C NMR (101 MHz,** $CDCl_3$**) spectrum of 2ai**

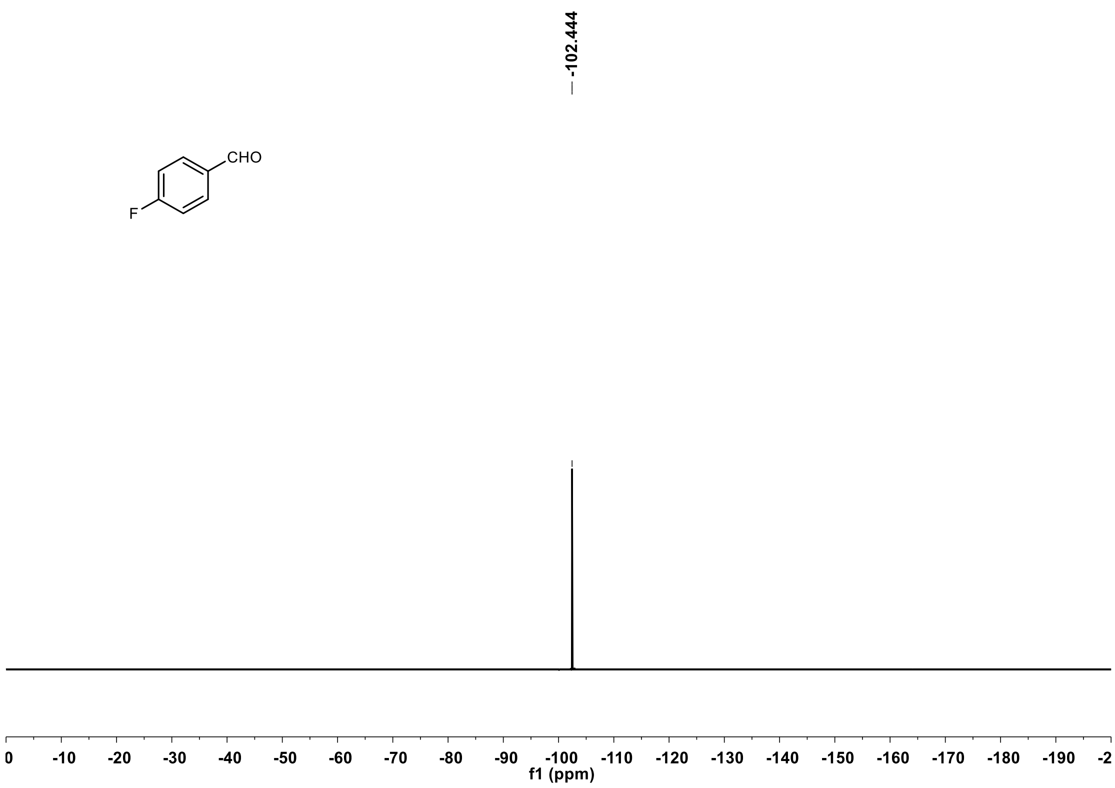

Supplementary Figure 27. ^{19}F NMR (376 MHz, $CDCl_3$) spectrum of 2ai

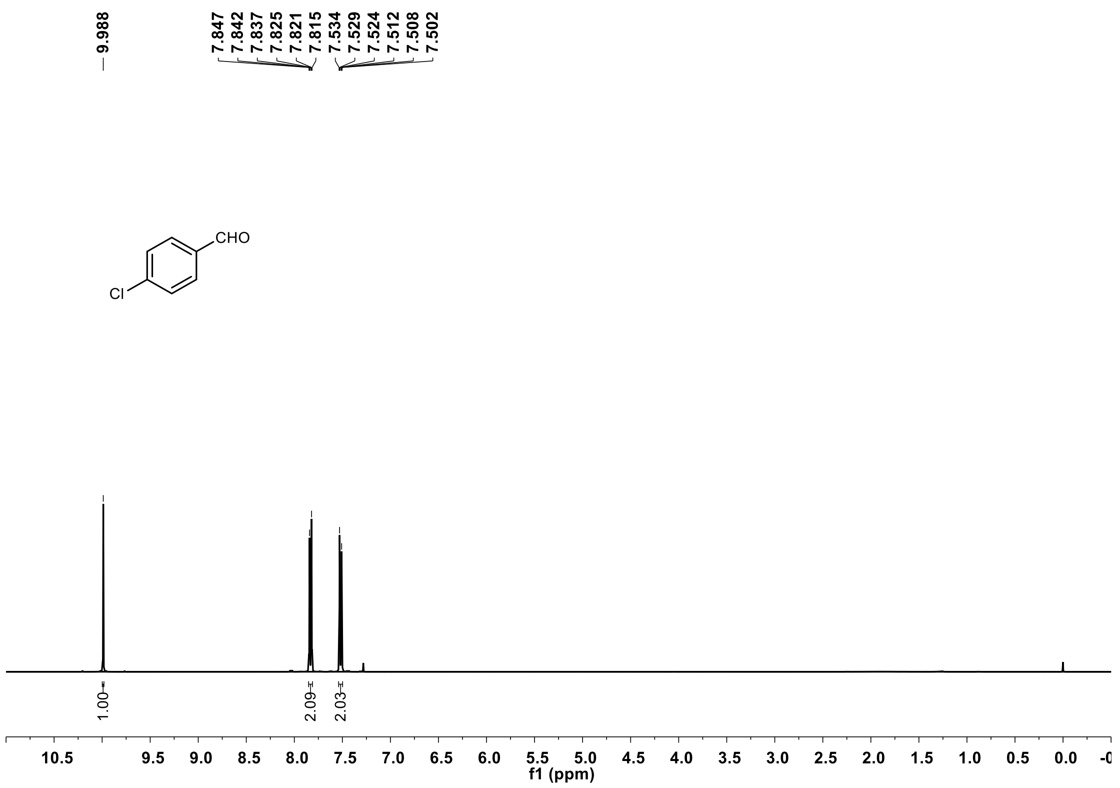

Supplementary Figure 28. ^{1}H NMR (400 MHz, $CDCl_3$) spectrum of 2aj

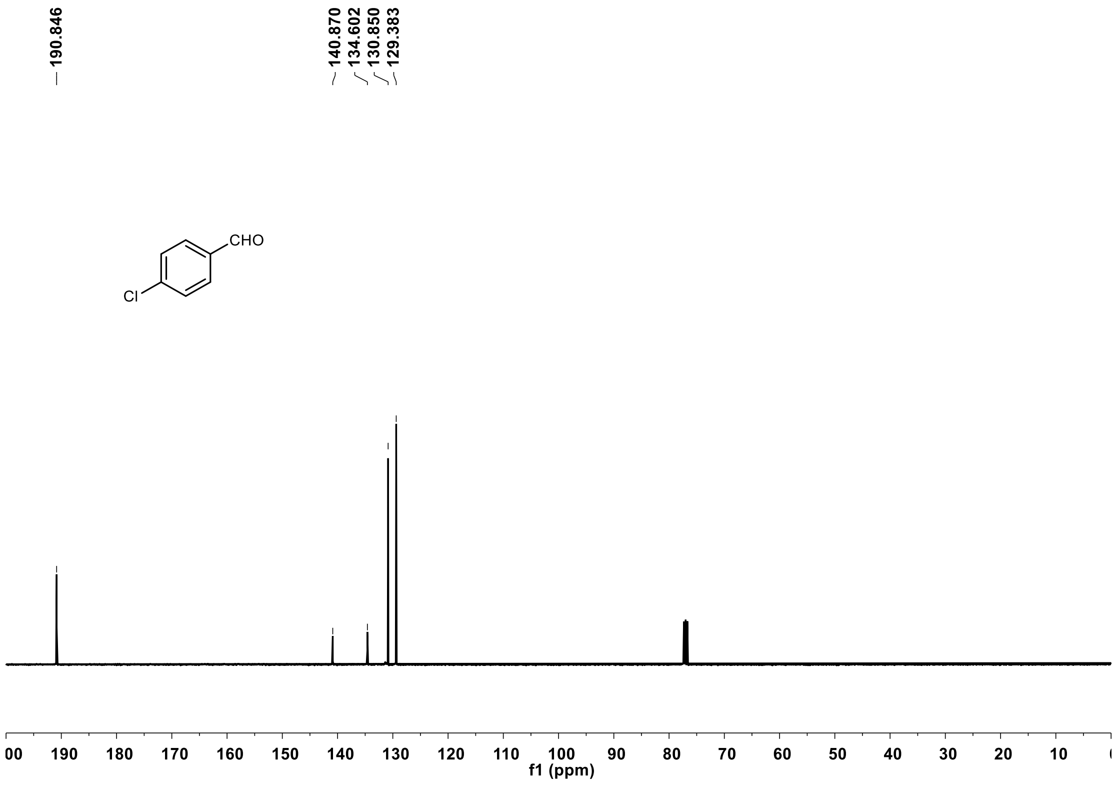

Supplementary Figure 29. ^{13}C NMR (101 MHz, $CDCl_3$) spectrum of 2aj

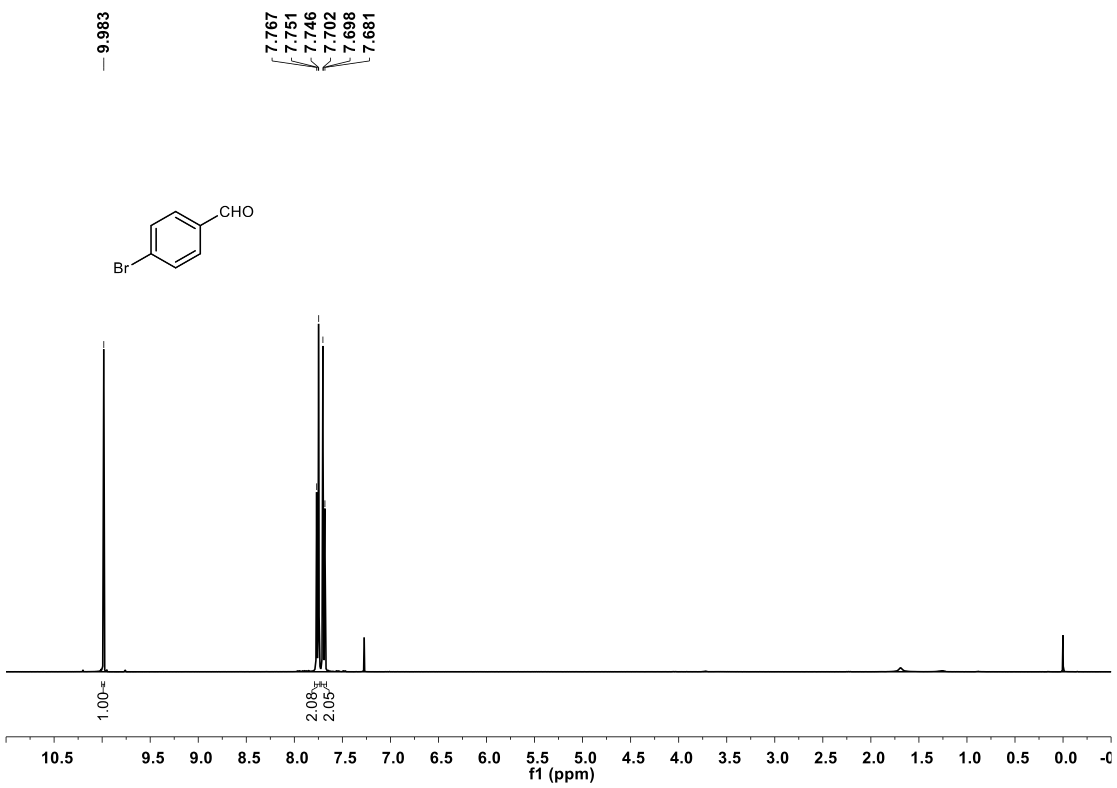

Supplementary Figure 30. **^{1}H NMR (400 MHz, $CDCl_3$) spectrum of 2ak**

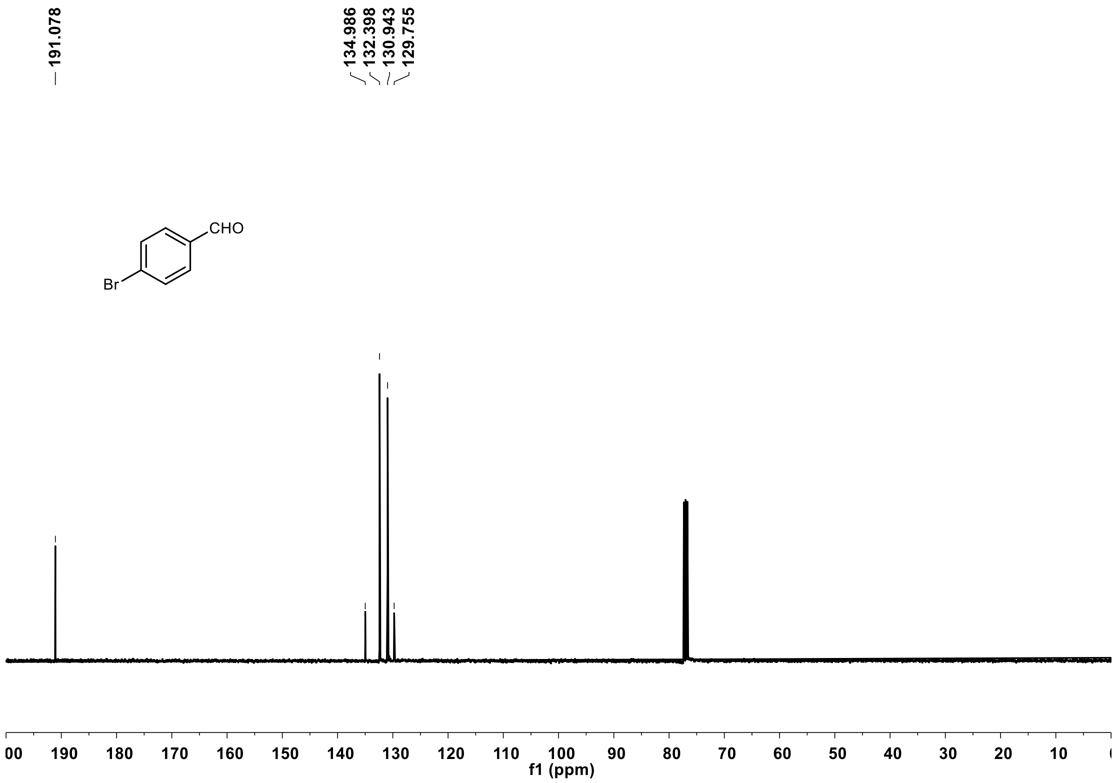

Supplementary Figure 31. **^{13}C NMR (101 MHz, $CDCl_3$) spectrum of 2ak**

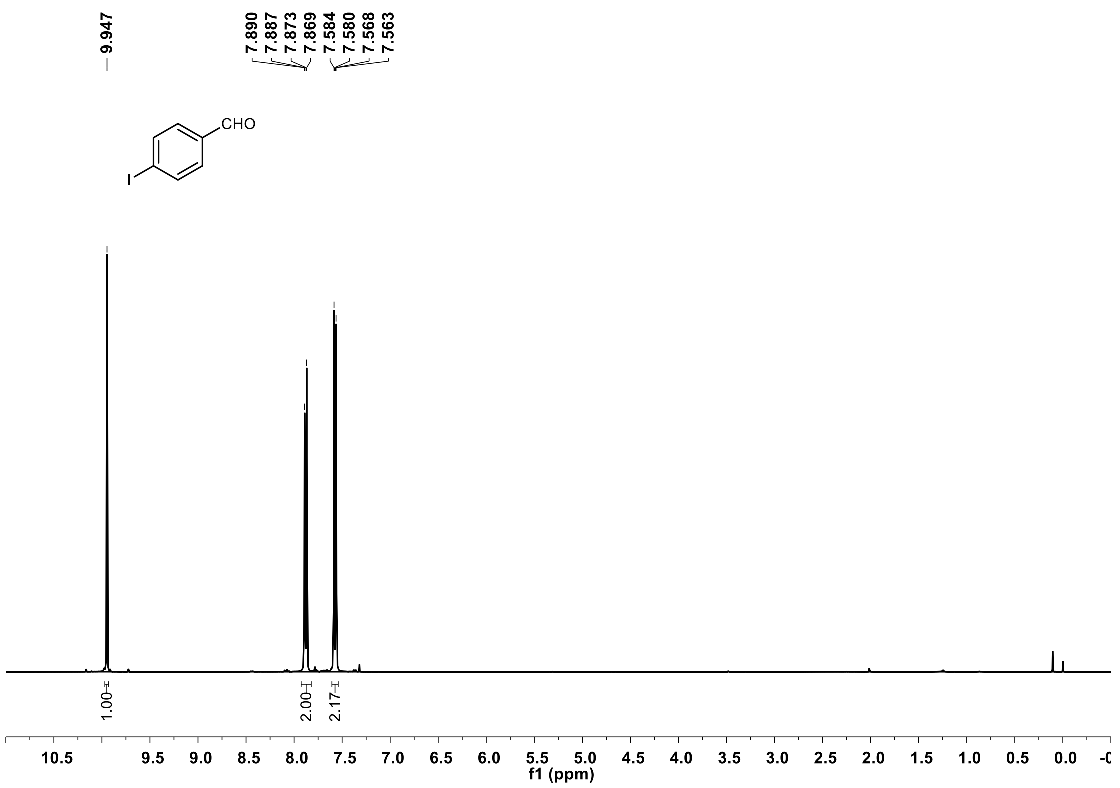

Supplementary Figure 32. ^{1}H **NMR (400 MHz,** $CDCl_3$**) spectrum of 2al**

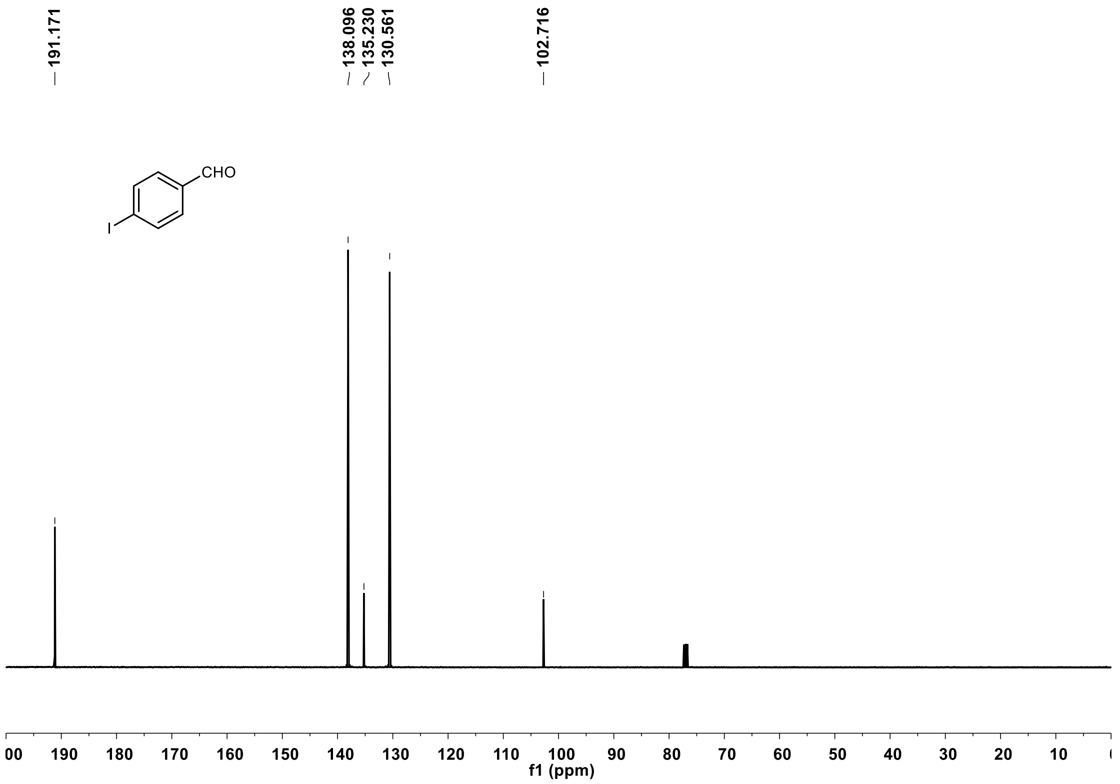

Supplementary Figure 33. ^{13}C **NMR (101 MHz,** $CDCl_3$**) spectrum of 2al**

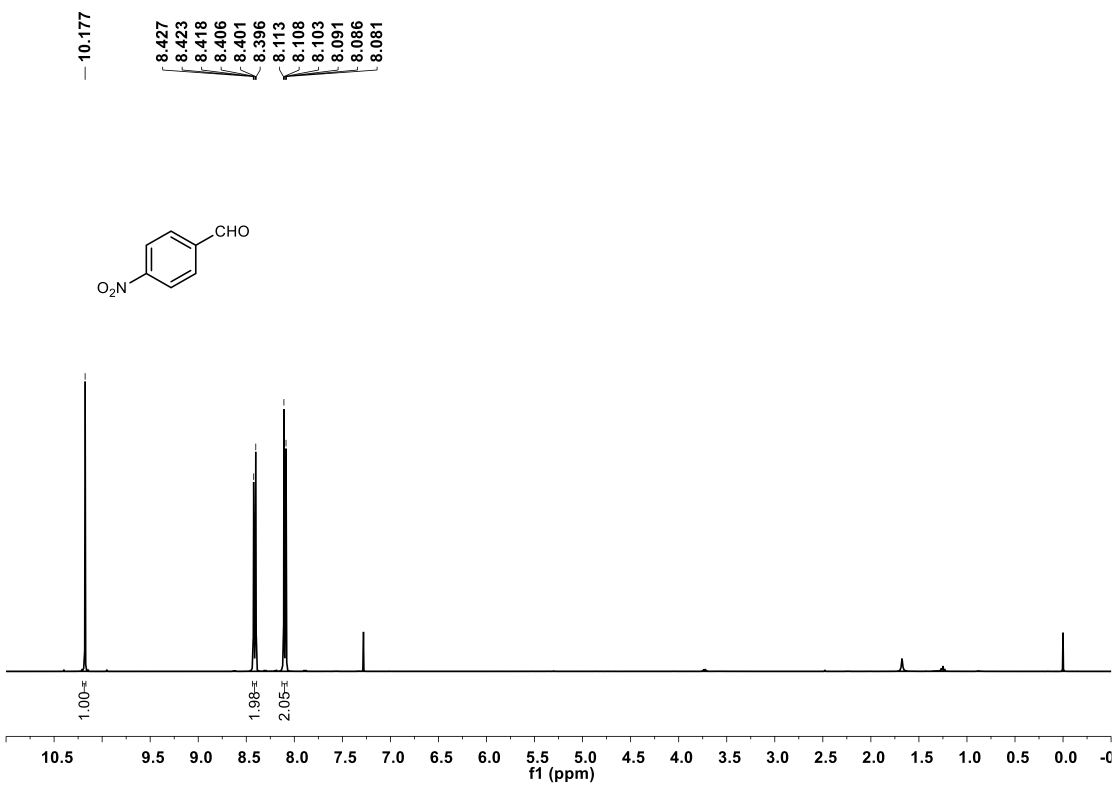

Supplementary Figure 34. ^{1}H NMR (400 MHz, $CDCl_3$) spectrum of 2am

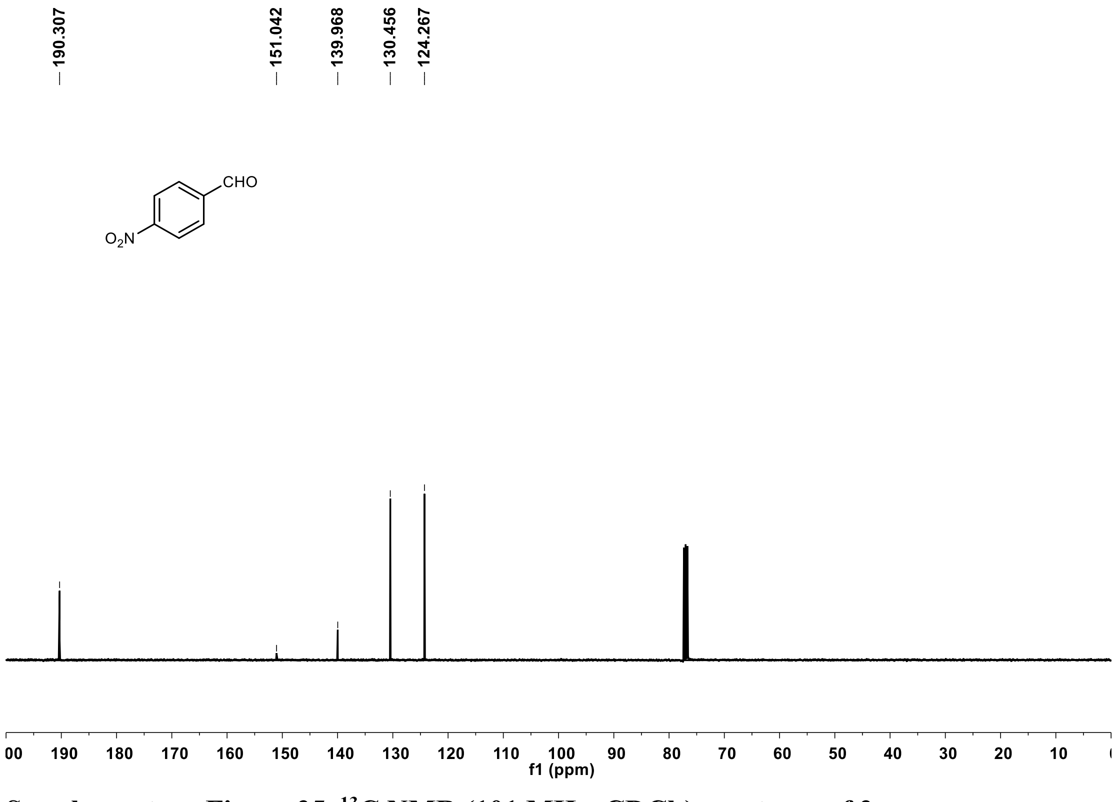

Supplementary Figure 35. ^{13}C NMR (101 MHz, $CDCl_3$) spectrum of 2am

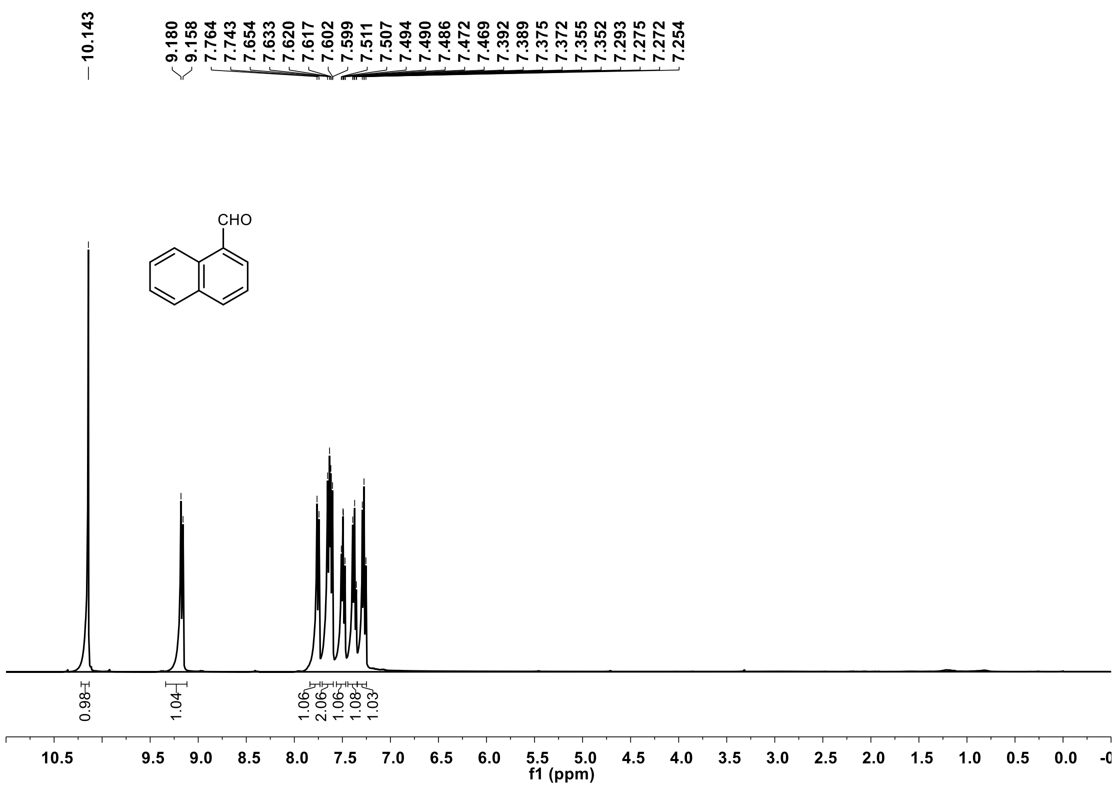

Supplementary Figure 36. ^{1}H NMR (400 MHz, $CDCl_3$) spectrum of 2an

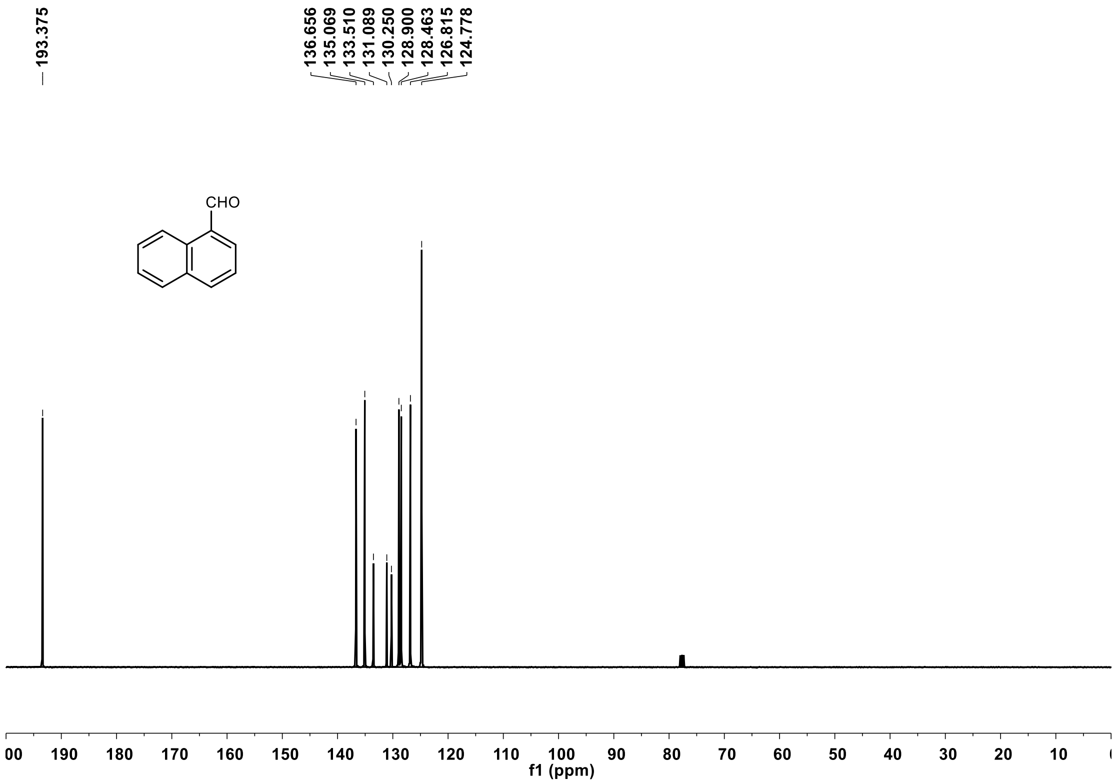

Supplementary Figure 37. ^{13}C NMR (101 MHz, $CDCl_3$) spectrum of 2an

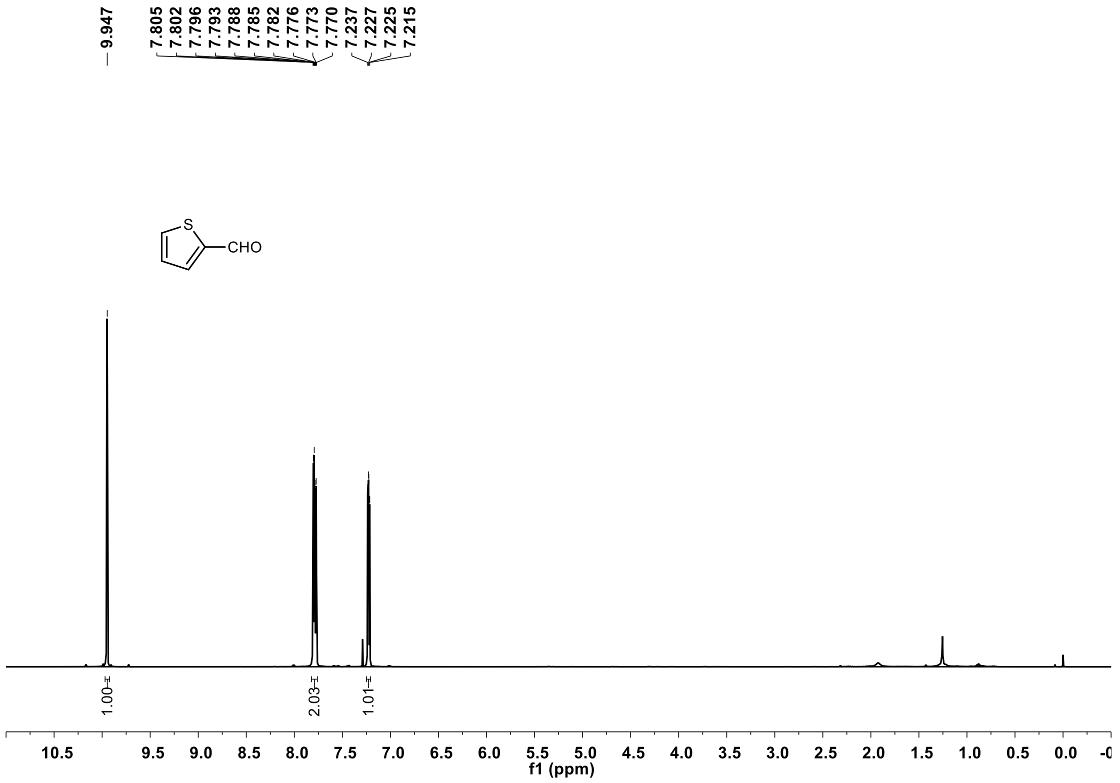

Supplementary Figure 38. ^{1}H NMR (400 MHz, $CDCl_3$) spectrum of 2ao

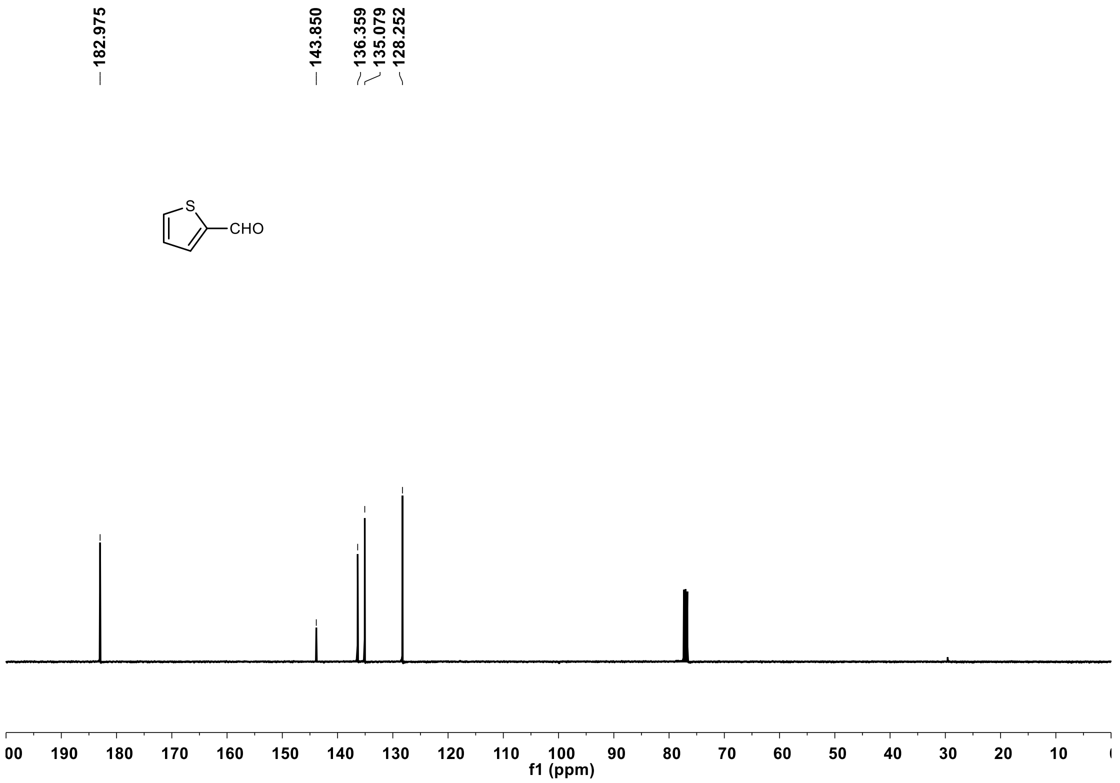

Supplementary Figure 39. ^{13}C NMR (101 MHz, $CDCl_3$) spectrum of 2ao

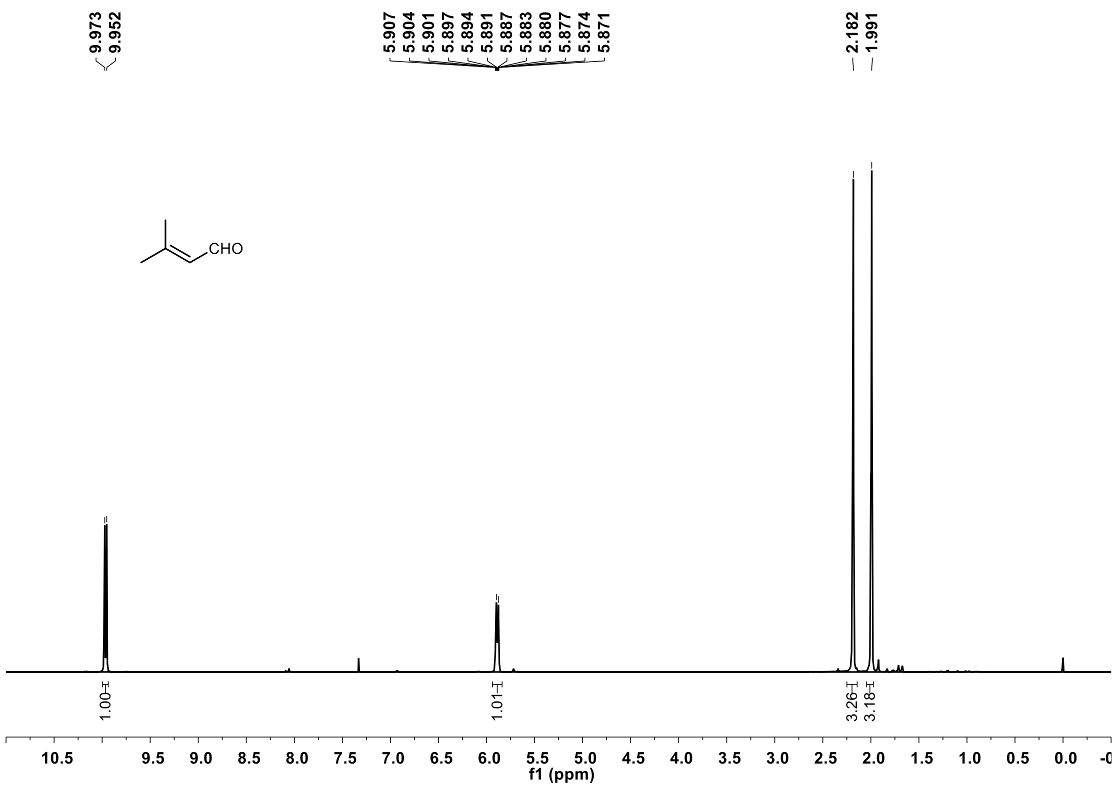

Supplementary Figure 40. ^{1}H **NMR (400 MHz,** $CDCl_3$**) spectrum of 2ap**

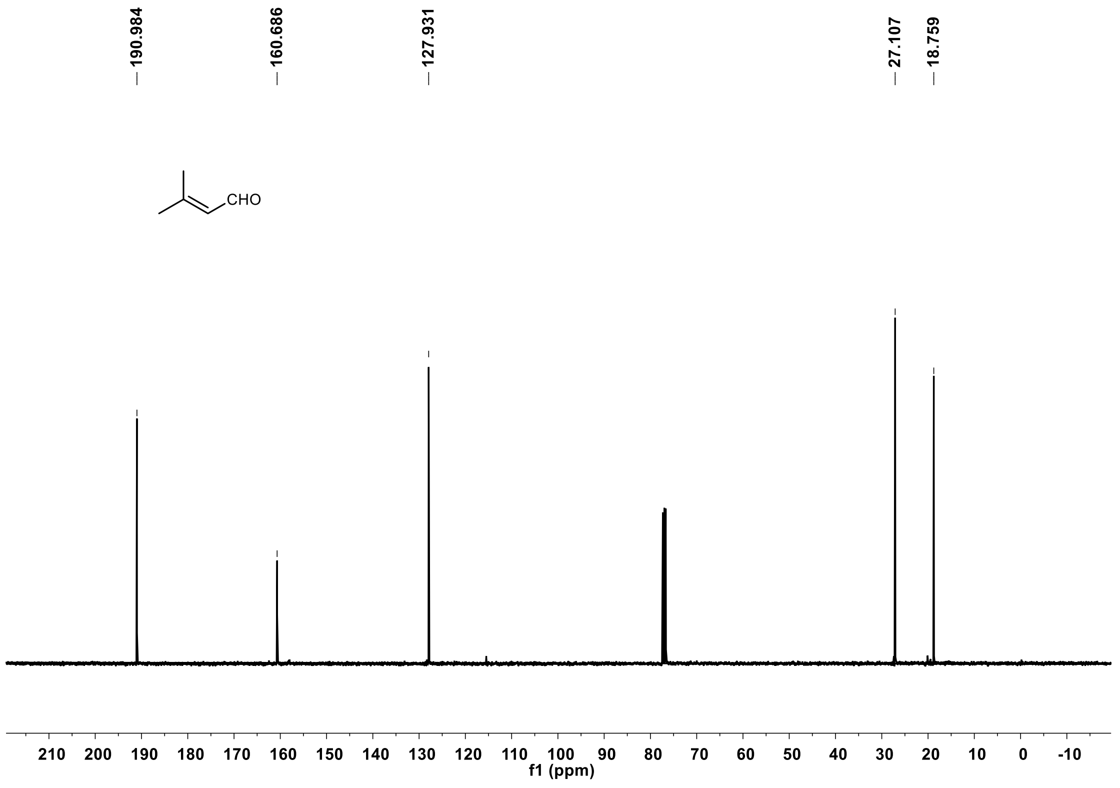

Supplementary Figure 41. ^{13}C **NMR (101 MHz,** $CDCl_3$**) spectrum of 2ap**

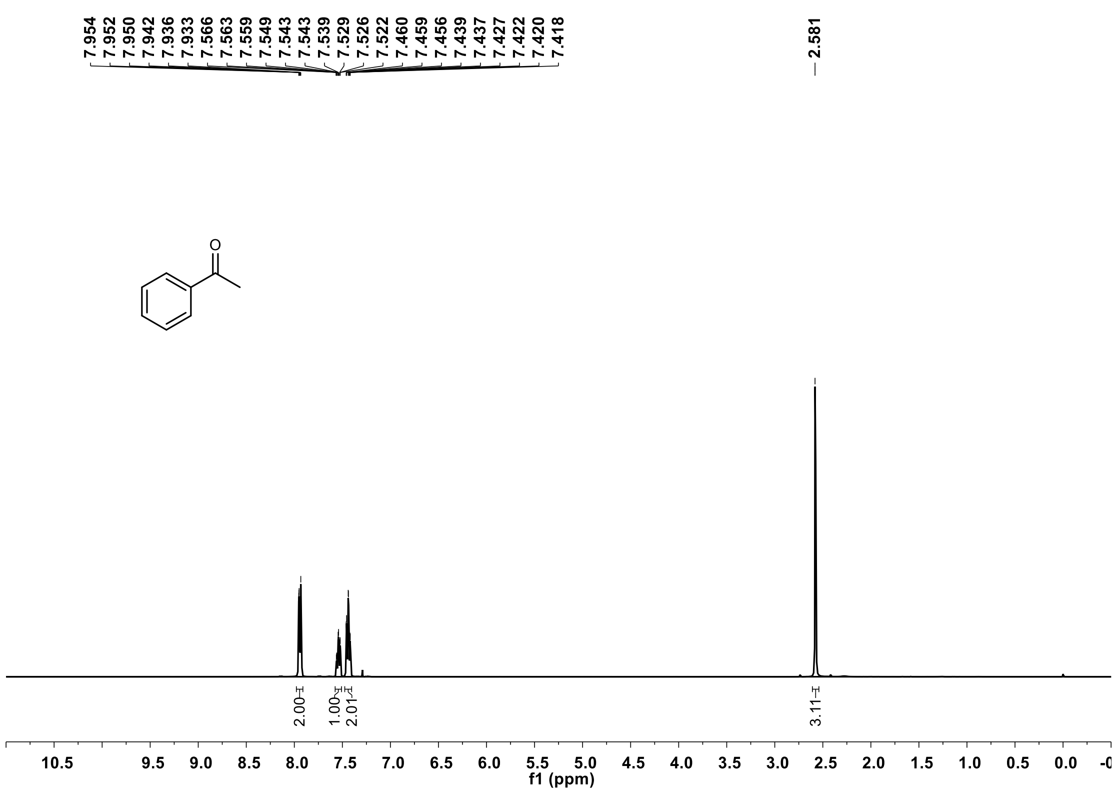

Supplementary Figure 42. ^{1}H NMR (400 MHz, $CDCl_3$) spectrum of 2ba

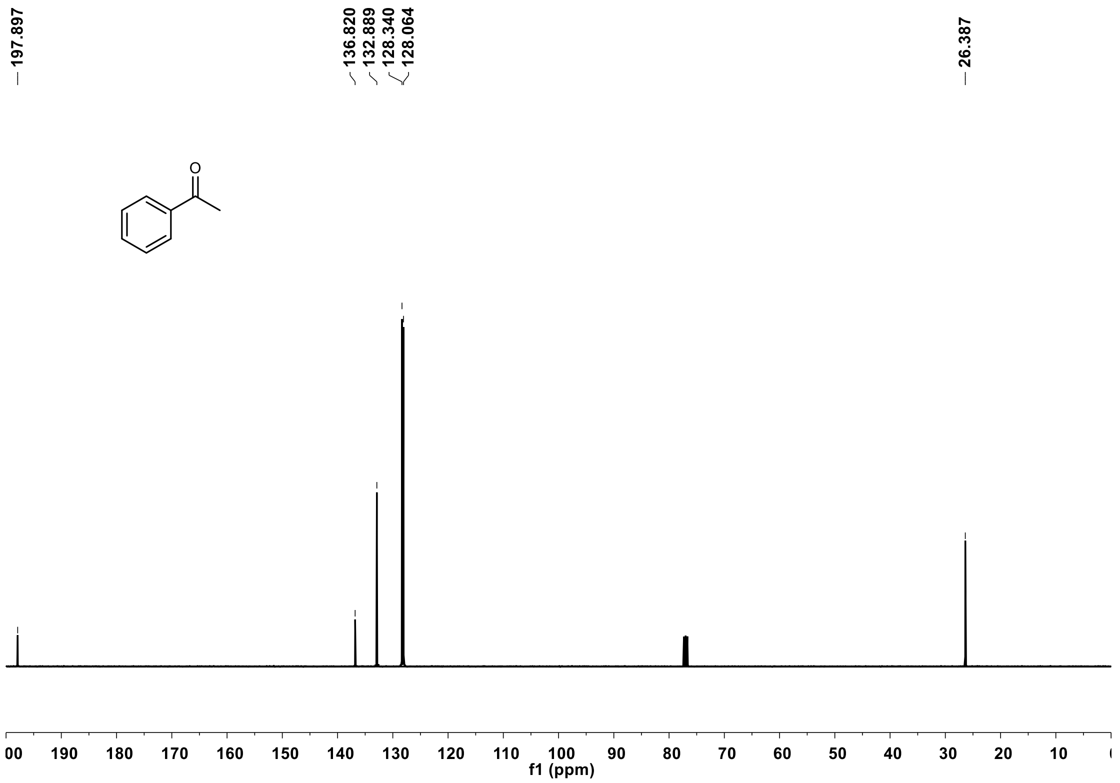

Supplementary Figure 43. ^{13}C NMR (101 MHz, $CDCl_3$) spectrum of 2ba

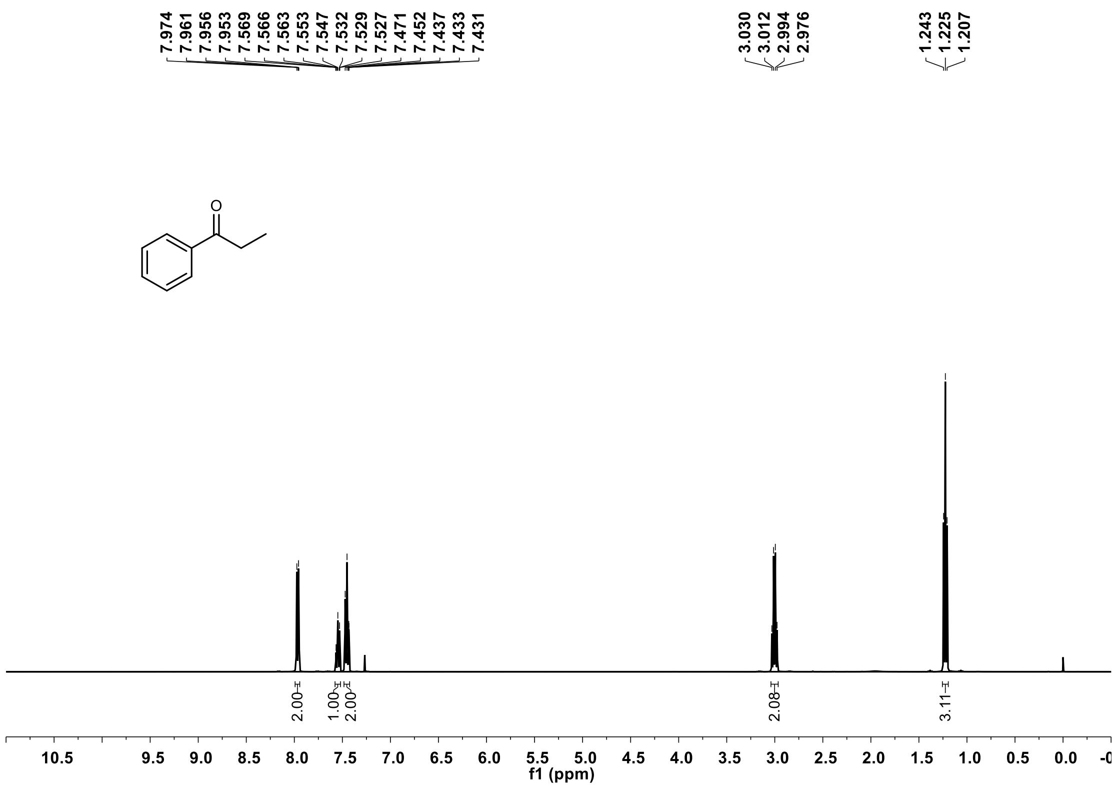

Supplementary Figure 44. **^{1}H NMR (400 MHz, $CDCl_3$) spectrum of 2bb**

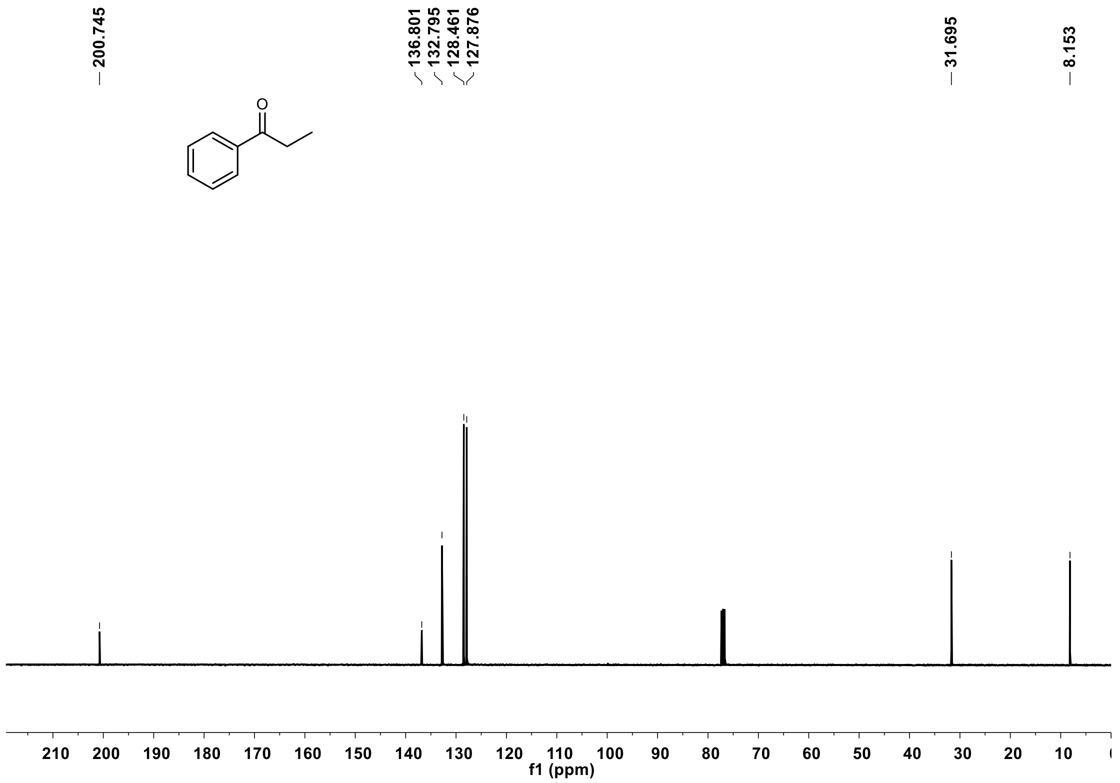

Supplementary Figure 45. **^{13}C NMR (101 MHz, $CDCl_3$) spectrum of 2bb**

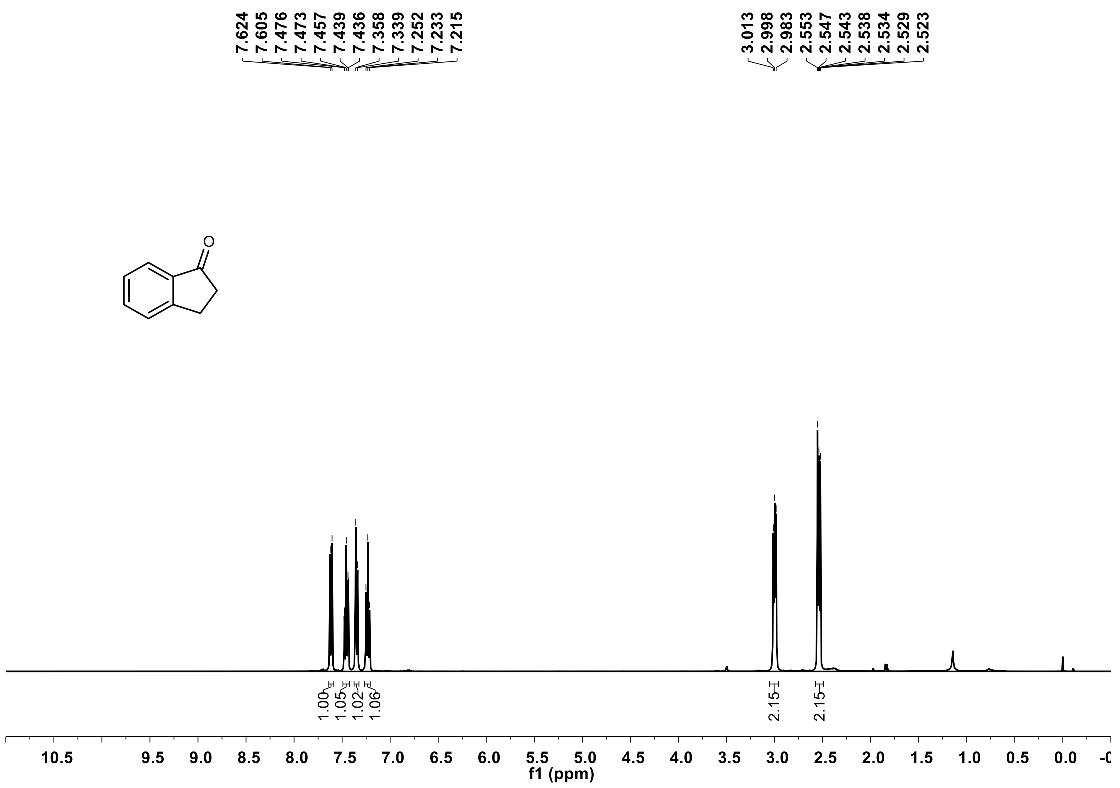

Supplementary Figure 46. ^{1}H NMR (400 MHz, $CDCl_3$) spectrum of 2bc

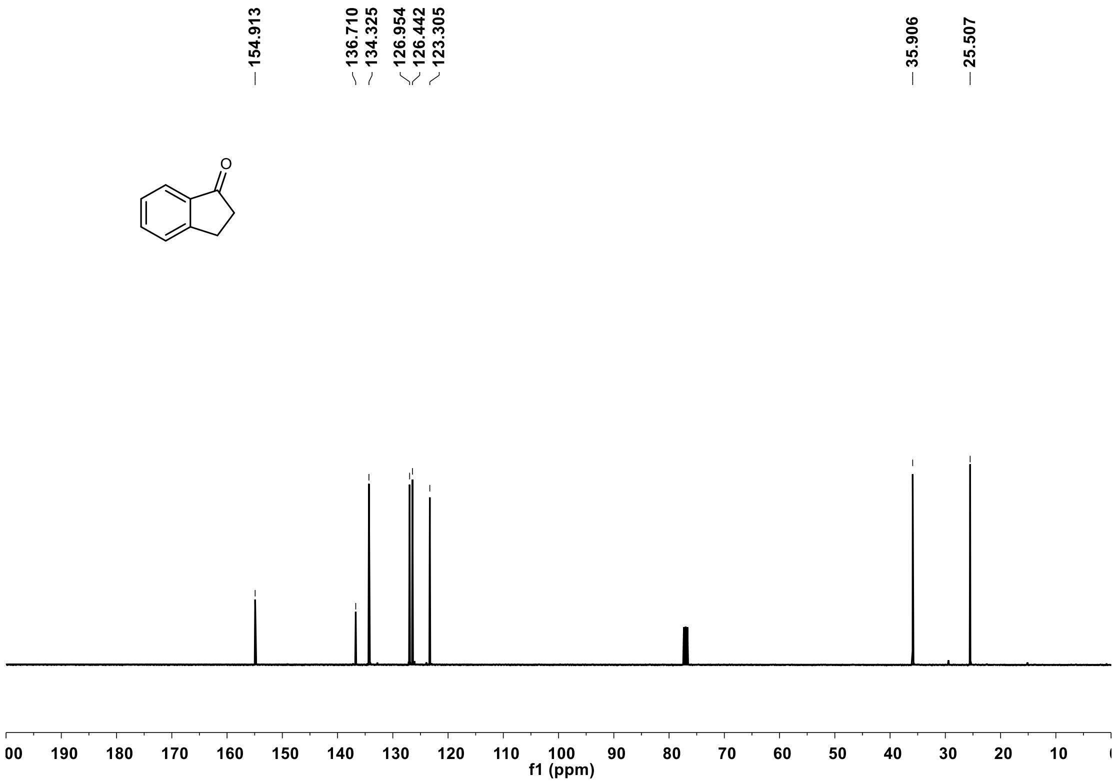

Supplementary Figure 47. ^{13}C NMR (101 MHz, $CDCl_3$) spectrum of 2bc

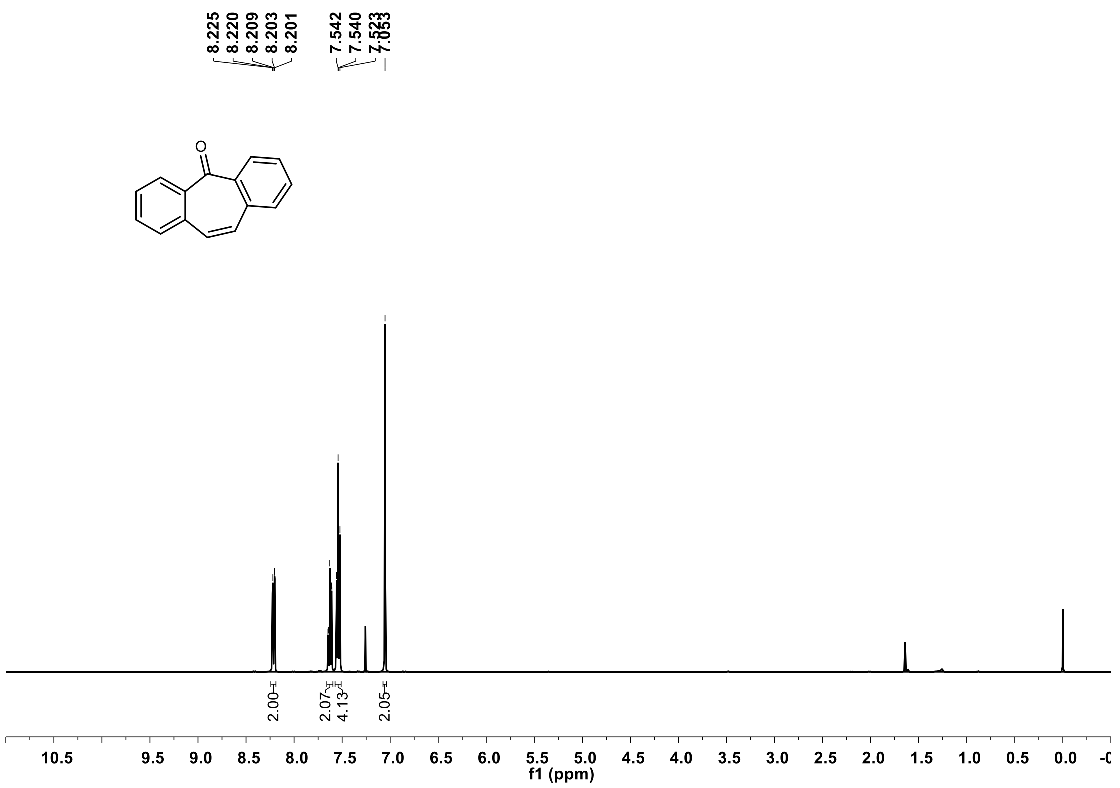

Supplementary Figure 48. ^{1}**H NMR (400 MHz,** $CDCl_3$**) spectrum of 2bd**

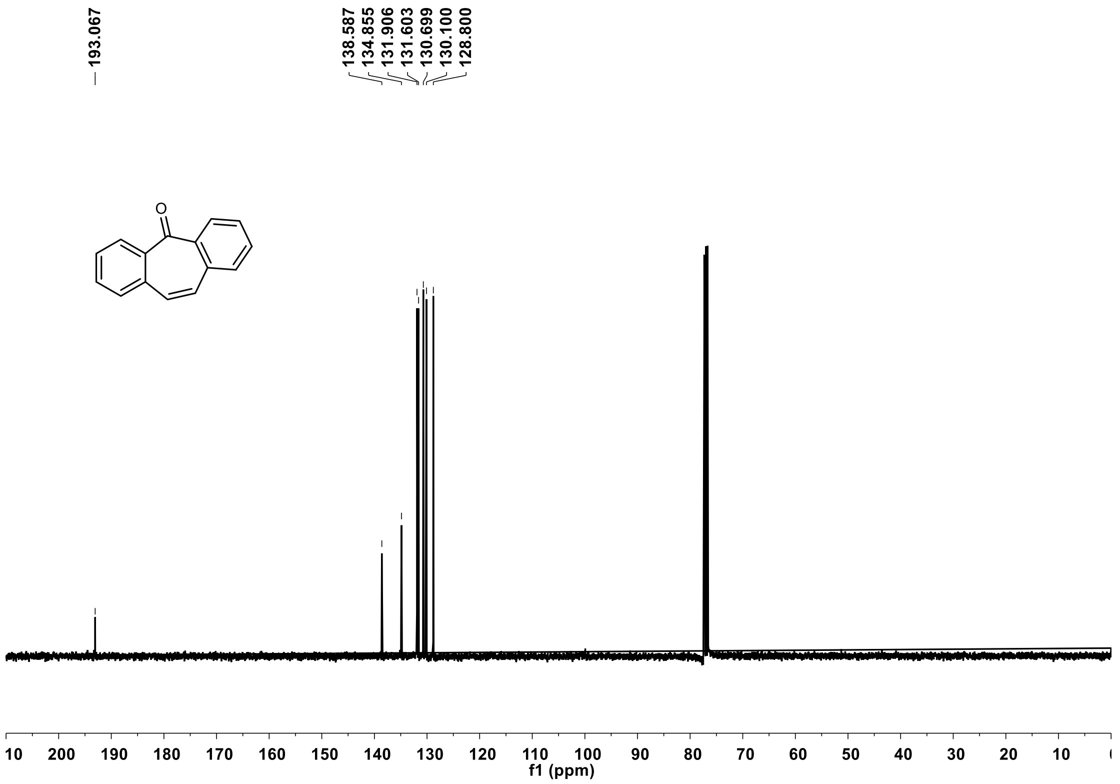

Supplementary Figure 49. ^{13}**C NMR (101 MHz,** $CDCl_3$**) spectrum of 2bd**

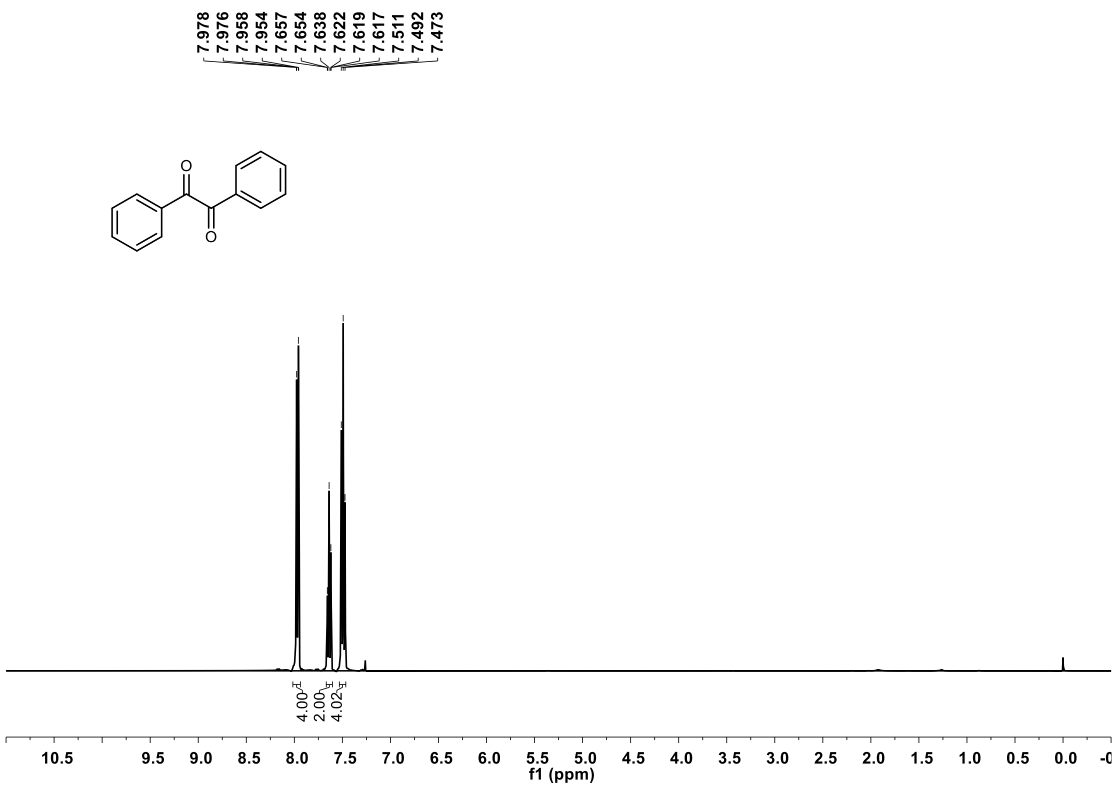

Supplementary Figure 50. ^{1}H **NMR (400 MHz, $CDCl_3$) spectrum of 2be**

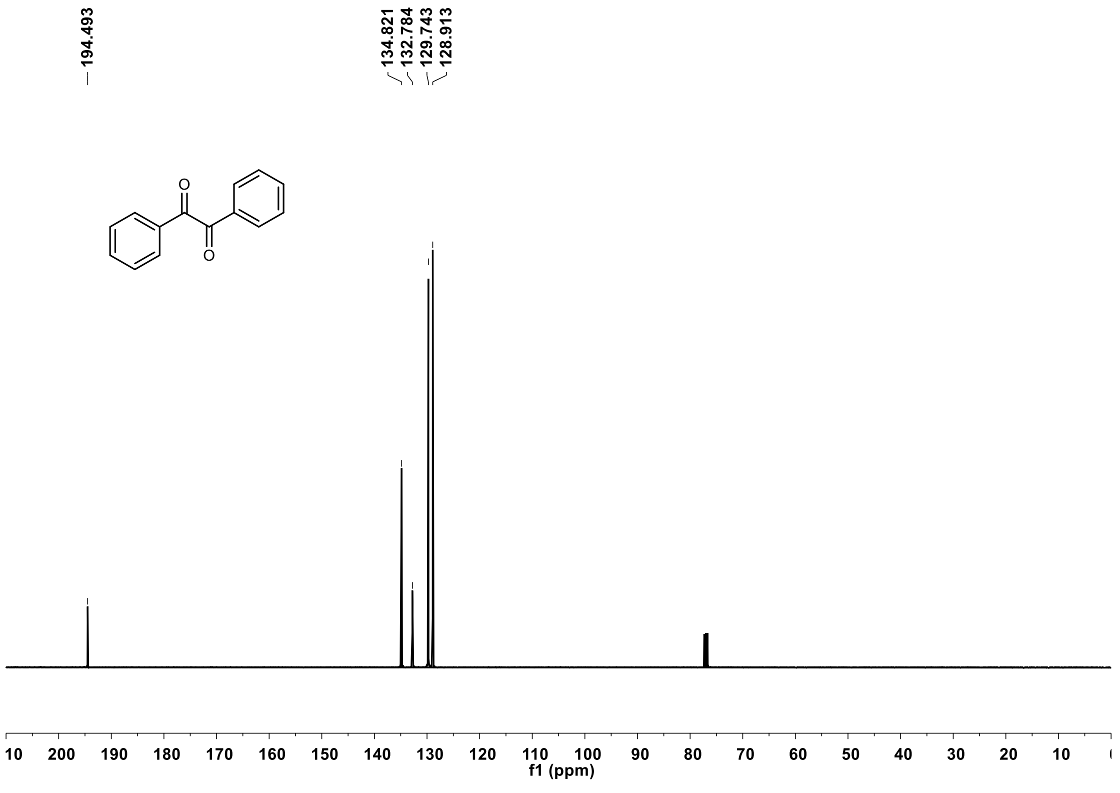

Supplementary Figure 51. ^{13}C **NMR (101 MHz, $CDCl_3$) spectrum of 2be**

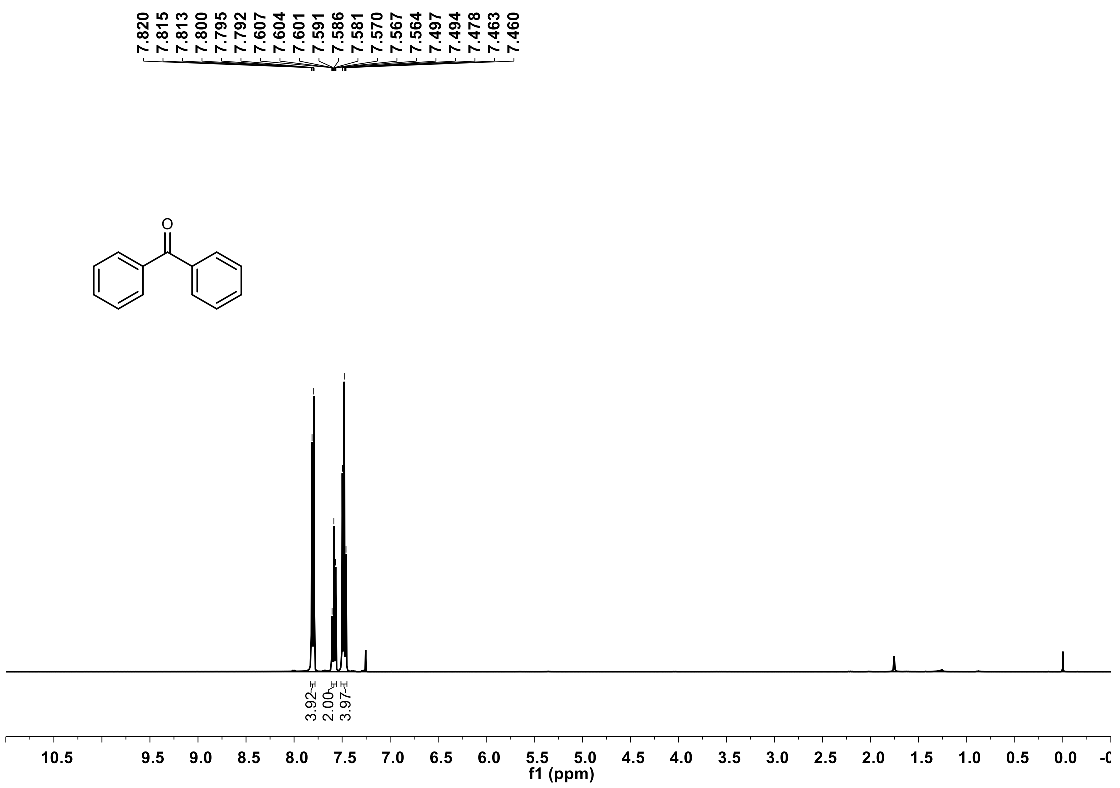

Supplementary Figure 52. ^{1}H NMR (400 MHz, $CDCl_3$) spectrum of 2bf

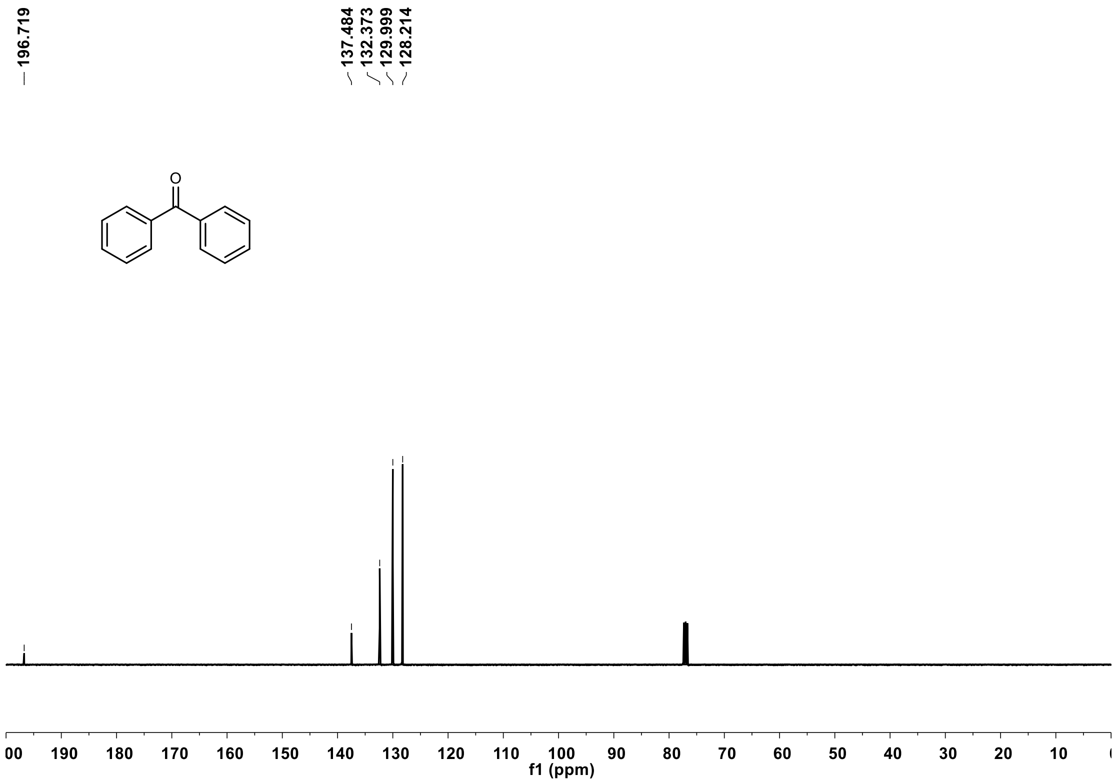

Supplementary Figure 53. ^{13}C NMR (101 MHz, $CDCl_3$) spectrum of 2bf

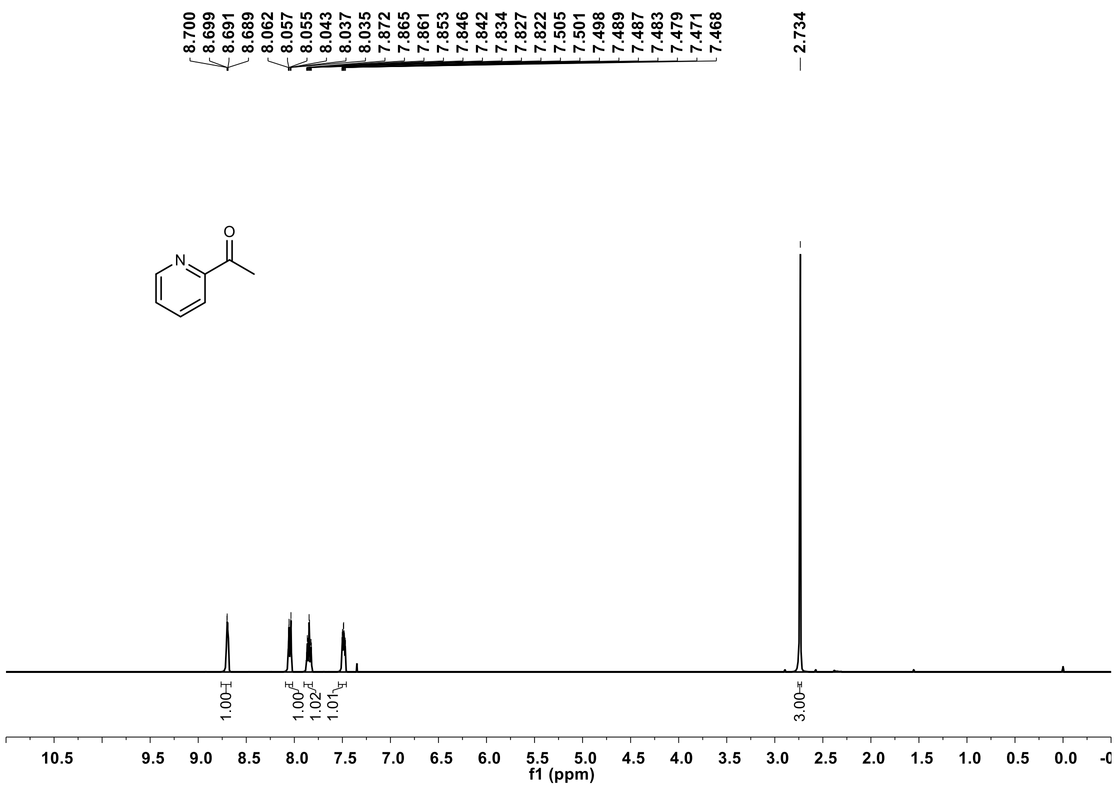

Supplementary Figure 54. ^{1}H **NMR (400 MHz,** $CDCl_3$**) spectrum of 2bg**

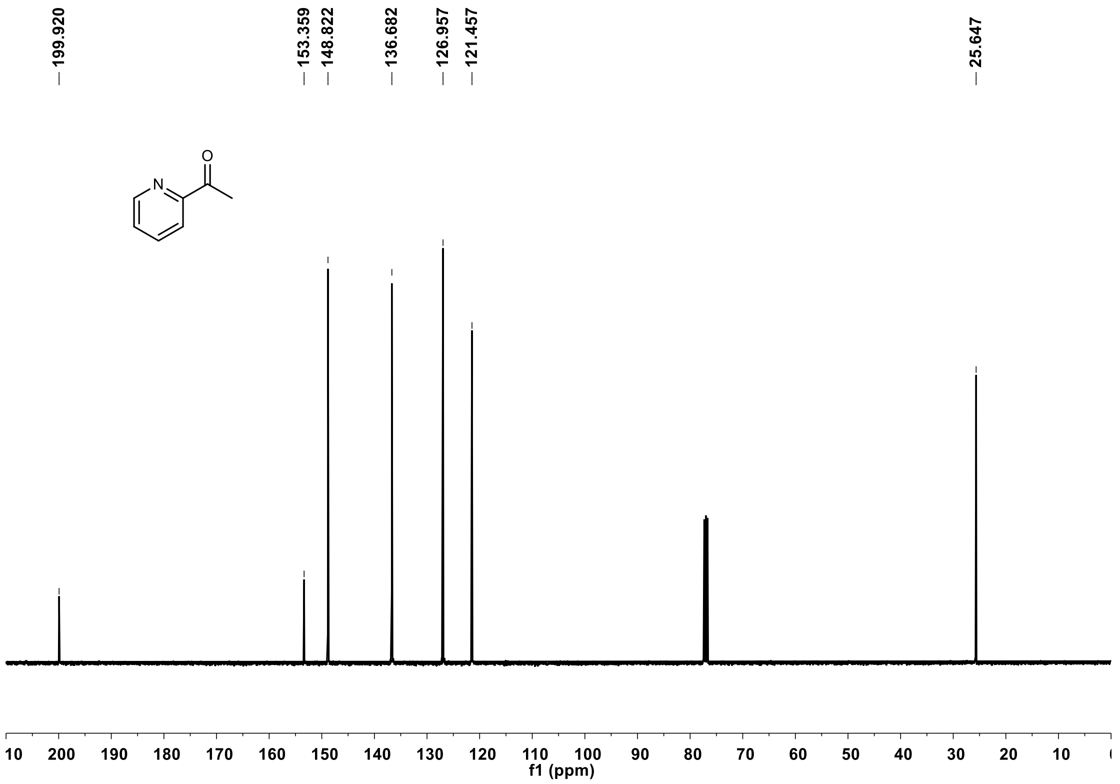

Supplementary Figure 55. ^{13}C **NMR (101 MHz,** $CDCl_3$**) spectrum of 2bg**

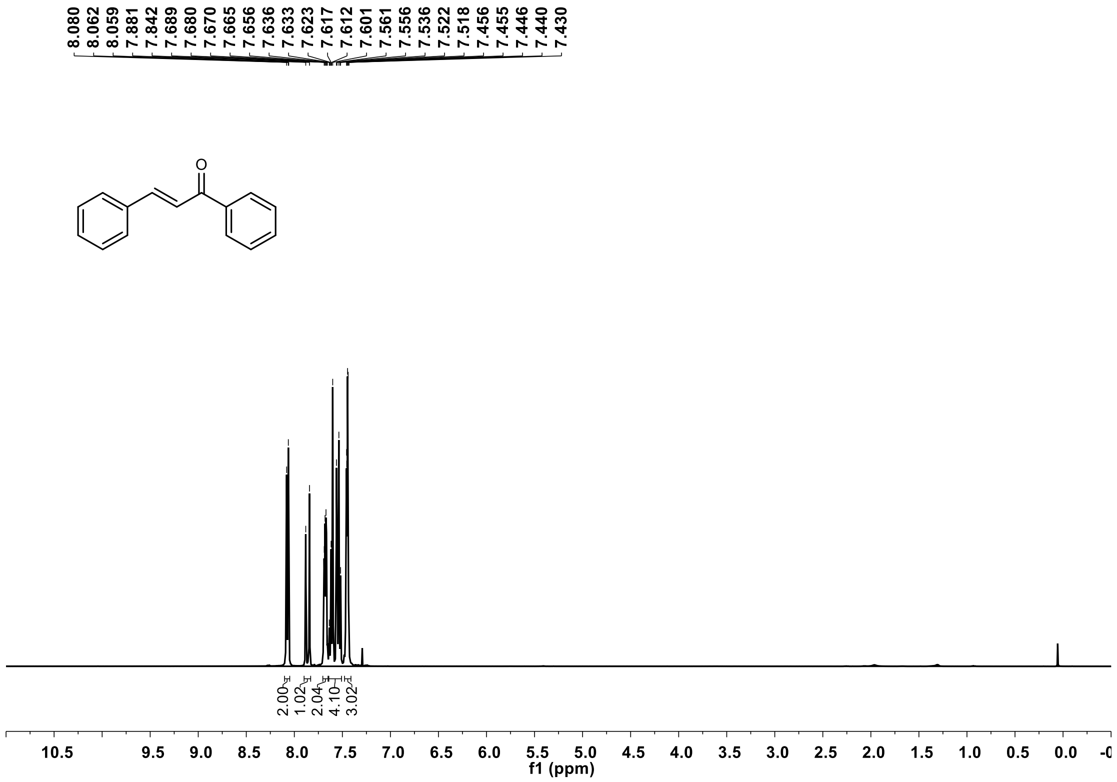

Supplementary Figure 56. ^{1}H NMR (400 MHz, $CDCl_3$) spectrum of 2bh

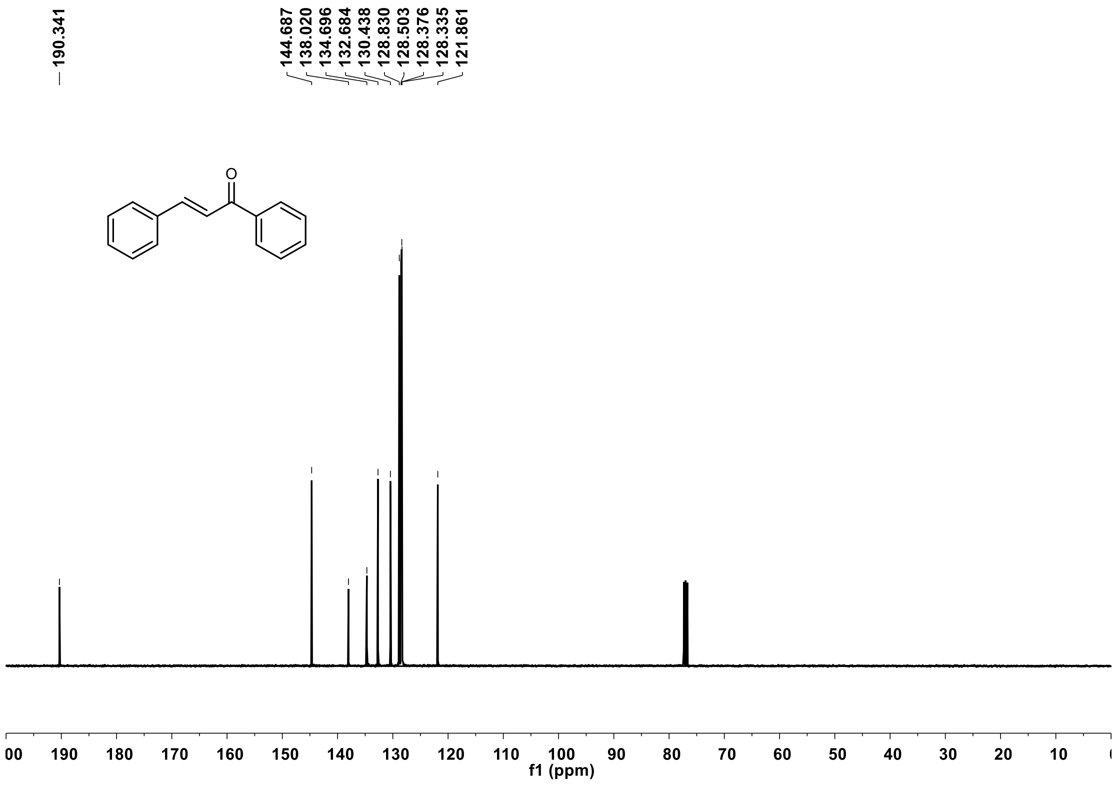

Supplementary Figure 57. ^{13}C NMR (101 MHz, $CDCl_3$) spectrum of 2bh

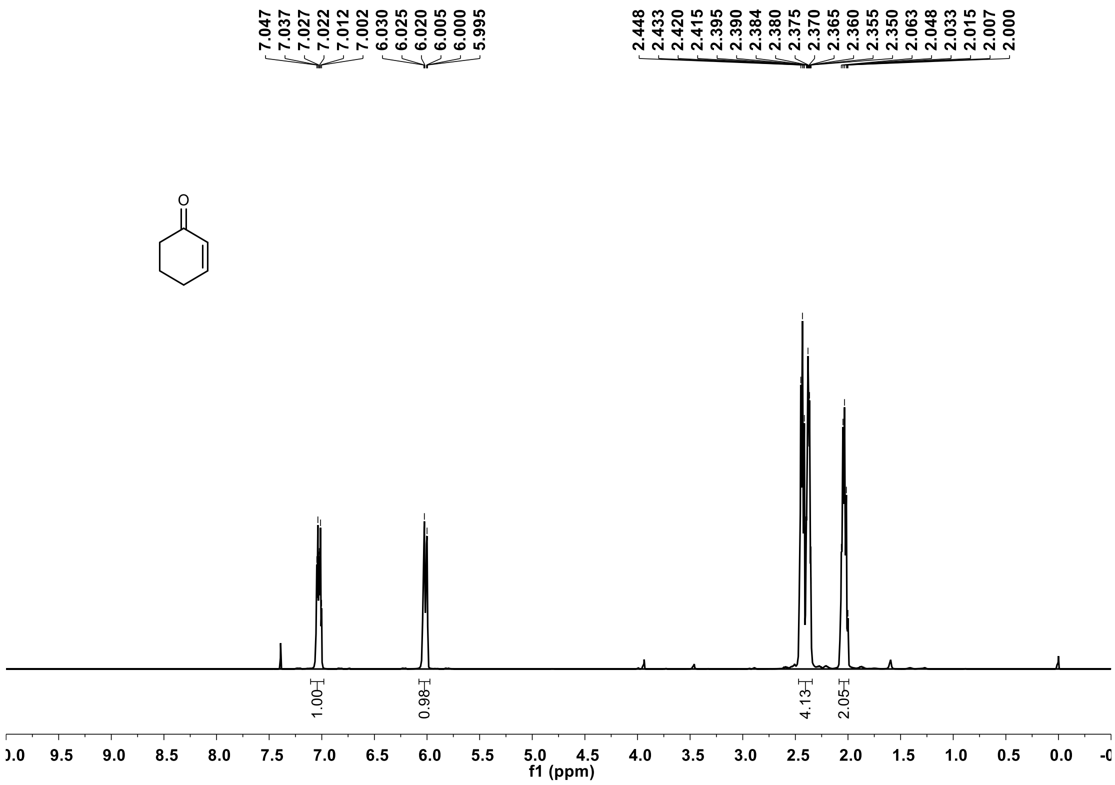

Supplementary Figure 58. ^{1}H NMR (400 MHz, $CDCl_3$) spectrum of 2bi

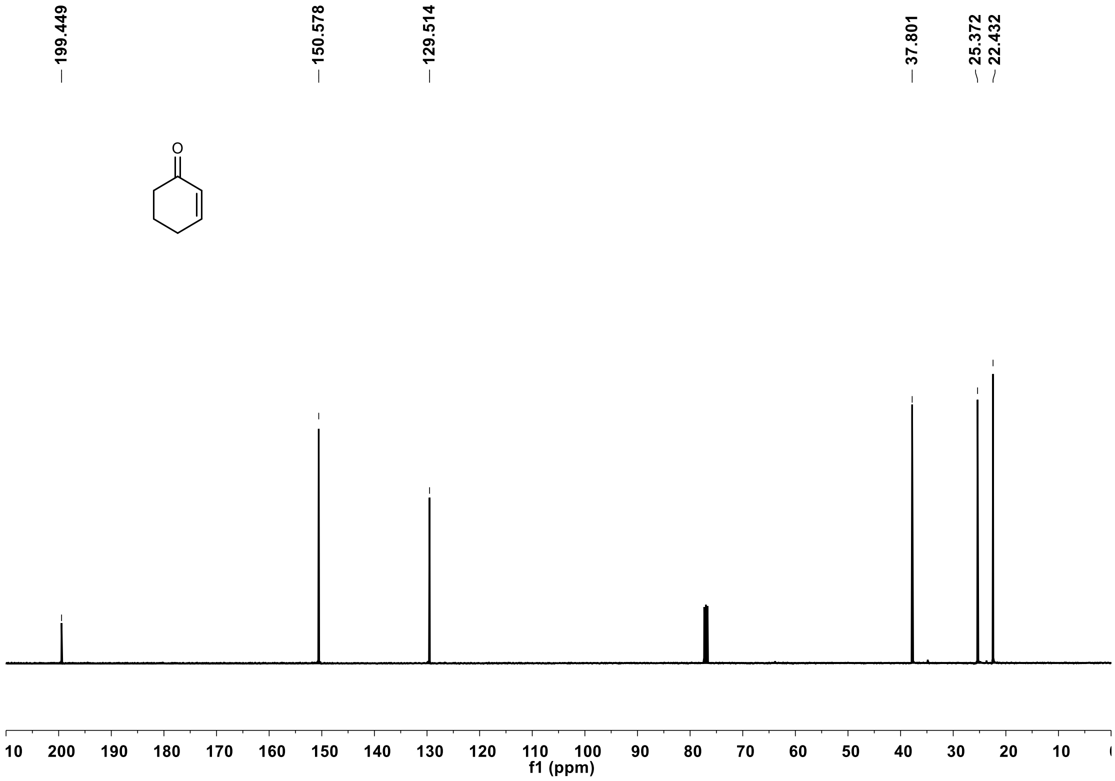

Supplementary Figure 59. ^{13}C NMR (101 MHz, $CDCl_3$) spectrum of 2bi

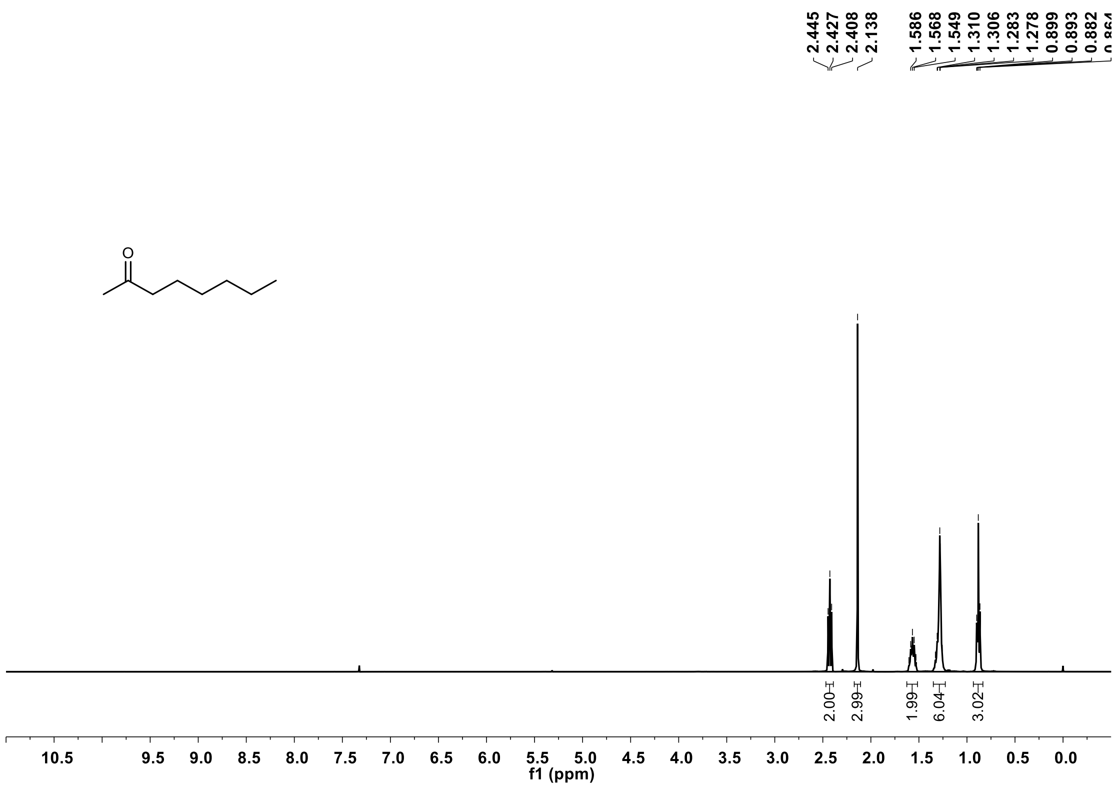

Supplementary Figure 60. ^{1}H NMR (400 MHz, $CDCl_3$) spectrum of 2bj

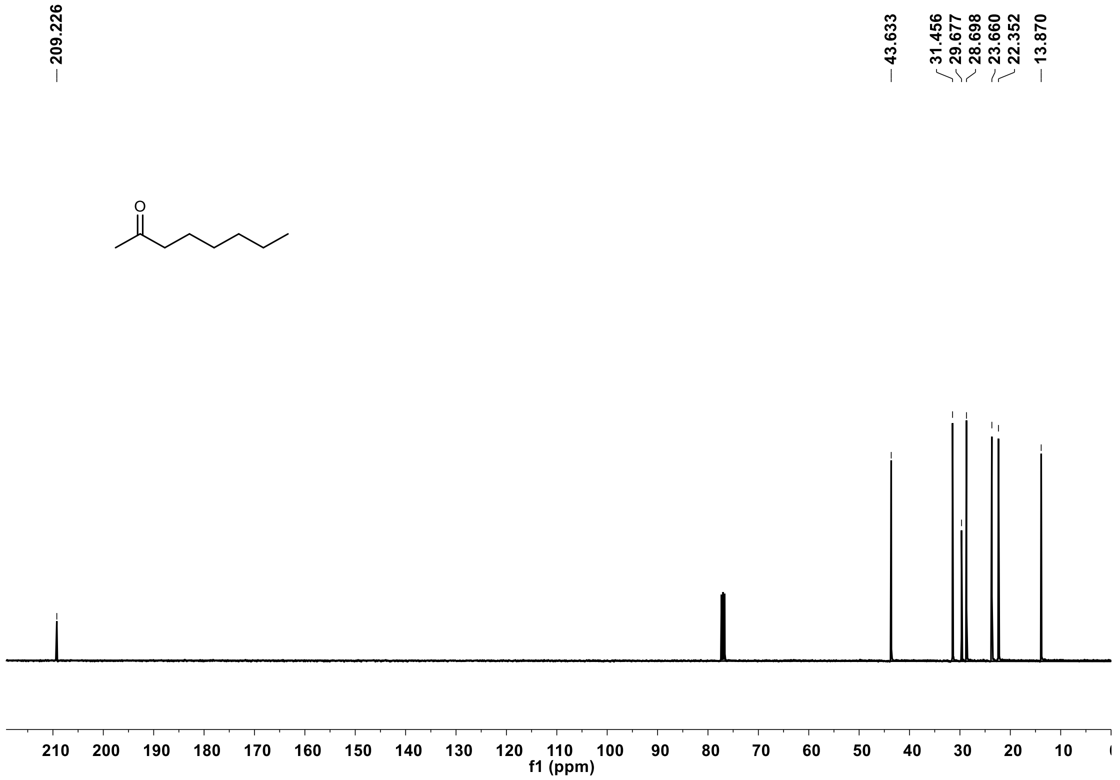

Supplementary Figure 61. ^{13}C NMR (101 MHz, $CDCl_3$) spectrum of 2bj

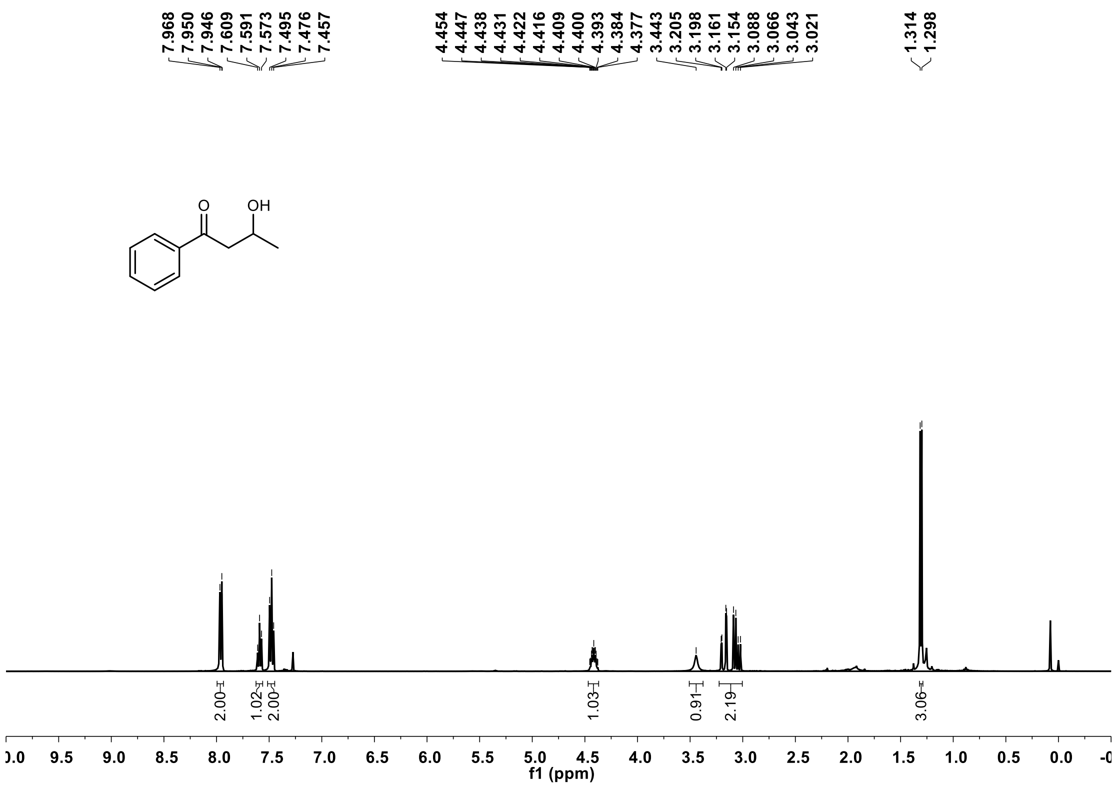

Supplementary Figure 62. **^{1}H NMR (400 MHz, $CDCl_3$) spectrum of 4**

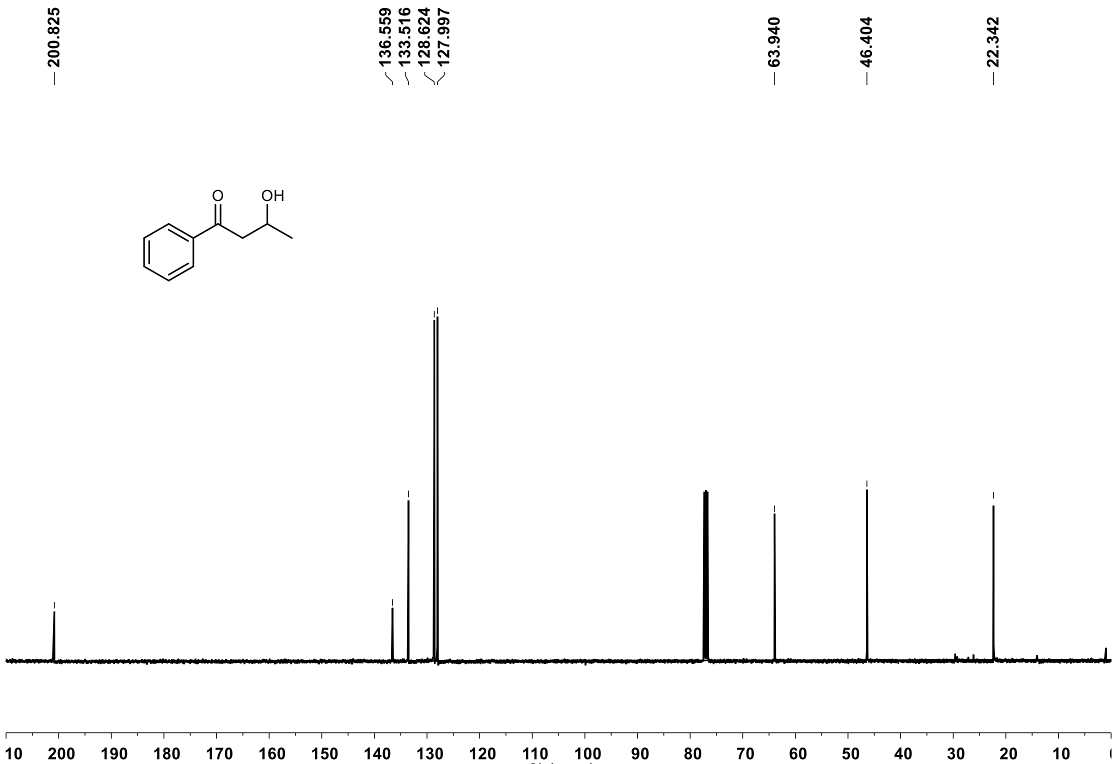

Supplementary Figure 63. **^{13}C NMR (101 MHz, $CDCl_3$) spectrum of 4**

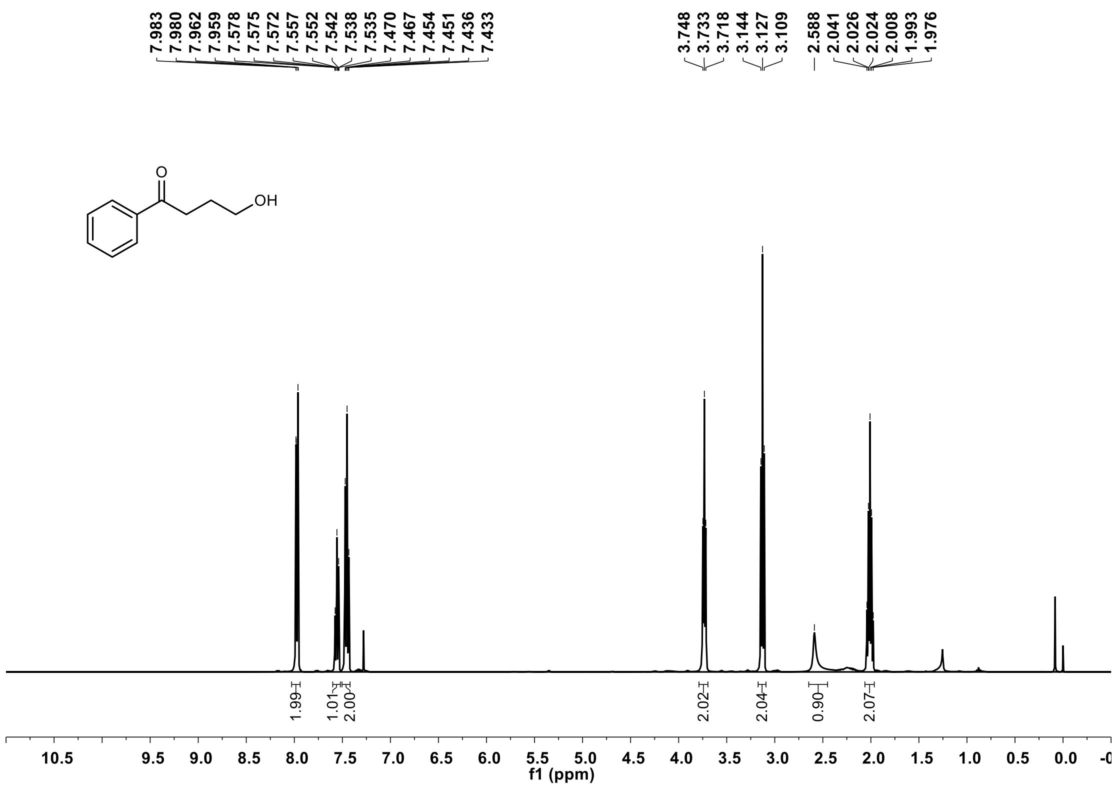

Supplementary Figure 64. ^{1}H NMR (400 MHz, $CDCl_3$) spectrum of 6

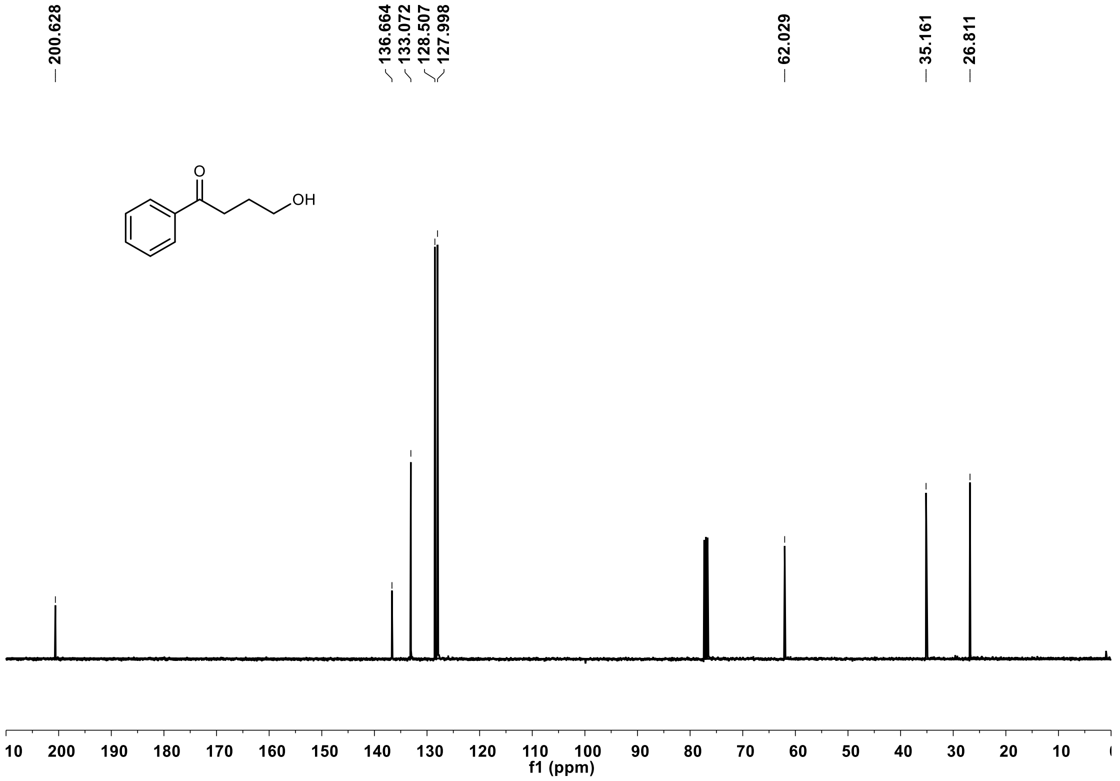

Supplementary Figure 65. ^{13}C NMR (101 MHz, $CDCl_3$) spectrum of 6

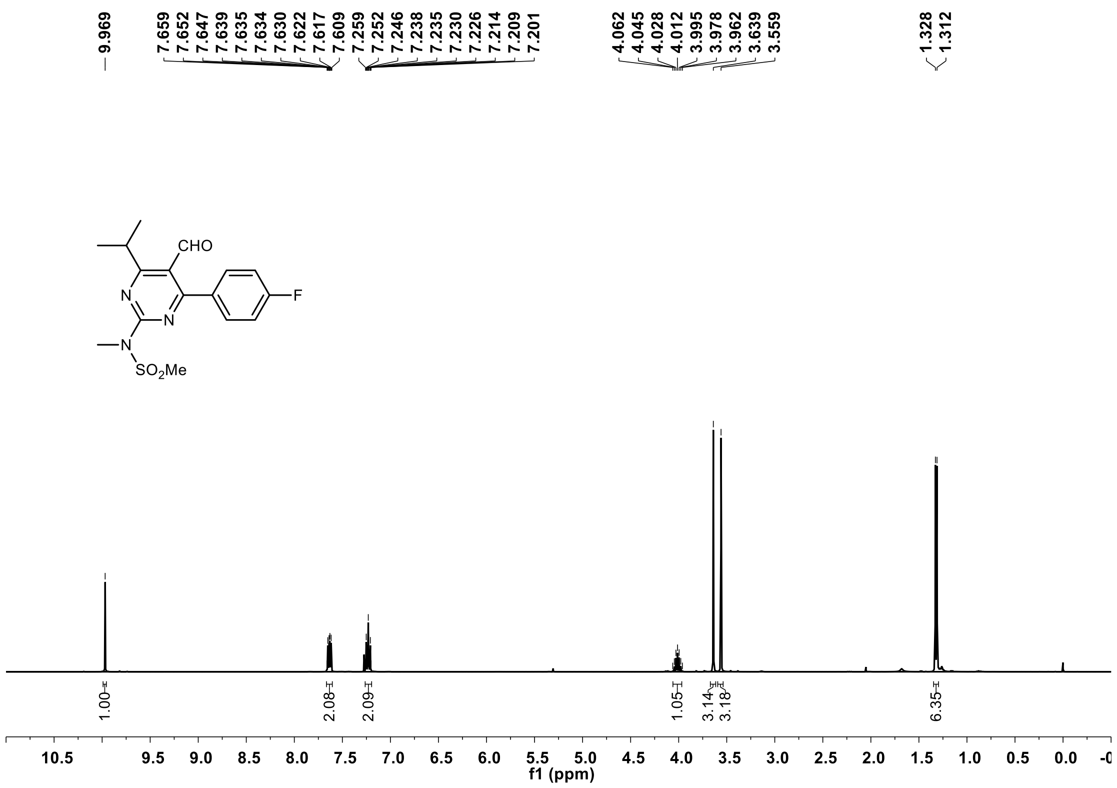

Supplementary Figure 66. ^{1}H NMR (400 MHz, $CDCl_3$) spectrum of 2aq

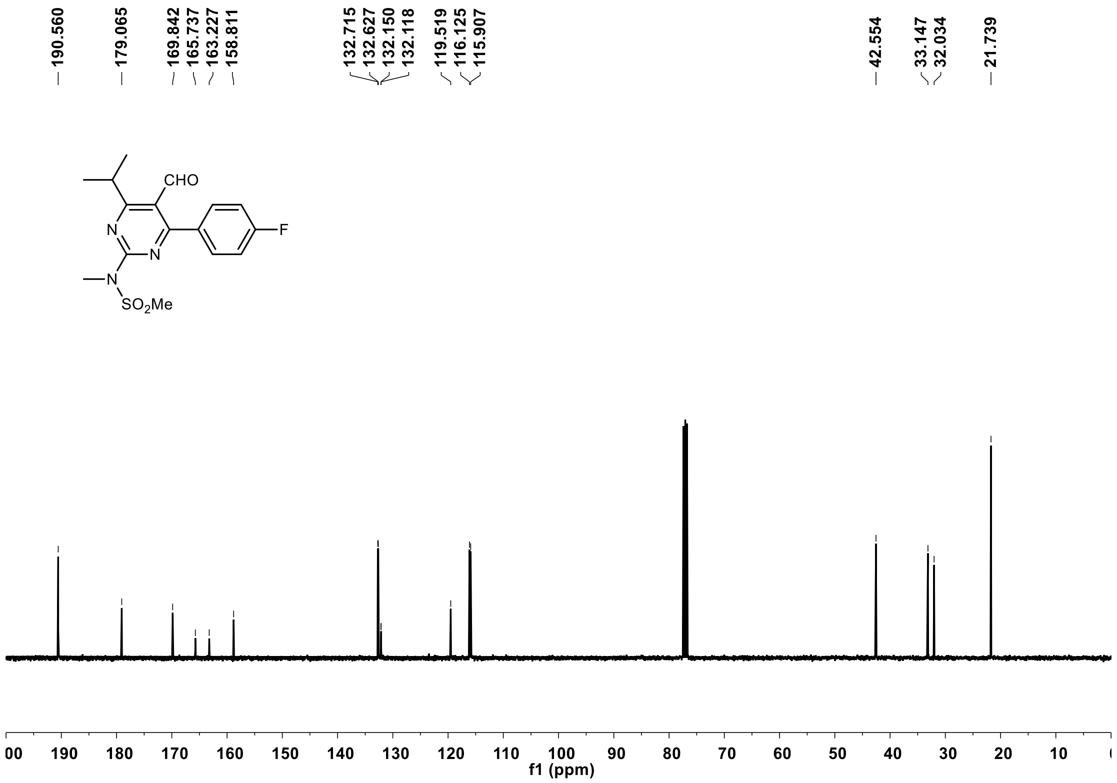

Supplementary Figure 67. ^{13}C NMR (101 MHz, $CDCl_3$) spectrum of 2aq

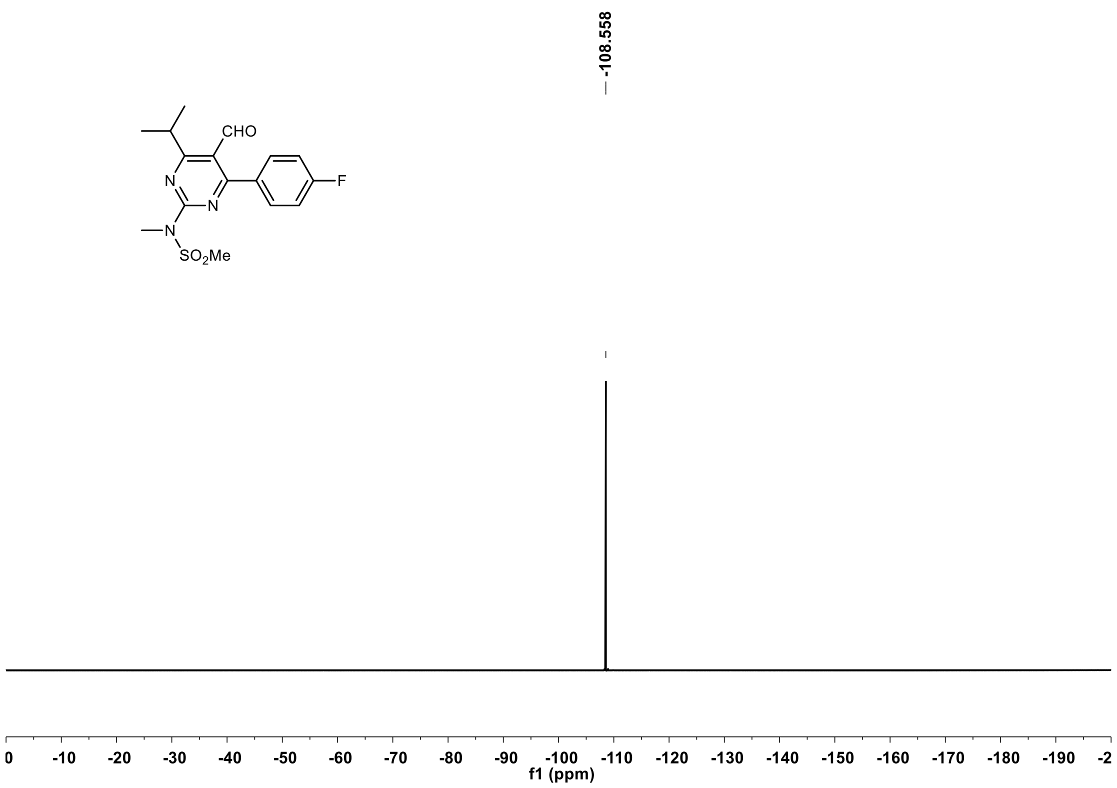

Supplementary Figure 68. ^{19}F NMR (376 MHz, $CDCl_3$) spectrum of 2aq

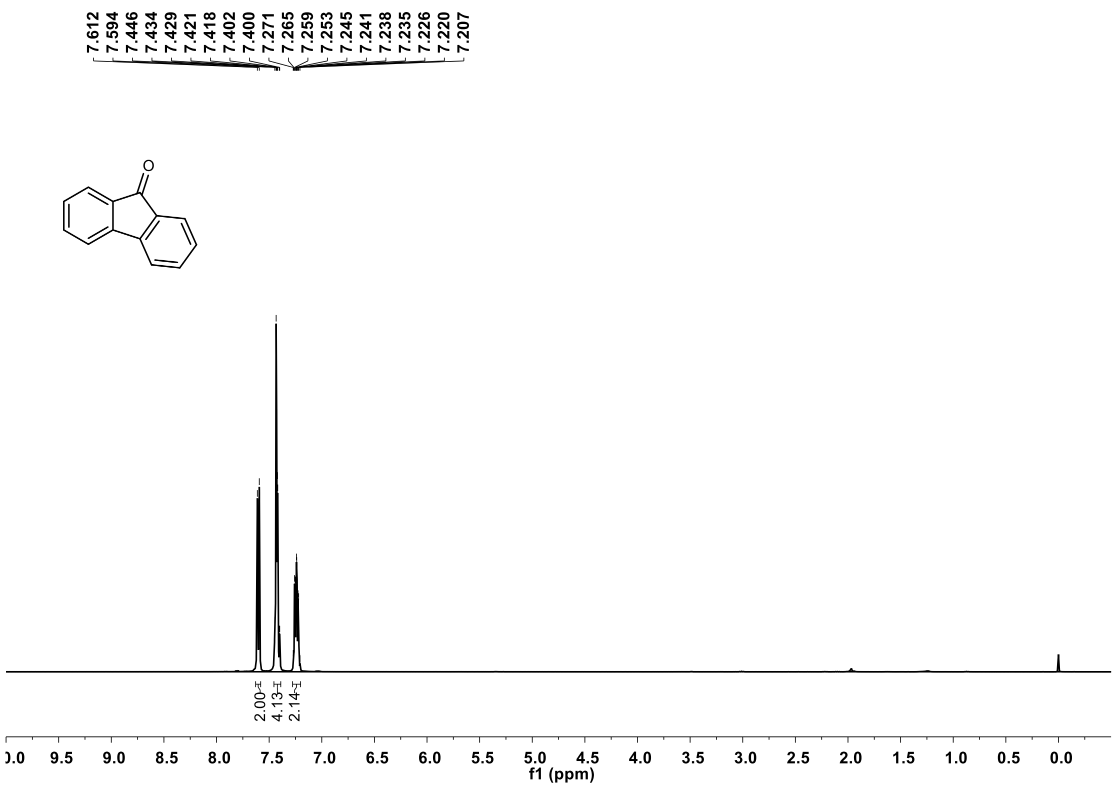

Supplementary Figure 69. ^{1}H NMR (400 MHz, $CDCl_3$) spectrum of 2bk

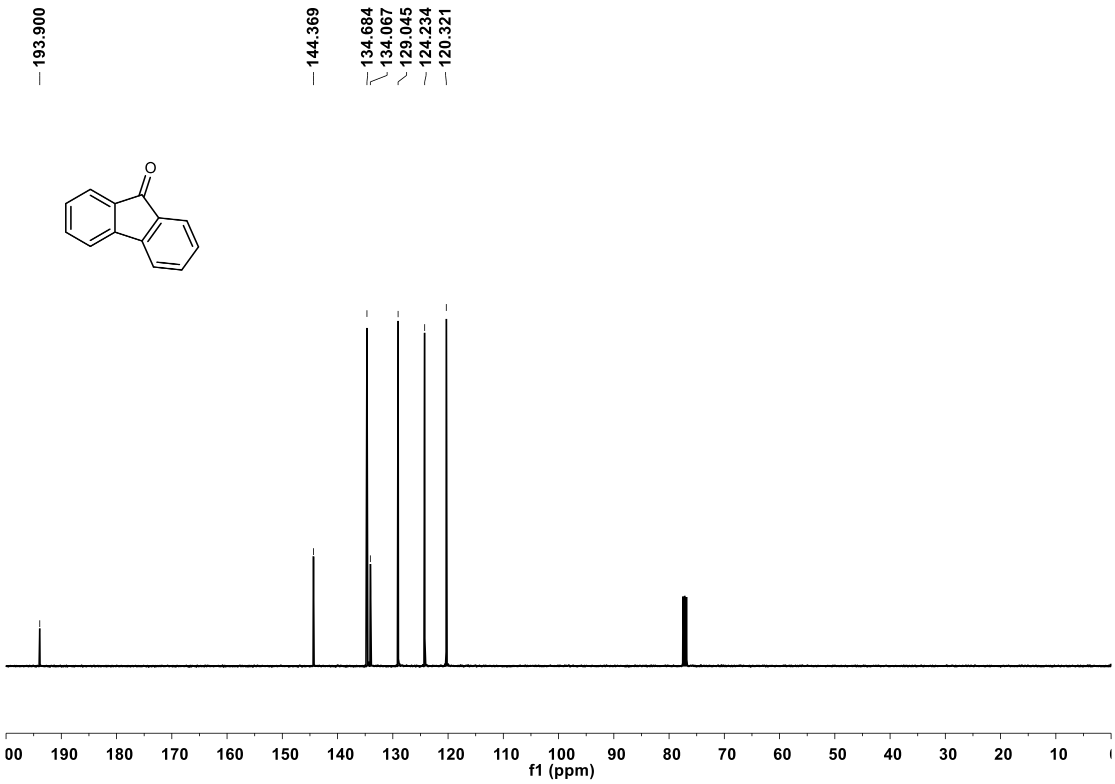

Supplementary Figure 70. ^{13}C NMR (101 MHz, $CDCl_3$) spectrum of 2bk

Supplementary References

1. Fernandes, R. A. & Bethi, V. An expedient osmium(VI)/$K_3Fe(CN)_6$-mediated selective oxidation of benzylic, allylic and propargylic alcohols. *RSC Adv.* ***4***, 40561-40568 (2014).
2. Kim, M. J., Jung, Y. E., Lee, C. Y. & Kim, J. HKUST-1/ABNO-catalyzed aerobic oxidation of secondary benzyl alcohols at room temperature. *Tetrahedron Lett.* ***59***, 2722-2725 (2018).
3. Dip, I., Gethers, C., Rice, T. & Straub, T. S. A rapid and convenient oxidation of secondary alcohols. *Tetrahedron Lett.* ***58***, 2720-2722 (2017).
4. Das, A. & Stahl, S. S. Noncovalent Immobilization of Molecular Electrocatalysts for Chemical Synthesis: Efficient Electrochemical Alcohol Oxidation with a Pyrene-TEMPO Conjugate. *Angew. Chem. Int. Ed.* ***56***, 8892-8897 (2017).
5. Zhao, L.-M., Meng, Q.-Y., Fan, X.-B., Ye, C., Li, X.-B., Chen, B., Ramamurthy, V., Tung, C.-H. & Wu, L.-Z. Photocatalysis with Quantum Dots and Visible Light: Selective and Efficient Oxidation of Alcohols to Carbonyl Compounds through a Radical Relay Process in Water. *Angew. Chem. Int. Ed.* ***56***, 3020-3024 (2017).
6. McTiernan, C. D., Pitre, S. P. & Scaiano, J. C. Photocatalytic Dehalogenation of Vicinal Dibromo Compounds Utilizing Sexithiophene and Visible-Light Irradiation. *ACS Catal.* ***4***, 4034-4039 (2014).
7. Genna, D. T. & Posner, G. H. Cyanocuprates Convert Carboxylic Acids Directly into Ketones. *Org. Lett.* ***13***, 5358-5361 (2011).
8. Kon, Y., Usui, Y. & Sato, K. Oxidation of allylic alcohols to α,β-unsaturatedcarbonyl compounds with aqueous hydrogen peroxide under organic solvent-free conditions. *Chem. Commun.* 4399-4400 (2007).
